# *Helicobacter pylori* disrupts gastric mucosal homeostasis by stimulating macrophages to secrete CCL3

**DOI:** 10.1186/s12964-024-01627-5

**Published:** 2024-05-10

**Authors:** Yan-Fei Wei, Xue Li, Meng-Ran Zhao, Si Liu, Li Min, Sheng-Tao Zhu, Shu-Tian Zhang, Si-An Xie

**Affiliations:** 1 Department of Gastroenterology, Beijing Friendship Hospital, Capital Medical University, Beijing, 100050, China; 2 National Clinical Research Center for Digestive Disease, Beijing Digestive Disease Center, State Key Laboratory of Digestive Health, Beijing, 100050, China

**Keywords:** *Helicobacter pylori*, Gastritis, Macrophage, CCL3

## Abstract

**Background:**

*Helicobacter pylori* (*H. pylori*) is the predominant etiological agent of gastritis and disrupts the integrity of the gastric mucosal barrier through various pathogenic mechanisms. After *H. pylori* invades the gastric mucosa, it interacts with immune cells in the lamina propria. Macrophages are central players in the inflammatory response, and *H. pylori* stimulates them to secrete a variety of inflammatory factors, leading to the chronic damage of the gastric mucosa. Therefore, the study aims to explore the mechanism of gastric mucosal injury caused by inflammatory factors secreted by macrophages, which may provide a new mechanism for the development of *H. pylori*-related gastritis.

**Methods:**

The expression and secretion of CCL3 from *H. pylori* infected macrophages were detected by RT-qPCR, Western blot and ELISA. The effect of *H. pylori*-infected macrophage culture medium and CCL3 on gastric epithelial cells tight junctions were analyzed by Western blot, immunofluorescence and transepithelial electrical resistance. EdU and apoptotic flow cytometry assays were used to detect cell proliferation and apoptosis levels. Dual-luciferase reporter assays and chromatin immunoprecipitation assays were used to study CCL3 transcription factors. Finally, gastric mucosal tissue inflammation and CCL3 expression were analyzed by hematoxylin and eosin staining and immunohistochemistry.

**Results:**

After *H. pylori* infection, CCL3 expressed and secreted from macrophages were increased. *H. pylori*-infected macrophage culture medium and CCL3 disrupted gastric epithelial cells tight junctions, while CCL3 neutralizing antibody and receptor inhibitor of CCL3 improved the disruption of tight junctions between cells. In addition, *H. pylori*-infected macrophage culture medium and CCL3 recombinant proteins stimulated P38 phosphorylation, and P38 phosphorylation inhibitor improved the disruption of tight junctions between cells. Besides, it was identified that STAT1 was a transcription factor of CCL3 and *H. pylori* stimulated macrophage to secret CCL3 through the JAK1-STAT1 pathway. Finally, after mice were injected with murine CCL3 recombinant protein, the gastric mucosal injury and inflammation were aggravated, and the phosphorylation level of P38 was increased.

**Conclusions:**

In summary, our findings demonstrate that *H. pylori* infection stimulates macrophages to secrete CCL3 via the JAK1-STAT1 pathway. Subsequently, CCL3 damages gastric epithelial tight junctions through the phosphorylation of P38. This may be a novel mechanism of gastric mucosal injury in *H. pylori*-associated gastritis.

**Supplementary Information:**

The online version contains supplementary material available at 10.1186/s12964-024-01627-5.

## Introduction

*Helicobacter pylori (H. pylori)*, the gram-negative helical bacterium, has a global infection prevalence exceeding 50% [1]. The virulence of *H. pylori* as well as host genetic and environmental factors collectively contribute to the development of gastric mucosal lesions [2]. All individuals infected with *H. pylori* have chronic gastritis, which may progress to peptic ulcers, atrophic gastritis, or gastric cancer [3, 4]. Consequently, the International Agency for Research on Cancer designated *H. pylori* as a Group 1 carcinogen [5, 6]. After *H. pylori* infection, gastric epithelial cells release inflammatory cytokines which recruit macrophages to the site of infection [7]. *H. pylori* invades the gastric epithelium and enters the lamina propria, then interacts with macrophages and stimulates macrophages to secrete inflammatory factors which further aggravates gastric mucosal damage [8]. Therefore, we aimed to study the mechanism by which *H. pylori* and macrophages synergize to cause the progression of gastritis.

Chronic *H. pylori* infection may be attributed to an inadequate immune response, indicating complex regulation between *H. pylori* and the immune system [9]. Macrophages, which originate from blood monocytes, play crucial roles in both innate and adaptive immunity [10]. At the site of infection, macrophages release cytokines, such as interleukin (IL), tumor necrosis factor (TNF), which contribute to the body's inflammatory response [11, 12]. A positive correlation was observed between the abundance of macrophages and the inflammatory response of the gastric mucosa following infection with *H. pylori* [13]. Macrophages infected with *H. pylori* induce inflammation-related factors to kill bacteria, such as nitric oxide (NO), which is an important defense mediator against *H. pylori* [14].

Chemokines, which are key molecules in the regulation of host immune responses, have been shown to exert significant effects on *H. pylori* infection. These effects include immune evasion, inflammation, mucosal damage repair, and even the progression of gastric cancer [15, 16]. Macrophage inflammatory protein 1α (MIP-1α), also referred to as CCL3, belongs to the chemokine CC family and is found in the supernatant of macrophages stimulated by lipopolysaccharide (LPS) [17]. CCL3 exhibits potent chemotactic activity and attracts immune cells, including monocytes, macrophages, and lymphocytes, to the site of inflammation by specifically binding to its receptors (CCR1, CCR4, and CCR5) [18]. As a chemokine family member, CCL3 plays a significant role in the progression of various diseases, such as respiratory disease and viral infections [19]. Prior investigations have shown elevated CCL3 expression in *H. pylori*-positive gastric mucosa [20, 21]. However, no study has revealed a relationship between CCL3 and *H. pylori*-related gastric mucosal damage.

Therefore, the objective of this study was to investigate the influence of the chemokine CCL3 on the gastric mucosa, which was released by *H. pylori-*infected macrophages. Furthermore, we aimed to elucidate the mechanisms about CCL3 secretion and gastric mucosa disruption. The findings of this study may provide a novel pathogenic mechanism for mucosal injury caused by *H. pylori.*

## Materials and methods

### Cells culture

Immortalized human gastric epithelial cells (GES-1) and human leukemia monocytic cells (THP-1) were purchased from iCell Bioscience Inc (Shanghai, China), and the human gastric cancer cell line MKN28 was purchased from Immortalized Cells (Xiamen, China). GES-1, THP-1, and MKN28 cells were cultured in RPMI 1640 (03.4007C, EallBio, China) supplemented with 10% fetal bovine serum (FBS, Gibco, USA) in a 37℃ incubator with 5% CO2.

### Reagents

Phorbol 12-myristate-13-acetate (PMA, P8139) was purchased from Sigma (USA). Vancomycin (V8050), trimethoprim (ST8980), amphotericin B (A8251), and cefsulodin sodium (IC2530) were purchased from Solarbio (Beijing, China). Fludarabine (HY-B0069), 2-NP (HY-W013523), Maraviroc (HY-13004), SB203580 (HY-10256), and Upadacitinib (HY-19569) were purchased from MCE (USA). CCL3 neutralizing antibody (AF270-NA) was purchased from RD (USA). Recombinant human CCL3 recombinant protein (Ag26982) was purchased from Proteintech (China), and recombinant murine CCL3 protein (250–09) was purchased from Peprotech (USA).

### *H. pylori* strains and cell co-culture

*H. pylori* bacterial strain 26695 (VacA^+^ and CagA^+^) was gifted from Peking University Third Hospital, and Sydney Strain 1 (SS1, VacA^+^, and CagA^+^) was donated by the Academy of Military Medical Sciences of the Chinese PLA. Both of these bacteria are standard strains. *H. pylori* was cultured on Columbian blood agar medium (CM0331, Oxoid, UK) containing 10% defibrous sheep blood (TX0030, Solarbio, China). The culture medium also incorporated four types of antibiotics: vancomycin (10 mg/L), trimethoprim (5 mg/L), amphotericin B (5 mg/L), and cefsulodin sodium (5 mg/L). *H. pylori* was cultured at 37℃ under a microaerobic humidified atmosphere (5% O_2_, 10% CO_2_, and 85% N_2_) produced by a microaerobic bag (C-02, Mitsubishi, Japan).

THP-1 cells were seeded into 6-well plates at a confluence of 70% (1 × 10^6^ cells per well) and treated with PMA (100 ng/mL) for 24 h to induce differentiation into M0. Bacteria were harvested from Colombian blood agar plates and resuspended in phosphate-buffered saline (PBS, P1020, Solarbio, China), and the concentration of *H. pylori* was estimated by spectrophotometry at OD_530_nm, with a conversion factor of 1 OD_530 _= 2 × 10^8^ CFU/mL. The cells were incubated with *H. pylori* at a multiplicity of infection (MOI) of 1:10. After 24 h, the *H. pylori* co-culture medium was collected, centrifuged at 3000 rpm for 10 min, and then filtered with a 0.22 μm filter membrane.

### RNA extraction and real-time quantitative PCR (RT-qPCR) assay

Total RNA was extracted from cells and tissues using the TRIzol reagent (15596018, Ambion, USA). Total RNA was quantified using the Biospec-Nano system. Thereafter, 1000 ng RNA was reverse-transcribed into cDNA using the PrimeScript™ RT Master Mix (RR036A, Takara, Japan) in a 20 μL volume. Then, cDNA was amplified with the Fast SYBR Green Master Mix (A25742, Thermo Fisher, USA) in a 10 μL volume. RT-qPCR was performed using the 7500 Fast Real-Time PCR System. GAPDH was used as an endogenous reference, and the relative amounts of mRNA were determined based on 2^−ΔΔCt^ calculations. Primers were synthesized by Sangon Biotech (Shanghai, China), and the sequences were listed in Supplementary Table 1.

### Protein extraction and Western blot (WB)

Proteins were extracted from cells or tissues using RIPA lysis buffer (P0013C, Beyotime, China) containing phosphatase inhibitor (CW2383S, CWBIO, China) and protease inhibitor cocktail (04693132001, Roche, Switzerland). The protein concentrations were determined using a BCA kit (BL521A, Biosharp, China), and bovine serum albumin (BSA) inside the kit was used as the protein standard. Equivalent amounts of protein (30 μg) were subjected electrophoresis on 10% (PG112) or 12% (PG113) sodium dodecyl sulfate polyacrylamide gels (Epizyme, China) and transferred to 0.22 μm polyvinylidene fluoride (PVDF) membranes (ISEQ00010, Immobilon, USA). The membranes were blocked using 5% non-fat milk in TBST buffer at room temperature for 2 h and then incubated with primary antibodies overnight at 4℃. The membranes were incubated with HRP-conjugated secondary antibodies at room temperature for 1.5 h. Protein bands were imaged and quantified using the ChemiDoc XRS + system (Bio-Rad, USA). The antibodies used were listed in Supplementary Table 2.

### Cell proliferation and apoptosis assays

Cell proliferation was determined by Cell Counting Kit-8 (CCK8) and 5-ethynyl-2-deoxyuridine (EdU) assays. For the CCK8 assay, GES-1 and MKN28 cells were seeded in different conditioned medias at a density of 2000 cells per well in 96-well plates. Every 24 h for 72 h, 10 μL of the CCK8 kit reagent (C0038, Beyotime, China) was added to each well, and cell viability was measured with spectrophotometry at OD_450_nm after 2 h of incubation. The EdU assay was performed using the Cell-Light™ EdU Apollo^®^ 567 kit (C10310-1, Ribobio, China). The cells were seeded into 24-well culture plates and treated with different medias or drugs, after which they were incubated with EdU for 2 h. Apollo and Hoechst staining solutions were used to observe EdU-positive cells according to the manufacturer’s instructions. Apoptosis was evaluated by FITC Annexin V Apoptosis Detection Kit (C1062L, Beyotime, China). The cells were stained with FITC-Annexin V and propidium iodide (PI) according to the manufacturer’s protocol. The apoptosis rate was detected by fluorescence-activated cell sorting (FACS) after 15 min of staining.

### Immunofluorescence (IF) and transepithelial electrical resistance (TEER)

The cells were grown on coverslips and treated with different medias or drugs before washing with PBS. The cells were then fixed in 4% paraformaldehyde, permeabilized with 0.3% Triton X-100, and blocked with 1% BSA (A8020, Solarbio, China) for 1 h at room temperature. The cells were stained with primary antibodies overnight at 4℃. The next day, the secondary antibody conjugated to Alexa Fluor^®^ 488 or Alexa Fluor^®^ 594 was added and incubated for 1 h at room temperature. Finally, the nuclei were stained with DAPI (sc3598, Santa Cruz, USA), and the cells were observed under a confocal microscope (Olympus FluoView™ FV1200, Japan). The MKN28 cells were cultured on 1.13 cm^2^ Transwell supports (14212, LABSELECT, China). TEER was measured using the transmembrane cell resistance meter (RE1600, KINGTECH, China). TEER was calculated by multiplying ohm by the surface area of the monolayer. The resistance of the transwell membrane without cells was subtracted as a blank.

### Dual-luciferase reporter assay

The pGL3 plasmid carrying the CCL3 promoter region (from -2000 base pairs to the transcription start site (TSS)) and the luciferase reporter gene were constructed by YouBio (Changsha, China). The pRL-TK plasmid which carried renilla luciferase and pGL3 plasmid carried the CCL3 promoter region were co-transfected into THP-1 cells at approximately 70% confluence using MegTran (T210003, Origene, USA). After 12 h, Fludarabine (5 μM), which is the STAT1 phosphorylation inhibitor, or 2-NP (45 μM), which is used to enhance STAT1 transcription, were added separately to the cells. Luciferase activity was assessed according to the standard protocol of the Promega Dual Luciferase Assay System (E1910, Promega, USA) after 24 h stimulation. Luciferase activity values were normalized by the corresponding renilla luciferase activity.

### Chromatin immunoprecipitation (ChIP)

ChIP was performed using a ChIP kit (P2078, Beyotime, China). After co-culture with *H. pylori* for 24 h, THP-1 cells in 10 cm^2^ plates (approximately 6 × 10^6^ cells) were used for the ChIP experiment according to the manufacturer’s protocol. After sonication, each sample was analyzed using DNA markers and agarose gel electrophoresis. The ideal bands were concentrated between 200 and 1000 base pairs. Immunoprecipitation was performed using the STAT1 antibody (1:100, 9172, CST, USA), and the corresponding input and IgG (A7016, Beyotime, China) groups were setted. The enrichment level of the CCL3 promoter was analyzed by PCR using the primers listed in Supplementary Table 1. The enrichment relative to input was calculated using the following formula: 100% × 2^^ [CT value (Input−4.32−IgG or STAT1)]^ to reflect the binding strength of the target protein in the CCL3 promoter region.

### Enzyme-linked immunosorbent assay (ELISA)

Human CCL3 ELISA kit was purchased from CUSABIO (CSB-E04662h, China). Mouse CCL3 and inflammatory factors ELISA kits were purchased from ABclonal (China). Mouse blood was collected from the periocular venous plexus and centrifuged to obtain serum. The assay was performed according to the manufacturer’s instructions. In brief, standard and serum samples or co-culture medium were added to a microplate well coated with antibodies and sequentially incubated with different reagents. Absorbance values at 450 nm were determined and calculated the samples concentration from the standard curve.

### Chemokine antibody arrays

In this study, the Proteome Profiler Human Cytokine Array Kit (ARY005B, RD, USA) was used to detect the expression of cytokines in *H. pylori*-infected macrophage culture medium. The assay was performed according to the manufacturer’s instructions. In brief, the membranes were blocked and then incubated with antibody-sample mixture overnight at 4℃. The membranes were incubated with secondary antibody. The membranes were imaged and quantified using the ChemiDoc XRS + system. Semi-quantitative analysis was performed by grayscale statistics.

### Patients and samples

Hematoxylin and eosin (H&E) stained slides were obtained from the Department of Pathology of Beijing Friendship Hospital, while gastric mucosal biopsy specimens for H&E staining were obtained from patients undergoing endoscopy at the Endoscopy Center of Beijing Friendship Hospital. The patients were divided into *H. pylori* infection group and non-infection group according to the histopathology and pathology reports. The human serum was obtained from the Department of Gastroenterology of Beijing Friendship Hospital, and the patients were checked for *H. pylori* infection by ^13^C breath test. Patients’ information is listed in Supplementary Table 3. The human gastric mucosal tissues used for immunohistochemistry (IHC) staining were obtained from the tissue chips, which were purchased from Outdo Biotech Company (Shanghai, China). These tissues are stained with *H. pylori* immunohistochemistry to determine the presence of bacterial infection. This study was approved by the Institutional Ethics Committee of Beijing Friendship Hospital (2018-P2-058–05).

### Animal experiments

Eight-week-old male C57BL/6 mice were purchased from Charles River (China). Mice were intragastrically inoculated with 0.2 mL *H. pylori* strain SS1 suspension (10^9^ CFU/mL) every other day three times (*n* = 13). Meanwhile, the other mice were inoculated with an equal volume of PBS as controls (*n* = 4). Before each inoculation, the mice were fasted for 6 h. The mice were sacrificed 6 weeks after *H. pylori* infection. One group of mice were intraperitoneally injected with recombinant murine CCL3 protein (100 ng/day, *n* = 8), while another group of mice were injected with CCL3 protein and P38 phosphorylation inhibitor together (SB203580, 5 mg/kg/day, *n* = 8). The rest mice were intraperitoneally injected with DMSO as controls (*n* = 4). Mice were injected continuously for 5 days and sacrificed on the fifth day after injection. All animal experiments were approved by the Animal Care and Use Committee of the Capital Medical University (AEEI-2023–202).

### H&E staining and IHC staining

The mice gastric tissues were fixed in 4% paraformaldehyde for 24 h, immersed in serial alcohol dehydration solutions, and embedded in paraffin. Subsequently, tissue sections (4 μm) were cut, stained with hematoxylin and eosin, and assessed under the light microscope (Olympus, Japan) or Zeiss microscope (Germany). After incubation at 65℃ for 2 h, the slides were deparaffinized in xylene and then rehydrated in alcohol. Following antigen removal under high pressure, endogenous peroxidase activity was blocked with 3% H_2_O_2_ for 10 min. Sections were blocked by goat serum for 1 h and then incubated at 4℃ overnight with the primary antibody. After incubation with the secondary antibody for 1.5 h at room temperature, the tissues were stained by the diaminobenzidine kit (DAB, ZLI-9019, ZSGB-BIO, China). Finally, the gastric tissues were counterstained with hematoxylin and photographed under the light microscope.

### Bioinformatic analysis

RNA expression microarray data were retrieved from the Gene Expression Omnibus (GEO), and Kyoto Encyclopedia of Genes and Genomes (KEGG) pathway analysis of *H. pylori* infection-related genes was carried out using the STRING website (https://cn.string-db.org/) [22, 23]. For immune cell infiltration analysis, the online analytical tool Cell type Identification By Estimating Relative Subsets Of RNA Transcripts (CIBERSORTx, https://cibersortx.stanford.edu/) [24] was used to gauge the immune response of 22 immune cells. Based on the CIBERSORTx algorithm and the LM22 gene signature, we quantified the immune response of the 22 immune cells under *H. pylori* infection based on dataset and then compared the immune response of these cells between the *H. pylori* negative and positive groups. JASPAR (https://jaspar.genereg.net/) and the transcription factor human (TF-human) database (http://bioinfo.life.hust.edu.cn/HumanTFDB#!/) were used to predict the transcription factors for CCL3. THE HUMAN PROTEIN ATLAS database (https://www.proteinatlas.org/) was used to analyze the distribution of the chemokine CCL3 in different cell types and gastric tissue.

### Statistical analysis

All statistical analyses were performed using GraphPad Prism 9.0.0. Data were expressed as the mean ± SD. Significant differences between groups were analyzed by t-test or one-way analysis of variance (ANOVA) followed by Duncan’s multiple comparison test. Statistical significance was considered at *P* < 0.05.

## Results

### Macrophages play an important role in mucosal inflammation caused by *H. pylori*

**Fig. 1:**
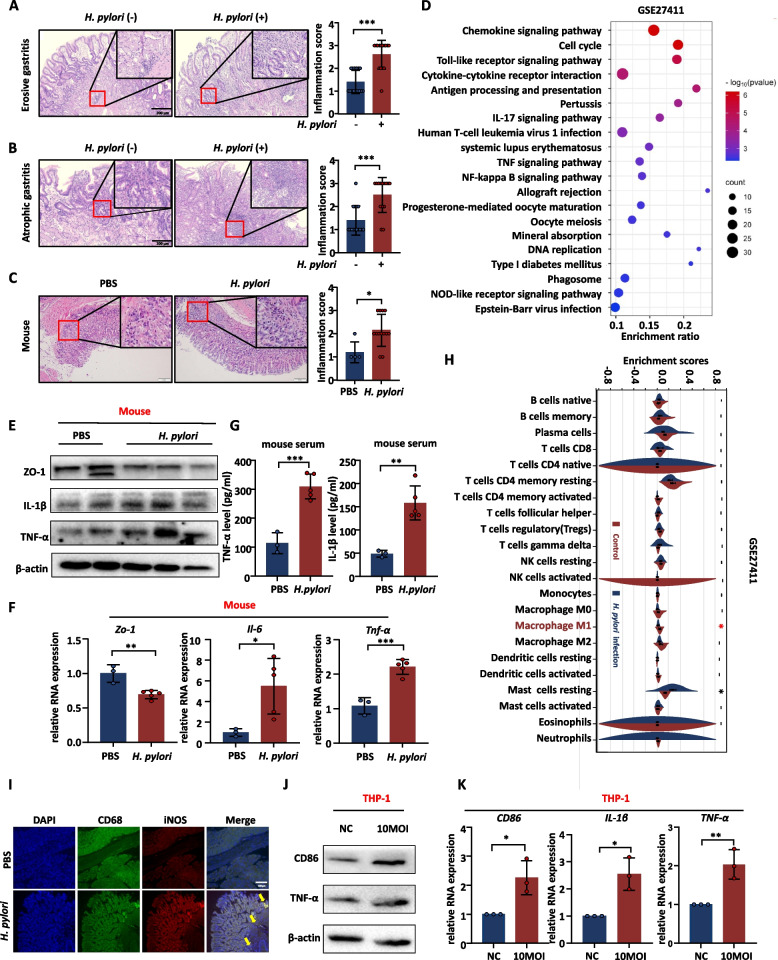
*H. pylori* infection exacerbates gastric mucosal inflammatory response and M1 macrophage infiltration. **A**, **B** Gastric mucosal H&E staining of human infected and non-infected with *H. pylori* (each group: *n* = 15). **C** Gastric mucosal H&E staining of mice infected and non-infected with *H. pylori* (PBS: *n* = 4; *H. pylori*: *n* = 13).** D** KEGG pathway analysis of the GSE27411 dataset. **E** The protein expression level of the inflammatory factors in mice gastric mucosal (PBS: *n* = 2; *H. pylori*: *n* = 3).** F** The RNA expression level of the inflammatory factors in mice gastric mucosal (PBS: *n* = 3; *H. pylori*: *n* = 5). **G** The expression level of inflammatory factors in mice serum (PBS: *n* = 3; *H. pylori*: *n* = 5). **H** Analysis of immune cell infiltration in the GSE27411 database. **I** IF staining of macrophage markers in mice gastric tissue. DAPI was used for nuclear staining (blue), CD68 was used for macrophage marker (green), iNOS was used for M1 marker (red), arrows showed M1 macrophage cells. **J**, **K** The polarization of human monocyte THP-1 co-cultured with *H. pylori*. Abbreviations: H&E, hematoxylin and eosin; IF, Immunofluorescence. The data are presented as the mean ± S.D. after triplicate. Two groups were compared by t-test, multiple groups were compared by one-way analysis of variance (ANOVA). Note: *P < 0.05, **P < 0.01, ***P < 0.001

To determine whether macrophages are aberrantly regulated in *H. pylori*-infected gastric tissues, the slides with H&E stained of gastric tissues were collected, meanwhile *H. pylori*-infected mouse model was also constructed. The *H. pylori*-infected group showed significantly aggravated gastric mucosal inflammation in human and mouse gastric tissues (Fig. 1A-C). Next, the GSE27411 dataset was obtained from the GEO database, which was a microarray dataset of *H. pylori*-infected human gastric mucosal tissues. KEGG pathway enrichment analysis of the GSE27411 dataset showed that pathways were mainly enriched in macrophage-related inflammation signaling pathways, such as the cytokine–cytokine receptor interaction, chemokine signaling pathway, Toll-like receptor signaling pathway, and TNF signaling pathway (Fig. 1D). In mouse gastric mucosal tissues, the expression of ZO-1 decreased, while the expression of inflammatory factors increased in the *H. pylori*-infected group (Fig. 1E, F), as well as in mouse serum, the secretion of inflammatory factors were increased in *H. pylori*-positive group (Fig. 1G). This suggested that *H. pylori* infection caused damage and inflammation of the gastric mucosa. Furthermore, CIBERSORTx immune cell infiltration analysis predicted that the content of M1 macrophages in *H. pylori-*infected tissues increased significantly (Fig. 1H). Immunofluorescence colocalization showed that *H. pylori* stimulated M1 macrophage infiltration in the gastric mucosa (Fig. 1I). Subsequently, THP-1 cells were co-cultured with *H. pylori* to detect macrophage polarization. We found that the expression of TNF-α, IL-1β, and CD86, which are M1 markers, significantly increased (Fig. 1J, K), indicating that *H. pylori* stimulated macrophages to polarize toward to the M1 direction, which is consistent with previous research [25]. Collectively, *H. pylori* infection accelerates gastric inflammation and stimulates M1 macrophage polarization.

#### *H. pylori*-infected macrophage culture medium accelerates disruption of the gastric mucosal barrier

**Fig. 2:**
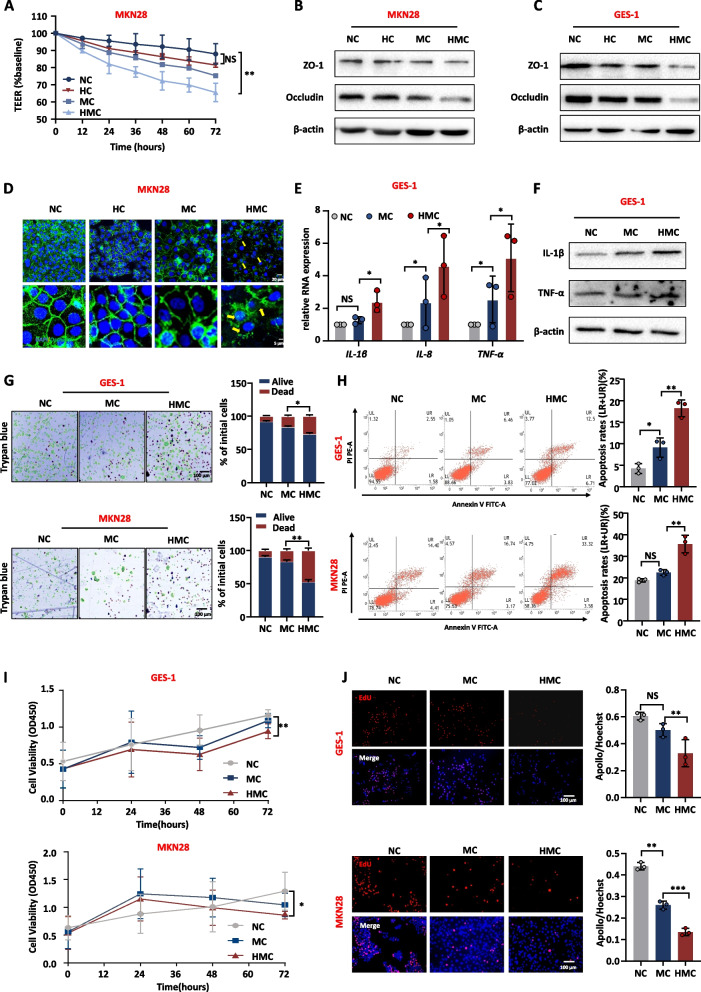
*H. pylori*-infected macrophage medium disrupts the gastric mucosa barrier and promotes inflammation. **A** TEER of MKN28 cells with different culture media. **B**, **C** The protein level of tight junction in MKN28 and GES-1 with different culture media. **D** IF detected the tight junction between MKN28 cells with different culture media. DAPI was used for nuclear staining (blue), Occludin was used for tight junction staining (green), arrows showed the disruption of tight junctions between cells. **E**, **F** The RNA and protein expression level of inflammatory factors in GES-1 with different culture media. **G**, **H** Viability and apoptosis of GES-1 and MKN28 cells with different culture media (**G**: Trypan blue (green circle: live cells; blue: dead cells); **H**: Annexin V- PI apoptosis flow cytometry). **I**, **J** Proliferation of GES-1 and MKN28 cells with different culture media (**I**: CCK8; **J**: EdU). Abbreviations: NC, normal culture medium; MC, macrophage culture medium; HMC, *H. pylori*-infected macrophage culture medium; HC, medium which *H. pylori* was cultured; IF, Immunofluorescence. The data are presented as the mean ± S.D. after triplicate. Two groups were compared by t-test, multiple groups were compared by one-way analysis of variance (ANOVA). Note: *P < 0.05, **P < 0.01, NS, not significant

Substances secreted by macrophages, such as chemokines, play an important role in the progression of inflammation. We want to investigate the potential regulatory effects of substances secreted by *H. pylori-*infected macrophages on inflammation and damage to the gastric mucosa. The supernatant of THP-1 cells infected with *H. pylori* was collected and co-cultured with GES-1 and MKN28 cells. Treatment with *H. pylori*-infected macrophage culture medium (HMC) significantly reduced TEER compared with normal culture medium (NC), macrophage culture medium (MC), and the medium which *H. pylori* was cultured (HC) (Fig. 2A and Supplementary Fig. 1A). Similarly, the expression levels of the tight junction proteins (ZO-1 and Occludin) decreased in HMC group (Fig. 2B and C, Supplementary Fig. 1B, C), and HMC disrupted the tight junctions between the cells (Fig. 2D, Supplementary Fig. 1D). These experiments also suggested that medium which *H. pylori* was cultured does not damage the gastric mucosa. Therefore, in subsequent experiments, the culture medium and macrophage culture medium were used as controls.

Besides, the effects of HMC on inflammation, proliferation, and apoptosis of the gastric epithelium were further evaluated. The expression of inflammatory factors increased in the HMC group (Fig. 2E, F). Furthermore, HMC weakened cell viability and induced apoptosis (Fig. 2G-H). Additionally, HMC treatment significantly inhibited cell proliferation (Fig. 2I, J). Through these experiments, *H. pylori*-infected macrophage culture medium exacerbates the inflammatory response of the gastric mucosa and disrupts the gastric mucosal barrier.

#### *H. pylori* infection promotes macrophage to express CCL3

**Fig. 3:**
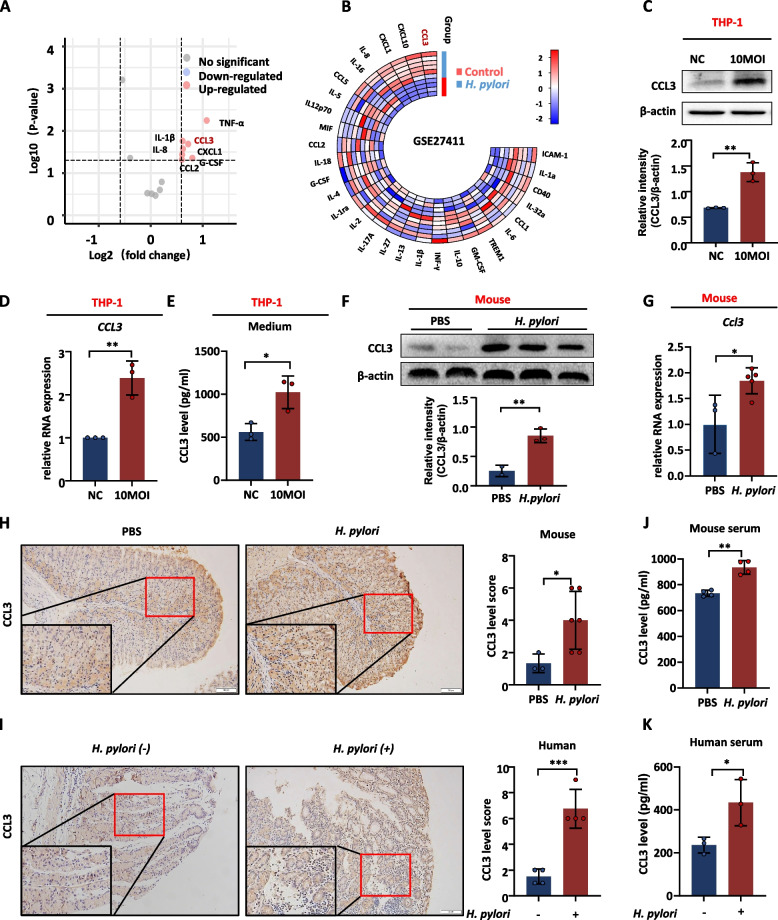
*H. pylori* infection promotes the expression of CCL3. **A** Analysis chemokines in the medium of THP-1 co-cultured with *H. pylori*. **B** Differential analysis of chemokines about the GSE27411 dataset. **C**, **D** The protein and RNA expression level of CCL3 in THP-1 co-cultured with *H. pylori.* **E** The secretion level of CCL3 in the medium of *H. pylori* -infected macrophages. **F** The protein expression level of CCL3 in *H. pylori* infected mice gastric tissue (PBS: *n* = 2; *H. pylori*: *n* = 3). **G** The RNA expression level of CCL3 in *H. pylori* infected mice gastric tissue (PBS: *n* = 3; *H. pylori*: *n* = 5).** H** IHC analysis CCL3 expression in mice (PBS: *n* = 3; *H. pylori*: *n* = 6). **I** IHC analysis CCL3 expression in human (each group: *n* = 4). **J** The expression level of CCL3 in mice serum (each group: *n* = 4). **K** The expression level of CCL3 in human serum (each group: *n* = 3). Abbreviations: IHC, immunohistochemical. The data are presented as the mean ± S.D. after triplicate. Two groups were compared by t-test, multiple groups were compared by one-way analysis of variance (ANOVA). Note: *P < 0.05, **P < 0.01, ***P < 0.001

As shown in Fig. 1D, the pathways were mainly enriched in cytokine–cytokine receptor interactions and chemokine signaling pathways in the *H. pylori*-infected group. Therefore, chemokine antibody microarray assay was performed to evaluate the medium of macrophages infected with *H. pylori*. Results indicated a significant increase in some chemokines (such as TNF-α (fold change = 2.20) and CCL3 (fold change = 1.65)) in the medium of macrophages infected with *H. pylori* (Fig. 3A and Supplementary Fig. 2A). The GSE27411 and GSE5081 datasets also revealed a significant increase in IL-8 and CCL3 in the *H. pylori* infection groups (Fig. 3B and Supplementary Fig. 2B). The significantly upregulated cytokines are likely to be crucial regulators. CCL3, an inflammatory protein secreted mainly by macrophages and rarely expressed in epithelial cells (Supplementary Fig. 3A, B), was significantly upregulated and oversecreted in *H. pylori*-infected THP-1 cells (Fig. 3C-E). In addition, CCL3 expression levels significantly increased in gastric mucosal tissues infected with *H. pylori* in both mouse and human samples (Fig. 3F-I). Similarly, CCL3 secretion in the serum of the *H. pylori*-infected samples was also elevated (Fig. 3J, K). These results demonstrate that *H. pylori* infection stimulates the expression and secretion of CCL3 by macrophages.

#### CCL3 is a critical factor in disrupting the gastric mucosal barrier

**Fig. 4:**
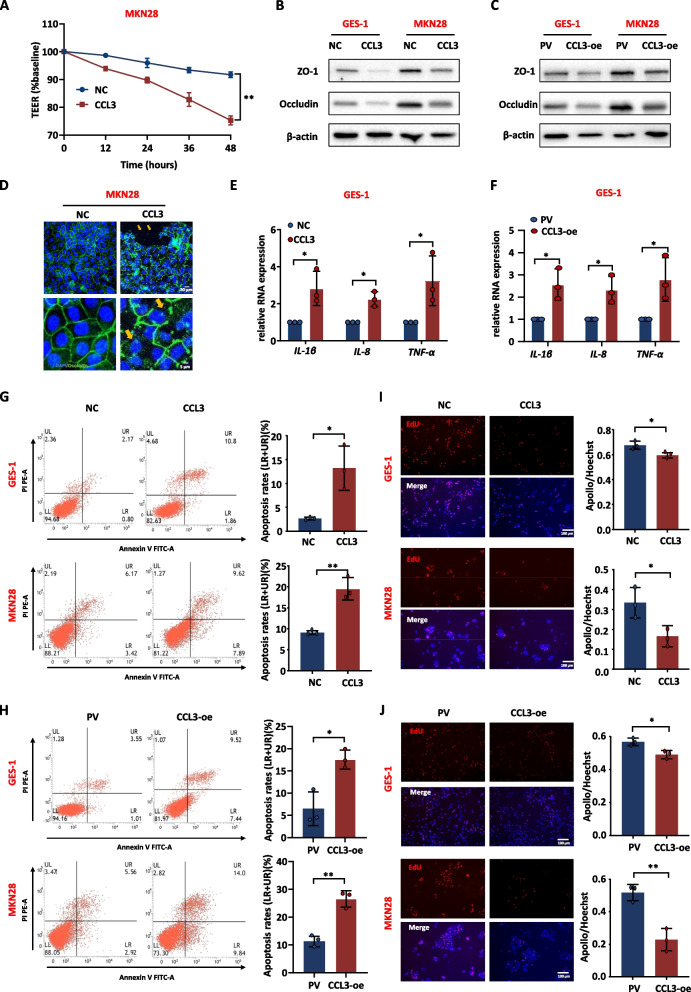
CCL3 disrupts gastric mucosal barrier and promotes inflammation. **A** The effect of recombinant CCL3 protein (160 ng/mL) on the TEER of MKN28 cells. **B**, **C** The protein level of tight junction in MKN28 and GES-1 with recombinant CCL3 or CCL3 overexpressing macrophage medium. **D** IF detected the effect of recombinant CCL3 protein on the tight junction between MKN28 cells. DAPI was used for nuclear staining (blue), Occludin was used for tight junction staining (green), arrows showed the disruption of tight junctions between cells. **E, F** The RNA expression level of inflammatory factors in GES-1 with recombinant CCL3 protein or CCL3 overexpressing macrophage medium. **G**, **H** Apoptosis of GES-1 and MKN28 with recombinant CCL3 protein or CCL3 overexpressing macrophage medium. **I, J** Proliferation of GES-1 and MKN28 with recombinant CCL3 protein or CCL3 overexpressing macrophage medium. Abbreviations: PV, pcDNA vector; IF, Immunofluorescence. The data are presented as the mean ± S.D. after triplicate. Two groups were compared by t-test, multiple groups were compared by one-way analysis of variance (ANOVA). Note: *P < 0.05, **P < 0.01

Next, to verify whether CCL3 is a key regulatory factor in macrophage-induced gastric mucosal damage after *H. pylori* infection, GES-1 and MKN28 cells were stimulated with human CCL3 recombinant protein (160 ng/mL) or medium from THP-1 cells overexpressing CCL3 (Supplementary Fig. 4A-C). TEER decreased and the tight junctions between cells were disrupted when the medium contained excessive levels of CCL3 through the addition of CCL3 recombinant protein or the medium from THP-1 cells overexpressing CCL3 (Fig. 4A-D). At the same time, CCL3 also stimulated the expression of inflammatory factors in GES-1 cells (Fig. 4E, F). CCL3 also promoted GES-1 and MKN28 cells apoptosis (Fig. 4G, H) and inhibited their proliferation (Fig. 4I, J).

**Fig. 5:**
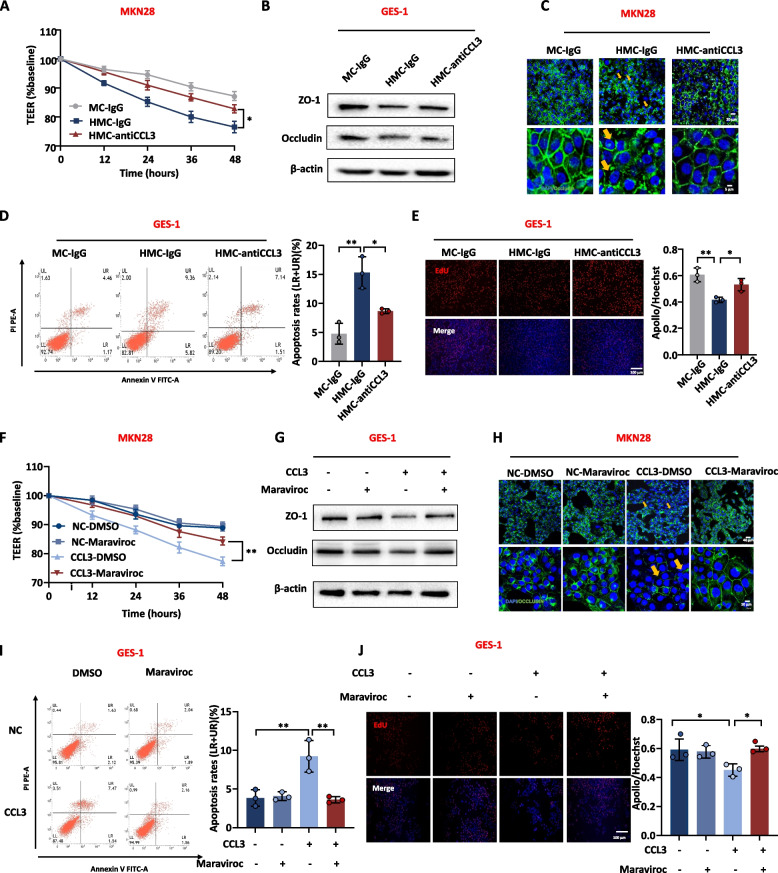
CCL3 neutralizing antibody and CCL3 receptor inhibitor improve mucosal damage and inflammatory response. **A** Effect of CCL3 neutralizing antibody (100 ng/ml) on TEER of MKN28 cells. **B** The protein expression level of the tight junction in GES-1 cells treated with CCL3 neutralizing antibody in conditioned media. **C** IF detected tight junction between MKN28 cells treated with CCL3 neutralizing antibody in conditioned media. DAPI was used for nuclear staining (blue), Occludin was used for tight junction staining (green), arrows showed the disruption of tight junctions between cells. **D**, **E** Apoptosis and proliferation of GES-1 treated with CCL3 neutralizing antibody in conditioned media (**D**: Annexin V- PI apoptosis flow cytometry; **E**: EdU). **F** Effect of CCL3 receptor inhibitor (Maraviroc, 100 nM) on TEER of MKN28 cells. **G** The protein expression level of the tight junction in GES-1 cells treated with CCL3 receptor inhibitor. **H** IF detected the tight junction between MKN28 cells treated with CCL3 receptor inhibitor. **I**, **J** Apoptosis and proliferation of GES-1 treated with CCL3 receptor inhibitor (**I**: Annexin V- PI apoptosis flow cytometry; **J**: EdU). Abbreviations: MC, macrophage culture medium; HMC, *H. pylori*-infected macrophage culture medium; IF, Immunofluorescence. The data are presented as the mean ± S.D. after triplicate Two groups were compared by t-test, multiple groups were compared by one-way analysis of variance (ANOVA). Note: *P < 0.05, **P < 0.01

To further confirm the important regulatory role of CCL3 over secretion by *H. pylori-*infected macrophages, rescue experiments were conducted. CCL3 neutralizing antibody (100 ng/mL) was used to bind the CCL3 protein in HMC. TEER and tight junction proteins recovered after adding CCL3 neutralizing antibody (Fig. 5A-C), meanwhile the CCL3 neutralizing antibody relieved apoptosis (Fig. 5D) and improved cell proliferation (Fig. 5E). GES-1 cells were treated with recombinant CCL3 protein and the CCL3 receptor inhibitor Maraviroc (100 nM) together. Similarly, TEER and the tight junction proteins were increased (Fig. 5F-H), while a reduction in apoptosis (Fig. 5I) and an improvement in proliferation capacity (Fig. 5J) were observed after the addition of the CCL3 receptor inhibitor. Therefore, CCL3 is a critical factor in disrupting the gastric mucosal barrier in vitro.

#### *H. pylori* stimulates macrophages to secrete CCL3 through the JAK1-STAT1 pathway

**Fig. 6:**
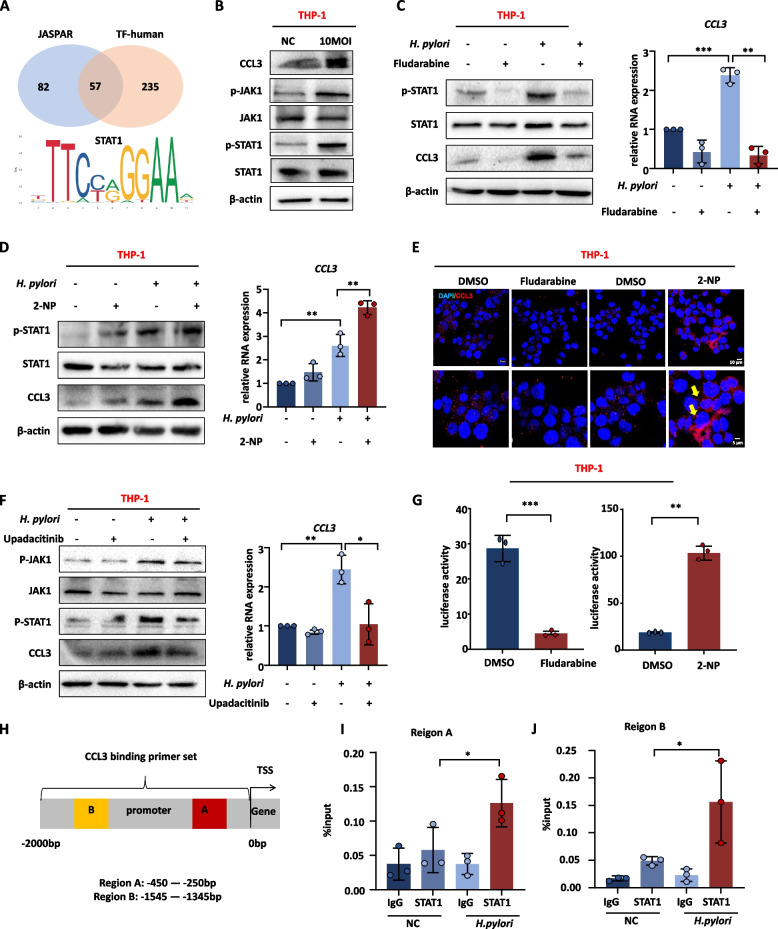
*H. pylori* stimulates macrophages to secrete CCL3 through the JAK1-STAT1 pathway. **A** CCL3 transcription factor prediction (from JASPAR https://jaspar.genereg.net and TF human http://bioinfo.life.hust.edu.cn/HumanTFDB#!/) and the sequence STAT1 binds to the CCL3 promoter (from JASPAR). **B** The protein expression level of CCL3, P-STAT1, P-JAK1 proteins in THP-1 infected with *H. pylori*. **C** The protein and RNA expression level of CCL3 in THP-1 cells treated with STAT1 phosphorylation inhibitor (Fludarabine, 5 μM). **D** The protein and RNA expression level of CCL3 in THP-1 cells treated with STAT1 enhancer (2NP, 45 μM). **E** IF detected the CCL3 expression treated with Fludarabine or 2NP. DAPI was used for nuclear staining (blue), CCL3 was stained in red, arrows showed CCL3 secreted by cells. **F** The protein and RNA expression level of CCL3 in THP-1 cells treated with JAK1 phosphorylation inhibitors (Upadacitinib, 1 μM). **G** THP-1 cells treated with Fludarabine and 2NP were transfected with CCL3 promoter plasmid for dual-luciferase reporter assays. **H**, **I** ChIP verified the binding ability of STAT1 to the CCL3 promoter, and the STAT1 binding sequences were enriched in 250-450 bp and 1345-1545 bp. Abbreviations: IHC, immunohistochemical; IF, Immunofluorescence; ChIP, Chromatin Immunoprecipitation. The data are presented as the mean ± S.D. after triplicate Two groups were compared by t-test, multiple groups were compared by one-way analysis of variance (ANOVA). Note: *P < 0.05, **P < 0.01, ***P < 0.001

The mechanism by which *H. pylori* stimulates macrophages to upregulate CCL3 expression is not clear. The JASPAR and human transcription factor (TF-human) databases were used to predict the transcription factors of CCL3. Based on the intersection of the two databases, STAT1 was identified as the transcription factor with the highest score (Fig. 6A). Additionally, previous studies have revealed that STAT1 is mainly activated by phosphorylated JAK and phosphorylated STATs enter the nucleus to act as transcription factors [26, 27]. The phosphorylation levels of JAK1 and STAT1 increased in THP-1 cells infected with *H. pylori* (Fig. 6B). Phosphorylation levels of STAT1 in the gastric mucosa of *H. pylori* infected mice were elevated (Supplementary Fig. 5). Subsequently, treatment with Fludarabine (5 μM) resulted in a decrease in CCL3 expression, whereas 2-NP (45 μM) treatment led to an increase in CCL3 levels (Fig. 6C-E). In order to investigate that P-JAK1 activates STAT1, the JAK1 phosphorylation inhibitor (Upadacitinib, 1 μM) was added during co-culture. The phosphorylation level of STAT1 was inhibited, as well as CCL3 decreased (Fig. 6F). To further validate whether STAT1 is a transcription factor for CCL3, dual-luciferase reporter assay and ChIP experiments were conducted. Fludarabine reduced CCL3 promoter luciferase activity, whereas 2-NP had the opposite effect (Fig. 6G). ChIP experiments showed that the STAT1 antibody successfully pulled down the CCL3 promoter fragment (Fig. 6H-J). These results indicate that STAT1 is a transcription factor for CCL3, and *H. pylori* stimulates macrophages to secrete CCL3 by activating the JAK1-STAT1 pathway.

#### HMC and chemokine CCL3 disrupt gastric mucosa barrier through P38 phosphorylation

**Fig. 7:**
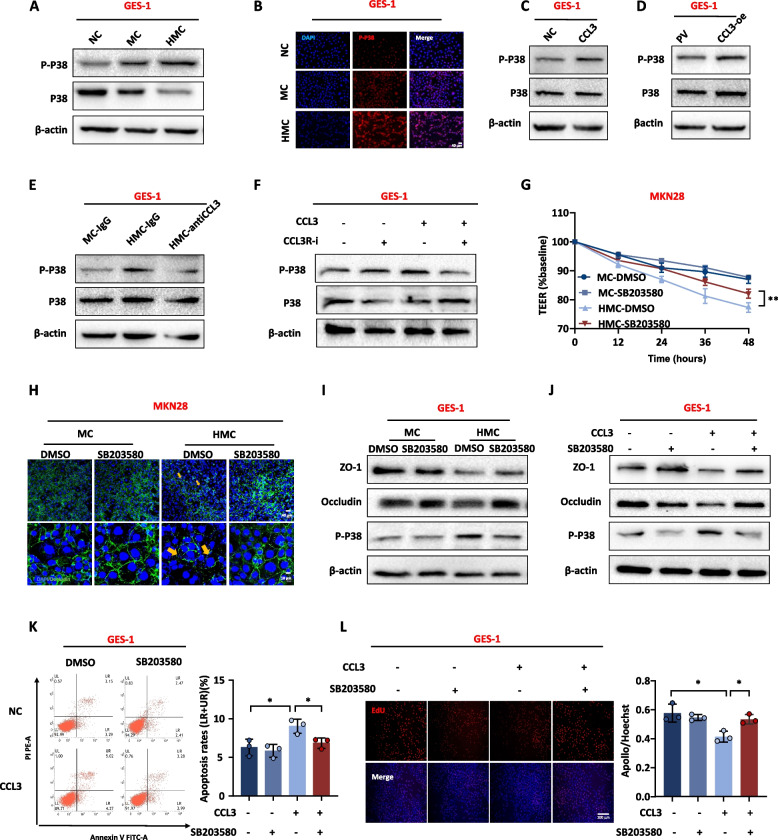
*H. pylori*-infected macrophage culture medium and chemokine CCL3 disrupt gastric mucosal barrier through P38 phosphorylation. **A** The P38 phosphorylation level in GES-1 treated with different conditioned media. **B** IF detected P38 phosphorylation levels in GES-1 treated with different conditioned media. DAPI was used for nuclear staining (blue), P-P38 stained in red. **C**, **D** The P38 phosphorylation level in GES-1 cells with recombinant CCL3 protein (160 ng/mL) and CCL3 overexpressing macrophage medium. **E**, **F** The P38 phosphorylation level in GES-1 cells treated with CCL3 neutralizing antibody (100 ng/mL) and CCL3 receptor inhibitor (Maraviroc, 100 nM). **G** Effects of P38 phosphorylation inhibitor (SB203580, 10 μM) on TEER of MKN28 cells. **H** IF detected the effect of P38 phosphorylation inhibitor on tight junctions between MKN28 cells. DAPI was used for nuclear staining (blue), Occludin was used for tight junction staining (green), arrows showed the disruption of tight junctions between cells. **I**,** J** The protein expression level of tight junction in GES-1 cells treated with P38 phosphorylation inhibitor. **K**, **L** Apoptosis and proliferation of GES-1 treated with P38 phosphorylation inhibitor (**K**: Annexin V- PI apoptosis flow cytometry; **L**: EdU). Abbreviations: NC, normal culture medium; MC, macrophage culture medium; HMC, *H. pylori*-infected macrophage culture medium; PV, pcDNA vector; IF, Immunofluorescence. The data are presented as the mean ± S.D. after triplicate. Two groups were compared by t-test, multiple groups were compared by one-way analysis of variance (ANOVA). Note: *P < 0.05, **P < 0.01

Next, we explored the conventional inflammatory pathways and found that HMC and CCL3 induced P38 phosphorylation rather than activated the NF-κB signaling pathway (Fig. 7A-D and Supplementary Fig. 6A-C). Moreover, a reduction in phosphorylated P38 levels was observed after the addition of CCL3 neutralizing antibody or CCL3 receptor inhibitor (Fig. 7E, F). To further clarify whether these effects were mediated through the P38 phosphorylation, the P38 phosphorylation inhibitor (SB203580, 10 μM) was used. An improvement in TEER (Fig. 7G) and tight junction disruption (Fig. 7H–J) were observed. The inhibition of P38 phosphorylation also rescued apoptosis and cell proliferation (Fig. 7K, L). These results suggest that HMC and CCL3 exert their effects through P38 phosphorylation.

#### CCL3 recombinant protein aggravates gastric mucosal inflammation and promotes P38 phosphorylation in vivo

**Fig. 8:**
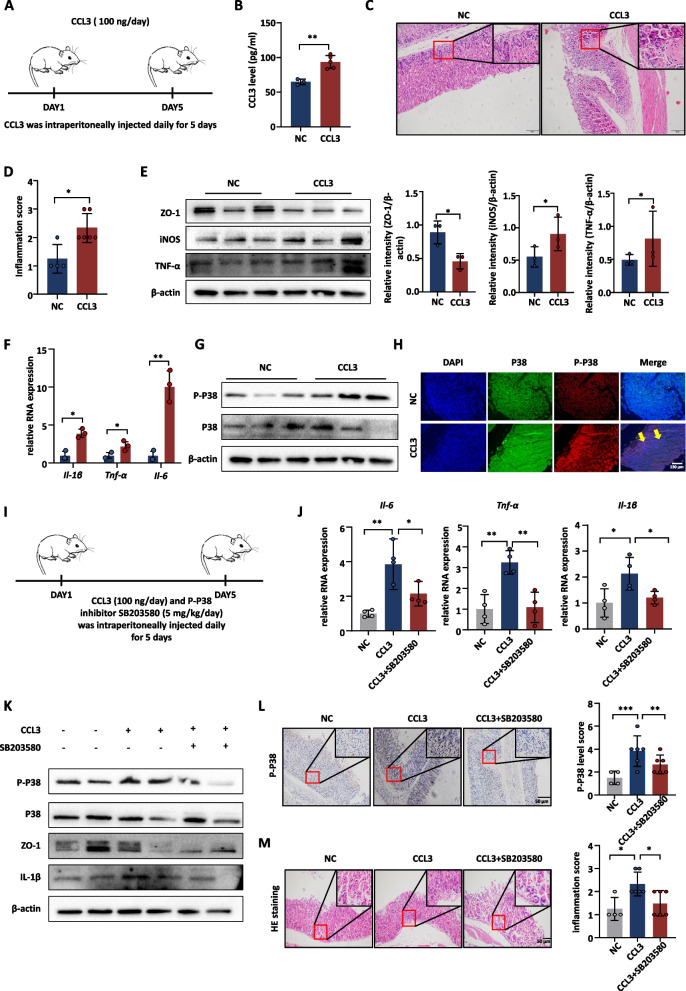
CCL3 recombinant protein aggravates gastric mucosal inflammation and promotes P38 phosphorylation in vivo. **A** The model of murine CCL3 recombinant protein (100 ng/day) intraperitoneal injection. **B** The secretion level of CCL3 in mice serum (NC: *n* = 3; CCL3: *n* = 5). **C**, **D** H&E staining and inflammation score of mice gastric mucosa (NC: *n* = 4; CCL3: *n* = 6). **E** The protein expression level of the inflammatory factors in mice gastric tissue (each group: *n* = 3). **F** The RNA expression level of the inflammatory factors in mice gastric tissue (NC: *n* = 2; CCL3: *n* = 3). **G** The protein expression level of P-P38 in mice gastric tissue (each group: *n* = 3). **H** IF detected P38 phosphorylation levels in mice gastric tissue. DAPI was used for nuclear staining (blue), P38 was stained in green, P-P38 was stained in red, arrows showed P-P38-expressing cells. **I** The model of CCL3 recombinant protein and P38 phosphorylation inhibitor (SB203580, 5 mg/kg/day) intraperitoneal injection. **J** The RNA expression level of inflammatory factors in mice gastric tissue (each group: *n* = 4). **K** The protein expression level of inflammatory factors in mice gastric tissue (each group: *n* = 2). **L** IHC detected the P38 phosphorylation levels in mice gastric tissue (NC: *n* = 4; CCL3: *n* = 6; CCL3 + SB203580: *n* = 6). **M, H**&**E** staining of mice gastric mucosa (NC: *n* = 4; CCL3: *n* = 6; CCL3 + SB203580: *n* = 6). Abbreviations: H&E, hematoxylin and eosin; IHC, immunohistochemical; IF, Immunofluorescence. The data are presented as the mean ± S.D. after triplicate. Two groups were compared by t-test, multiple groups were compared by one-way analysis of variance (ANOVA). Note: *P < 0.05, **P < 0.01, ***P < 0.001

In order to assess the potential damage caused by CCL3 to the gastric mucosal barrier in vivo, mice were intraperitoneally injected with murine CCL3 recombinant protein (100 ng/day) (Fig. 8A). The level of CCL3 in serum increased following the injection of CCL3 recombinant protein (Fig. 8B), leading to inflammation in gastric mucosa (Fig. 8C, D). The results showed that the expression of ZO-1 decreased, while inflammatory factors increased in the CCL3-injected group (Fig. 8E, F). We also found that P38 phosphorylation levels increased in the gastric mucosa (Fig. 8G, H and Supplementary Fig. 7). Additionally, the mice were injected with P38 phosphorylation inhibitor (SB203580, 5 mg/kg/day) to confirm that CCL3 exerted functions via the P38 phosphorylation (Fig. 8I). SB203580 improved the inflammatory response of the mice gastric mucosa through inhibiting P38 phosphorylation (Fig. 8J-M). Therefore, CCL3 causes inflammation in the gastric mucosa, mainly through P38 phosphorylation in vivo.

## Discussion

**Fig. 9:**
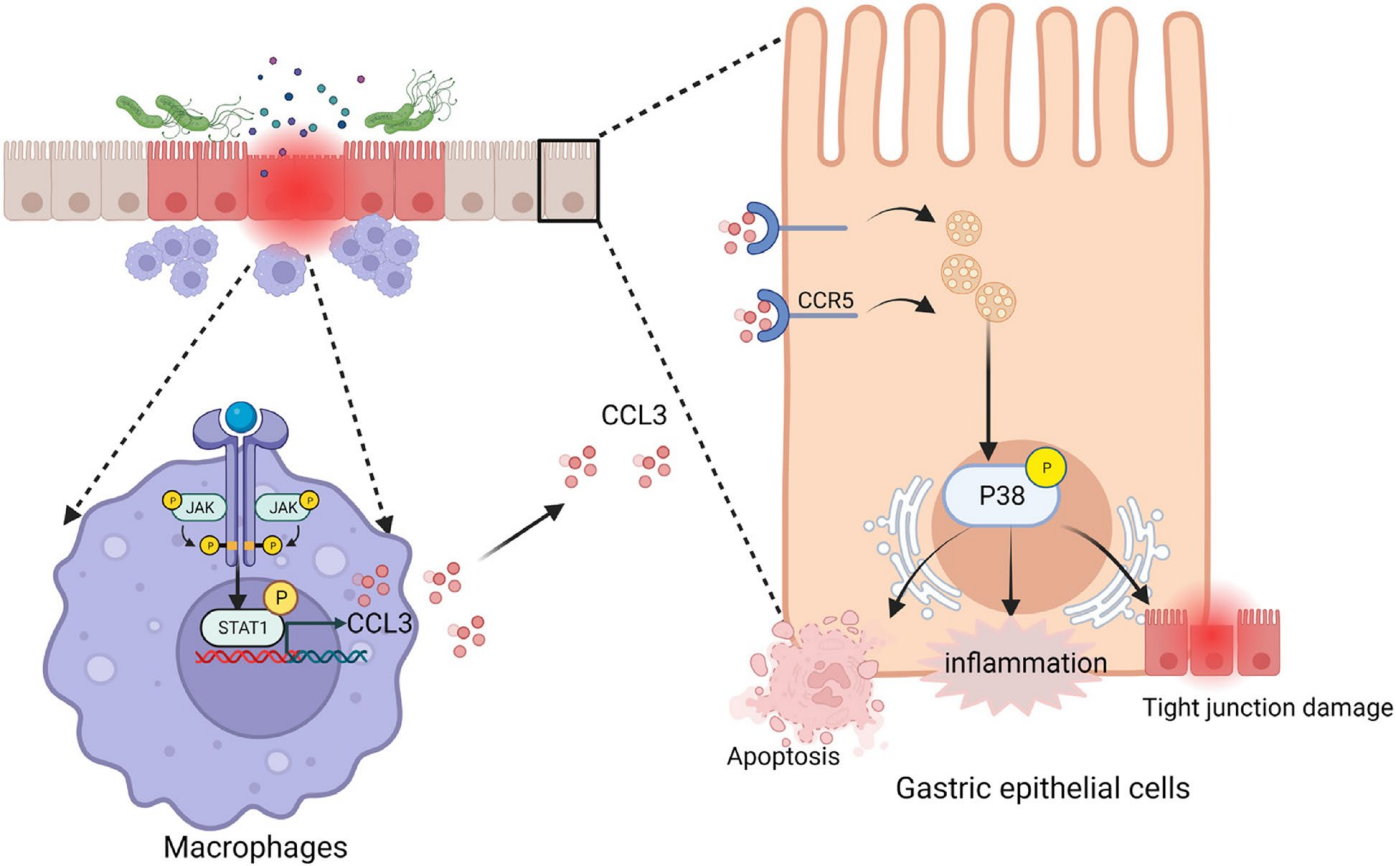
Schematic diagram of the mechanism of gastric mucosal damage caused by CCL3 which is secreted by *H. pylori* infected macrophages. *H. pylori* induces the expression of the chemokine CCL3 by macrophages via the JAK1-STAT1 pathway, and CCL3 disrupts the gastric mucosal barrier via P38 phosphorylation

In this study we demonstrate that *H. pylori* induces the secretion of the chemokine CCL3 by macrophages via the JAK1-STAT1 pathway. Additionally, CCL3 disrupts the gastric mucosal barrier via P38 phosphorylation (Fig. 9).

*H. pylori* was discovered in the 1980s by Marshall and Warren, and it infects 50% of the world population [1, 28]. Persistent infection with *H. pylori* is closely associated with various upper gastrointestinal diseases, including gastritis, peptic ulcers, and gastric adenocarcinoma [29]. Colonization by *H. pylori* triggers the release of inflammatory mediators, which attract immune cells to the site of infection. Macrophages are one of the main immune cells involved in *H. pylori*-induced gastric inflammation. Macrophages polarize into two distinct phenotypes (M1 and M2). M1 macrophages display antimicrobial and antitumor properties, whereas M2 macrophages exhibit anti-inflammatory capabilities [30]. Our findings indicated that *H. pylori* infection promoted the polarization of macrophages to the M1 direction, leading to the exacerbation of the gastric mucosal inflammatory response. However, the levels of M2 markers also increased after *H. pylori* infection. The polarization direction of macrophages during *H. pylori* infection exhibits a mixed phenotype in gastric biopsy specimens [31]. Furthermore, the burden of *H. pylori* affects macrophage polarization: a low MOI (10, 50, 100) promotes both M1 and M2 phenotypes, whereas a high MOI (200) inhibits the M2 phenotype in vitro experiments [32]. In addition, the human body initiates mechanisms that suppress M1 polarization during *H. pylori* infection to relieve inflammation. Infection with *H. pylori* leads to an upregulation in the expression of ARG2, resulting in decreased inflammatory cytokine expression and the inhibition of M1 macrophage activation [33]. We also considered whether the polarization of macrophages stimulated by *H. pylori* was related to the duration of bacterial infection, which can be used as a direction for future research.

In this study, macrophages were stimulated to secrete a variety of chemokines by *H. pylori*. Chemokines, which are small peptides with molecular weights ranging from 8–14 kDa, play a crucial role in attracting different types of immune cells to the site of inflammation [34]. *H. pylori* stimulates macrophages to secrete cytokines such as IL-6, TNF-α, IL-8, and IL-1β [35, 36]. Our research reveals that *H. pylori* induces the secretion of the chemokine CCL3 by macrophages, which is implicated in several inflammation-related diseases, including asthma, wound healing, arthritis, multiple sclerosis and pneumonia [19]. CCL3 exacerbates intestinal inflammation and impairs the integrity of the intestinal mucosal barrier [37]. Our study identifies an association between CCL3 and *H. pylori*-related gastritis, which leads to the damage of the gastric mucosa. CCL3 exerts a significant influence on inflammatory diseases through diverse mechanisms. CCL3 promotes apoptosis by activating the ERK1/2 and NF-κB pathways in necrotizing enterocolitis (NEC) [37]. Additionally, CCL3 induces inflammatory responses in acute pancreatitis (AP) by activating the JNK/ p38 MAPK signaling pathway [38]. In our study, CCL3 damages the gastric mucosa through the phosphorylation of P38. The P38 signaling pathway plays a significant role in the development of gastric mucosal inflammation induced by *H. pylori*. CagA + strains of *H. pylori* are more effective in activating the P38 pathway [39], whereas the VacA toxin of *H. pylori* induces cell apoptosis through P38 pathway [40]. NF-κB is a classical inflammatory pathway, but in our study, there are no significant alterations in the NF-κB pathway after the treatment of CCL3 or HMC. *H. pylori* imports CagA into epithelial cells, subsequently activates NF-κB and promotes the secretion of inflammatory factors [41]. Therefore, we think that the NF-κB pathway may be related to the release of inflammatory factors rather than to mucosal damage.

Based on the above findings, targeted inhibition of CCL3 and P38 phosphorylation may improve gastric mucosal inflammation and injury. Currently, no specific inhibitors are available for CCL3; therefore, targeted therapy mainly focuses on blocking its receptors (CCR1 or CCR5). CCL3 receptors targeted therapy is currently undergoing clinical trials to treat inflammation-related diseases. The CCR1 inhibitor CP-481715 can improve inflammation by reducing the number of monocytes in synovial effusions in patients with rheumatoid arthritis [42]. Similarly, the CCR1 inhibitor BX471, evaluated as a drug candidate for multiple sclerosis, can alleviate immune infiltration [43]. The CCR5 inhibitor Maraviroc has been approved for the treatment of HIV [44]. Cenicriviroc, an inhibitor of CCR2/CCR5, demonstrated good efficacy in a phase II clinical trial for COVID-19 [45]. CCL3 plays a significant role in these diseases, but CCR1 and CCR5 are not specific receptors for CCL3. Therefore, it is imperative to consider the possibility that symptom improvement may be attributed to the inhibition of other chemokines. Consequently, the development of specific CCL3 inhibitors is necessary. The P38 pathway is crucial for the initiation and progression of inflammation. Specific inhibitors of P38 are also currently undergoing clinical trials, and extensive research have been conducted on their application in chronic obstructive pulmonary disease (COPD) [46]. Short-term administration of acumapimod can enhance lung function and alleviate inflammation [47]. Furthermore, clinical trials have investigated the effectiveness of BIRB796 in patients with Crohn's disease [48], as well as pamapimod, VX745, and VX702 in rheumatoid arthritis [49–51].

Our study also has some limitations. First, we verified that CCL3 induced mucosal inflammation and injury by intraperitoneally injecting mice with the recombinant protein. However, in order to make the experiment more complete, it is necessary to supplement the relevant experiments of *H. pylori* infected CCL3 knockout mice or macrophage-clearing mice in the future. Secondly, the mechanisms of *H. pylori* stimulating macrophages to secrete CCL3 and CCL3 damage to the gastric mucosa were simply proven. Finally, in the future, compounds or drugs that target related pathways can be screened, and then it can be determined whether they can improve gastric mucosal injury.

## Conclusions

In conclusion, we have revealed that *H. pylori* stimulates macrophages to secrete CCL3 by activating the JAK1-STAT1 pathway. The chemokine CCL3 serves as a novel mucosal damage factor and exerts its effects on the gastric mucosa through P38 phosphorylation. Therefore, inhibiting the secretion of CCL3 or blocking P38 phosphorylation may become new targets for improving gastric mucosal damage, providing new insights into the pathogenic mechanism and treatment of *H. pylori-*related gastritis.

### Supplementary Information

**Additional file 1.**

**Additional file 2:** **Supplementary fig. 1** *H. pylori*-infected macrophage medium disrupts gastric mucosal barrier. A TEER of MKN28 cells with different culture media. B-C The protein level of tight junction in MKN28 and GES-1 with different culture media. D IF detected the tight junction between MKN28 cells with different culture media. DAPI was used for nuclear staining (blue), Occludin was used for tight junction staining (green), arrows showed the disruption of tight junctions between cells. Abbreviations: NC, normal culture medium; MC, macrophage culture medium; HMC, *H. pylori*-infected macrophage culture medium; IF, Immunofluorescence. The data are presented as the mean±S.D. after triplicate. Two groups were compared by t-test, multiple groups were compared by one-way analysis of variance (ANOVA). Note: **P < 0.01. **Supplementary fig. 2** *H. pylori* stimulates macrophages to secrete chemokines. A Analysis chemokines in the medium of THP-1 co-cultured with *H. pylori* by antibody chip. B Differential analysis of chemokines using the GSE5081 dataset. **Supplementary fig. 3** Chemokine CCL3 distribution in cell types and gastric tissue. A-B Analysis the distribution of the chemokine CCL3 in cell types and gastric tissue using THE HUMAN PROTEIN ATLAS database (https://www.proteinatlas.org/). **Supplementary fig. 4** The verification of CCL3 overexpression efficiency. A-B The RNA and protein expression level of CCL3 after transfected CCL3 overexpressed-plasmid in THP-1 cells. C The level of CCL3 in the medium of CCL3 overexpressing macrophages. Abbreviations: PV, pcDNA vector. The data are presented as the mean±S.D. after triplicate. Two groups were compared by t-test. Note: *P < 0.05. **Supplementary fig. 5** STAT1 phosphorylation level in mice gastric mucosa. A The protein expression level of P-STAT1 in the gastric mucosa of mice infected or uninfected with *H. pylori* (PBS: *n*=2; H. pylori: *n*=3). **Supplementary fig. 6** Exploration of pathways for gastric mucosal barrier damage caused by conditioned media and CCL3. A The phosphorylation level of P65 in GES-1 and MKN28 cells treated with different culture media. B The phosphorylation level of P65 in GES-1 treated with recombinant CCL3 protein (160 ng/ml). C The phosphorylation level of P65 in GES-1 and MKN28 cells treated with CCL3 overexpressing macrophage medium. Abbreviations: PV, pcDNA vector. **Supplementary fig. 7** The phosphorylation level of P38 in mice injected with CCL3 recombinant protein. A IHC detected the phosphorylation level of P38 in mice gastric mucosal (NC: *n*=4; CCL3: *n*=6). Abbreviations: IHC, immunohistochemical. Two groups were compared by t-test. Note: **P < 0.01.

**Additional file 3.**

## Data Availability

Data will be made available on request.

## Abbreviations

*H. Pylori*: *Helicobacter* *p**ylori*
IL: Interleukins
TNF: Tumor necrosis factor
NO: Nitric oxide
MIP-1α: Macrophage inflammatory protein 1α
LPS: Lipopolysaccharide
FBS: Fetal bovine serum
PMA: Phorbol 12-myristate-13-acetate
SS1: Sydney Strain 1
PBS: Phosphate-buffered saline
MOI: Multiplicity of infection
RT-qPCR: Real-Time quantitative PCR
WB: Western Blot
PVDF: Polyvinylidene fluoride
CCK8: Cell Counting Kit-8
EdU: 5-Ethynyl-2-deoxyuridine
FACS: Fluorescence-activated cell sorting
IF: Immunofluorescence trans
TEER: Epithelial electrical resistance
BSA: Bovine serum albumin
TSS: Transcription start site
ChIP: Chromatin Immunoprecipitation
ELISA: Enzyme-linked immunosorbent assay
H&E: Hematoxylin and eosin
IHC: Immunohistochemical
DAB: Diaminobenzidine
GEO: Gene Expression Omnibus
KEGG: Kyoto Encyclopedia of Genes and Genomes
HMC: *H. pylori*-Infected macrophage culture medium
NC: Normal culture medium
MC: Macrophage culture medium
HC: Medium which *H. pylori* was cultured
NEC: Necrotizing enterocolitis
AP: Acute pancreatitis
COPD: Chronic obstructive pulmonary disease
