# Supplementary material for: Helicobacter pylori disrupts gastric mucosal homeostasis by stimulating macrophages to secrete CCL3
Source: Cell Commun Signal. 2024 May 10;22:263. doi: 10.1186/s12964-024-01627-5 (PMC11084090; doi:10.1186/s12964-024-01627-5)
Supplement: Supplementary file 1 — Additional file 1. [file 12964_2024_1627_MOESM1_ESM.pdf]

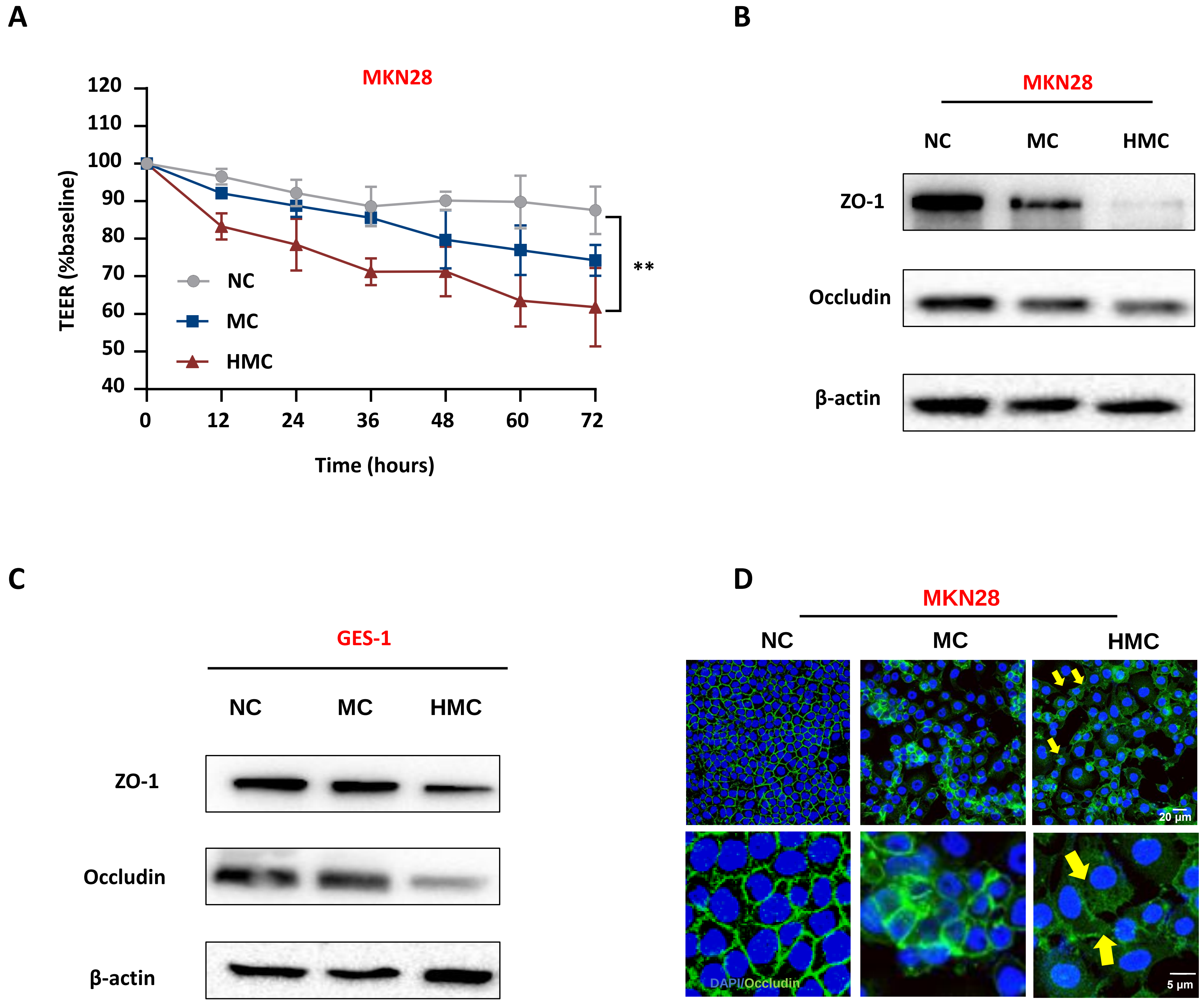

Supplementary figure 1

A

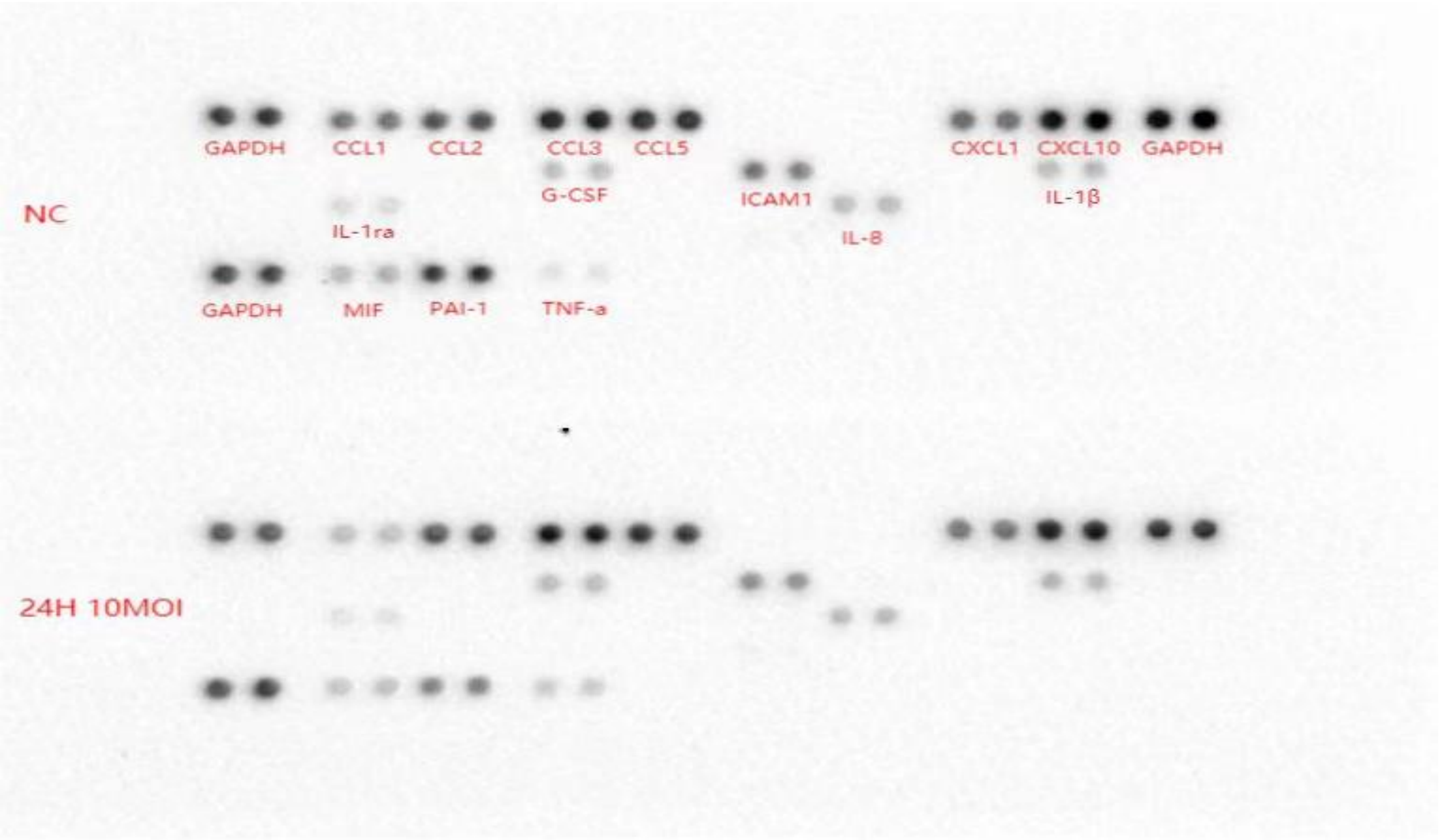

B

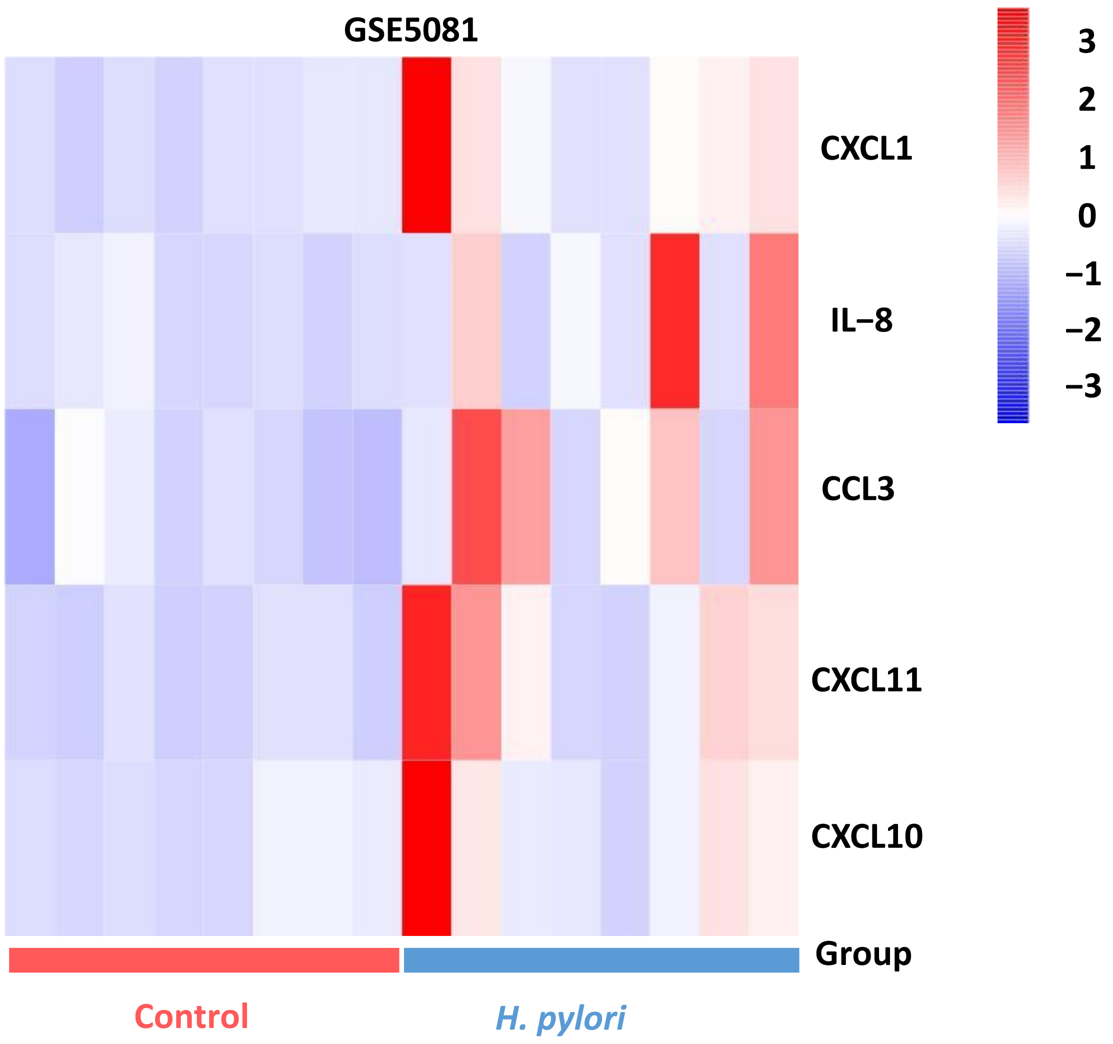

Supplementary figure 2

A

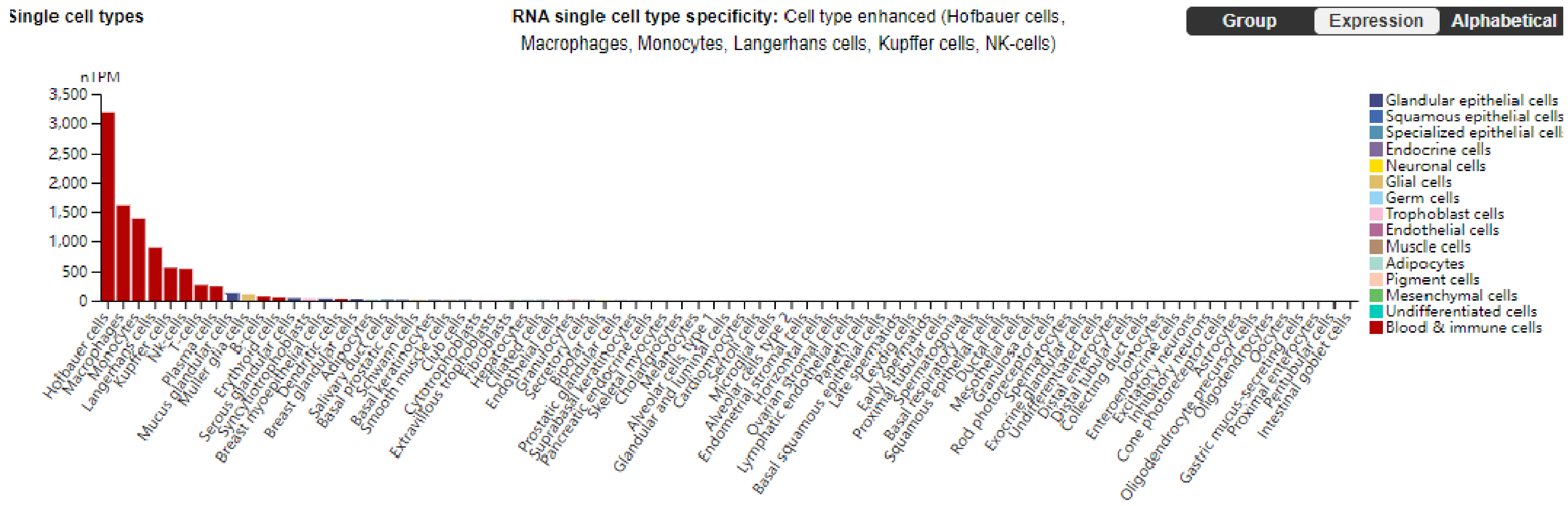

B

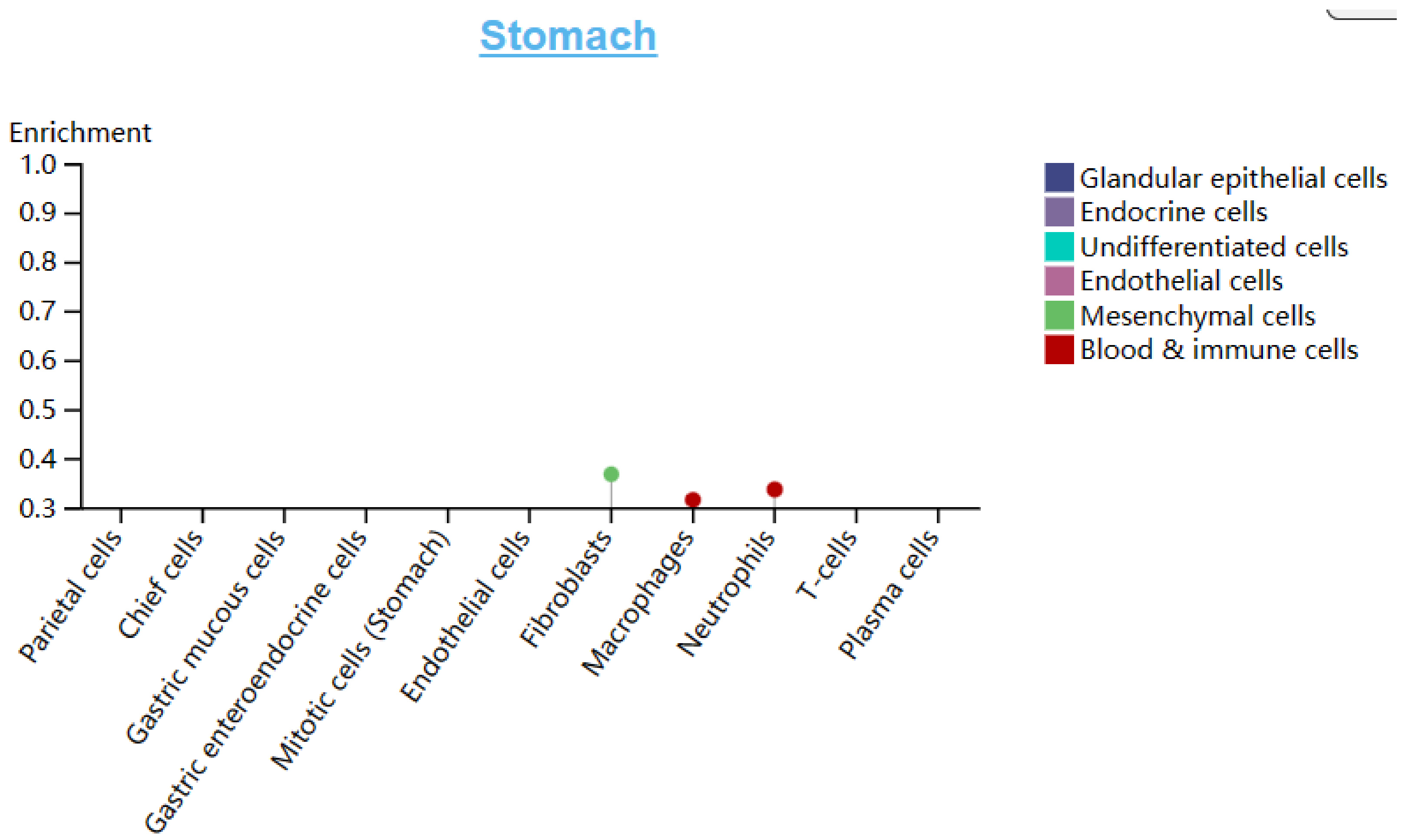

Supplementary figure 3

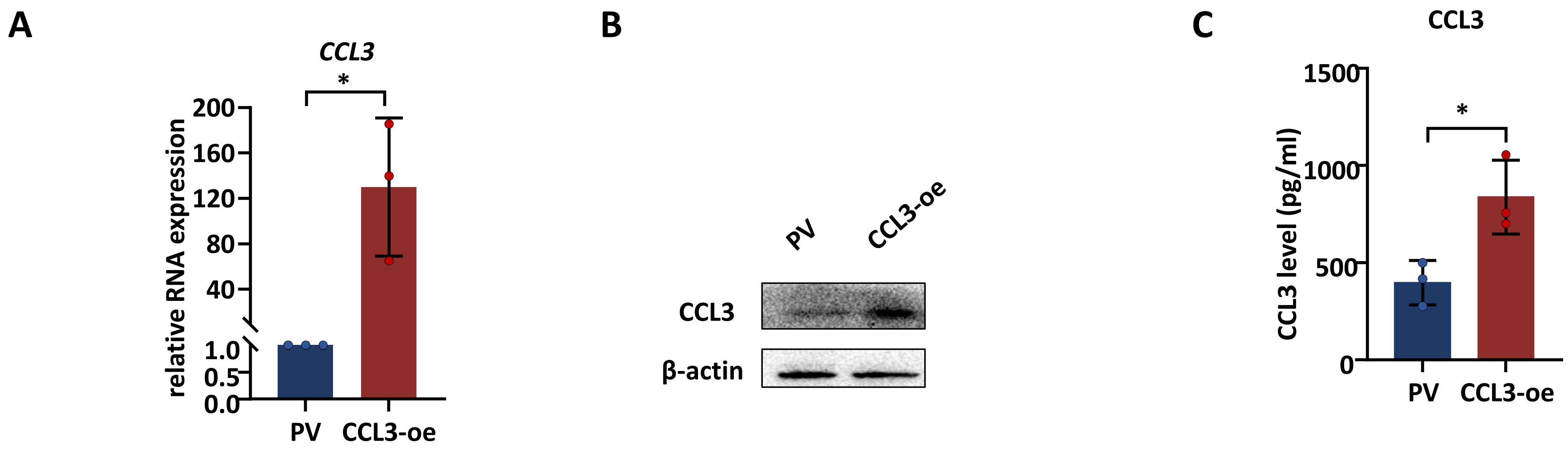

Supplementary figure 4

A

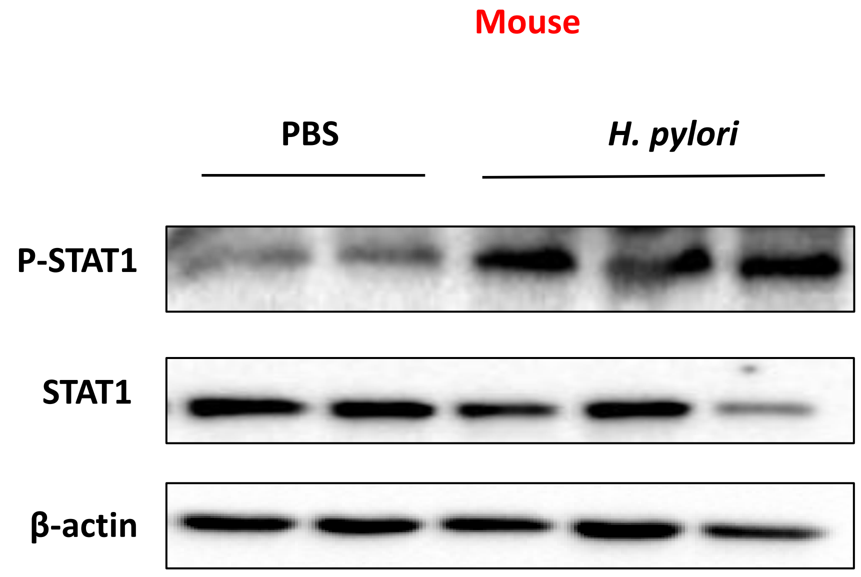

Supplementary figure 5

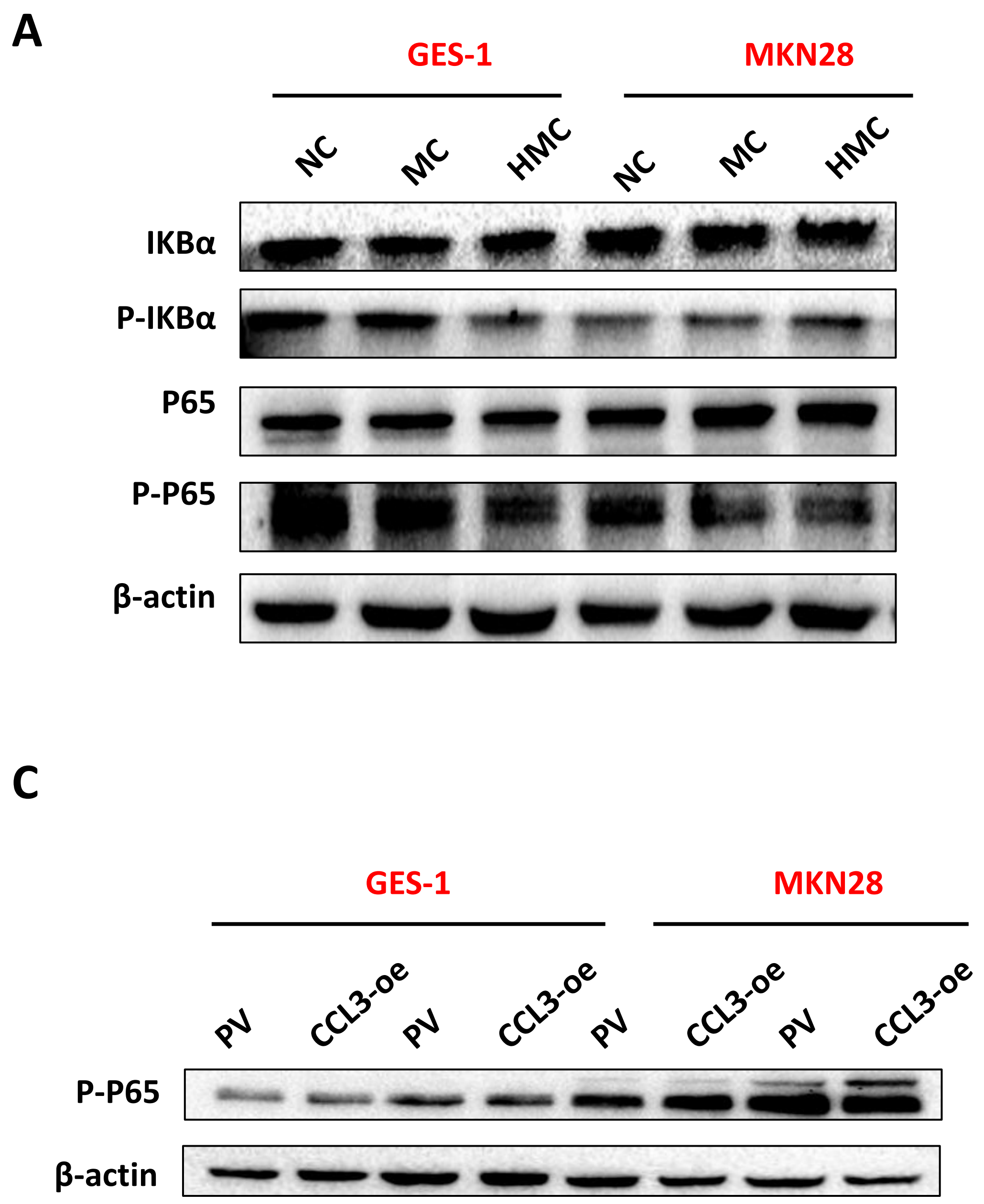

Supplementary figure 6

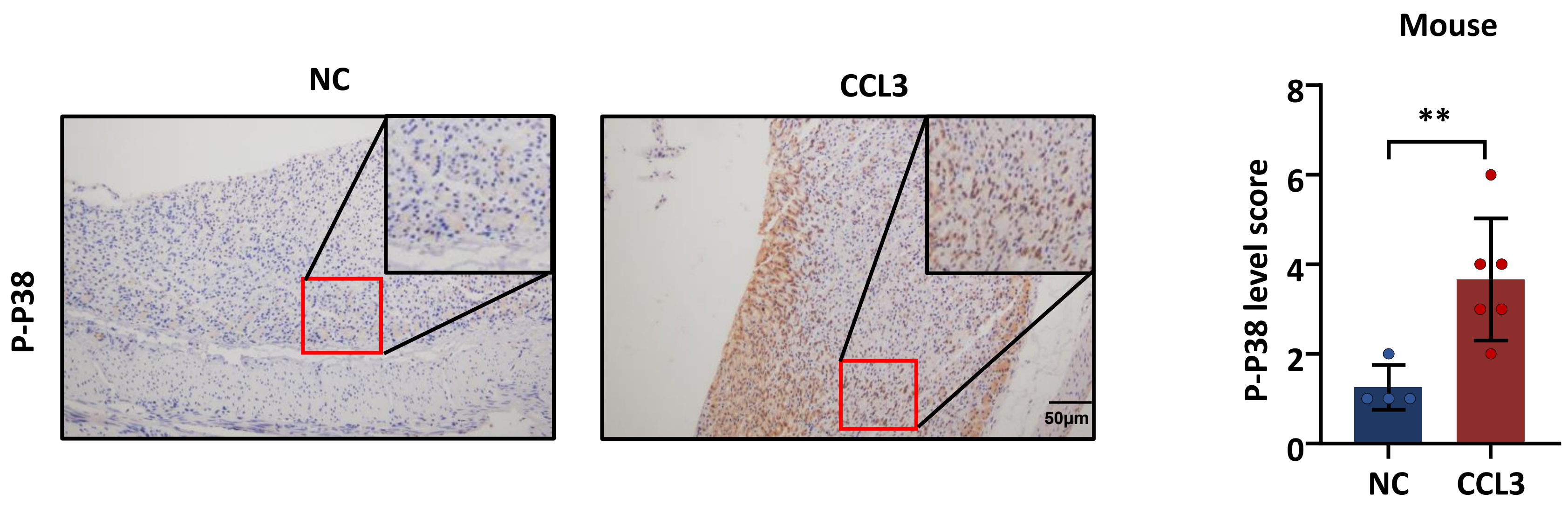

Supplementary figure 7
