# Supplementary material for: Helicobacter pylori disrupts gastric mucosal homeostasis by stimulating macrophages to secrete CCL3
Source: Cell Commun Signal. 2024 May 10;22:263. doi: 10.1186/s12964-024-01627-5 (PMC11084090; doi:10.1186/s12964-024-01627-5)
Supplement: Supplementary file 2 — Additional file 2: Supplementary fig. 1 H. pylori-infected macrophage medium disrupts gastric mucosal barrier. A TEER of MKN28 cells with different culture media. B-C The protein level of tight junction in MKN28 and GES-1 with different culture media. D IF detected the tight junction between MKN28 cells with different culture media. DAPI was used for nuclear staining (blue), Occludin was used for tight junction staining (green), arrows showed the disruption of tight junctions between cells. Abbreviations: NC, normal culture medium; MC, macrophage culture medium; HMC, H. pylori-infected macrophage culture medium; IF, Immunofluorescence. The data are presented as the mean±S.D. after triplicate. Two groups were compared by t-test, multiple groups were compared by one-way analysis of variance (ANOVA). Note: **P < 0.01. Supplementary fig. 2 H. pylori stimulates macrophages to secrete chemokines. A Analysis chemokines in the medium of THP-1 co-cultured with H. pylori by antibody chip. B Differential analysis of chemokines using the GSE5081 dataset. Supplementary fig. 3 Chemokine CCL3 distribution in cell types and gastric tissue. A-B Analysis the distribution of the chemokine CCL3 in cell types and gastric tissue using THE HUMAN PROTEIN ATLAS database (https://www.proteinatlas.org/). Supplementary fig. 4 The verification of CCL3 overexpression efficiency. A-B The RNA and protein expression level of CCL3 after transfected CCL3 overexpressed-plasmid in THP-1 cells. C The level of CCL3 in the medium of CCL3 overexpressing macrophages. Abbreviations: PV, pcDNA vector. The data are presented as the mean±S.D. after triplicate. Two groups were compared by t-test. Note: *P < 0.05. Supplementary fig. 5 STAT1 phosphorylation level in mice gastric mucosa. A The protein expression level of P-STAT1 in the gastric mucosa of mice infected or uninfected with H. pylori (PBS: n=2; H. pylori: n=3). Supplementary fig. 6 Exploration of pathways for gastric mucosal barrier damage caused by con [file 12964_2024_1627_MOESM2_ESM.pdf]

Supplementary Table 1 List of primer sequences used for RT-qPCR and ChIP

Primer name	Sequence 5'-3'
H-*GAPDH*-F	GGAGCGAGATCCCTCCAAAAT
H-*GAPDH*-R	GGCTGTTGTCATACTTCTCATGG
m-*Gapdh*-F	CATCAAGAAGGTGGTGAAGC
m-*Gapdh*-R	CCTGTTGCTGTAGCCGTATT
H-*ZO-1*-F	ACCAGTAAGTCGTCCTGATCC
H-*ZO-1*-R	TCGGCCAAATCTTCTCACTCC
m-*Zo-1*-F	GCCGCTAAGAGCACAGCAA
m-*Zo-1*-R	GCCCTCCTTTTAACACATCAGA
m-*Il-6*-F	CTGCAAGAGACTTCCATCCAG
m-*Il-6*-R	AGTGGTATAGACAGGTCTGTTGG
H-*TNF-α*-F	TCTTCTCGAACCCCGAGTGA
H-*TNF-α*-R	CCTCTGATGGCACCACCAG
m-*Tnf-α*-F	GGTGCCTATGTCTCAGCCTC
m-*Tnf-α*-R	GCTCCTCCACTTGGTGGTTT
H-*CD86*-F	CTGCTCATCTATACACGGTTACC
H-*CD86*-R	GGAAACGTCGTACAGTTCTGTG
H-*IL-1β*-F	GGACAAGCTGAGGAAGATGC
H-*IL-1β*-R	TCGTTATCCCATGTGTCGAA
m-*Il-1β*-F	GAAATGCCACCTTTTGACAGTG
m-*Il-1β*-R	TGGATGCTCTCATCAGGACAG
m-*Ccl3*-F	TGTACCATGACACTCTGCAAC
m-*Ccl3*-R	CAACGATGAATTGGCGTGGAA
H-*CCL3*-F	AGTTCTCTGCATCACTTGCTG
H-*CCL3*-R	CGGCTTCGCTTGGTTAGGAA
H-*IL-8*-F	TTTTGCCAAGGAGTGCTAAAGA
H-*IL-8*-R	AACCCTCTGCACCCAGTTTTC
CCL3(1345-1545bp)-F	GGCACCTGACATATTGACACC
CCL3(1345-1545bp)-R	GTGACTAGGGCGCTGTGTTA
CCL3(250-450bp)-F	GGTCACTTCTTCCCCTCGTG
CCL3(250-450bp)-R	CTGAGATGCCCCTGATCTCG

Supplementary Table 2 List of antibodies used for western blot

Antibody	Company	Lot	Dilution ratio(WB)
CCL3	SANTA	SC-166942	1:500
ZO-1	proteintech	21773-1	1:5000
OCCLUDIN	proteintech	27260-1	1:5000
β-ACTIN	proteintech	66009-1	1:20000
TNF-α	proteintech	60291-1	1:2000
IL-1β	proteintech	16806-1	1:5000
iNOS	Abcame	Ab178945	1:1000
CD86	proteintech	26903-1	1:2000
P-P38	SANTA	Sc7973	1:1000
P38	proteintech	14064-1	1:1000
P-STAT1	SANTA	Sc-8394	1:1000
STAT1	CST	9172	1:1000
P-JAK1	Bioss	Bs-3238	1:1000
JAK1	proteintech	66466-1	1:4000

Supplementary Table 3 List of basic information of patients who are used for H&E and serum ELISA.

Number	Gender	Age	Endoscopic diagnosis	*H.pylori* pathology report	^{13}C text
1	female	68	Erosive gastritis	positive	Not done
2	female	44	Erosive gastritis	positive	Not done
3	male	46	Erosive gastritis	positive	Not done
4	female	66	Erosive gastritis	positive	Not done
5	female	68	Erosive gastritis	positive	positive
6	male	68	Erosive gastritis	positive	positive
7	male	48	Erosive gastritis	positive	positive
8	male	65	Erosive gastritis	positive	Not done
9	male	46	Erosive gastritis	positive	positive
10	male	70	Erosive gastritis	positive	Not done
11	female	59	Erosive gastritis	positive	Not done
12	male	41	Erosive gastritis	positive	Not done
13	male	64	Erosive gastritis	positive	Not done
14	male	43	Erosive gastritis	positive	positive
15	male	46	Erosive gastritis	positive	positive
16	male	63	Atrophic gastritis	positive	Not done
17	female	66	Atrophic gastritis	positive	Not done
18	male	65	Atrophic gastritis	positive	Not done
19	female	58	Atrophic gastritis	positive	positive
20	male	69	Atrophic gastritis	positive	Not done
21	female	70	Atrophic gastritis	positive	Not done
22	female	63	Atrophic gastritis	positive	Not done
23	female	67	Atrophic gastritis	positive	Not done
24	male	60	Atrophic gastritis	positive	Not done
25	male	54	Atrophic gastritis	positive	positive
26	female	57	Atrophic gastritis	positive	positive
27	male	45	Atrophic gastritis	positive	Not done
28	female	66	Atrophic gastritis	positive	positive
29	female	69	Atrophic gastritis	positive	positive
30	male	56	Atrophic gastritis	positive	Not done
31	male	41	Erosive gastritis	negative	Not done
32	female	41	Erosive gastritis	negative	Not done
33	male	44	Erosive gastritis	negative	negative
34	male	46	Erosive gastritis	negative	Not done
35	male	72	Erosive gastritis	negative	negative
36	female	52	Erosive gastritis	negative	Not done
37	female	48	Erosive gastritis	negative	negative
38	male	50	Erosive gastritis	negative	Not done
39	male	34	Erosive gastritis	negative	Not done

40	male	54	Erosive gastritis	negative	negative
41	female	60	Erosive gastritis	negative	Not done
42	male	39	Erosive gastritis	negative	Not done
43	male	53	Erosive gastritis	negative	negative
44	female	49	Erosive gastritis	negative	negative
45	male	32	Erosive gastritis	negative	Not done
46	female	43	Atrophic gastritis	negative	Not done
47	male	69	Atrophic gastritis	negative	negative
48	male	75	Atrophic gastritis	negative	Not done
49	female	67	Atrophic gastritis	negative	Not done
50	female	57	Atrophic gastritis	negative	Not done
51	female	59	Atrophic gastritis	negative	Not done
52	female	30	Atrophic gastritis	negative	negative
53	female	63	Atrophic gastritis	negative	negative
54	female	74	Atrophic gastritis	negative	Not done
55	male	39	Atrophic gastritis	negative	Not done
56	female	62	Atrophic gastritis	negative	Not done
57	female	69	Atrophic gastritis	negative	negative
58	male	45	Atrophic gastritis	negative	Not done
59	male	78	Atrophic gastritis	negative	negative
60	female	67	Atrophic gastritis	negative	Not done
