# Supplementary material for: Helicobacter pylori disrupts gastric mucosal homeostasis by stimulating macrophages to secrete CCL3
Source: Cell Commun Signal. 2024 May 10;22:263. doi: 10.1186/s12964-024-01627-5 (PMC11084090; doi:10.1186/s12964-024-01627-5)
Supplement: Supplementary file 3 — Additional file 3. [file 12964_2024_1627_MOESM3_ESM.pdf]

Fig.1E

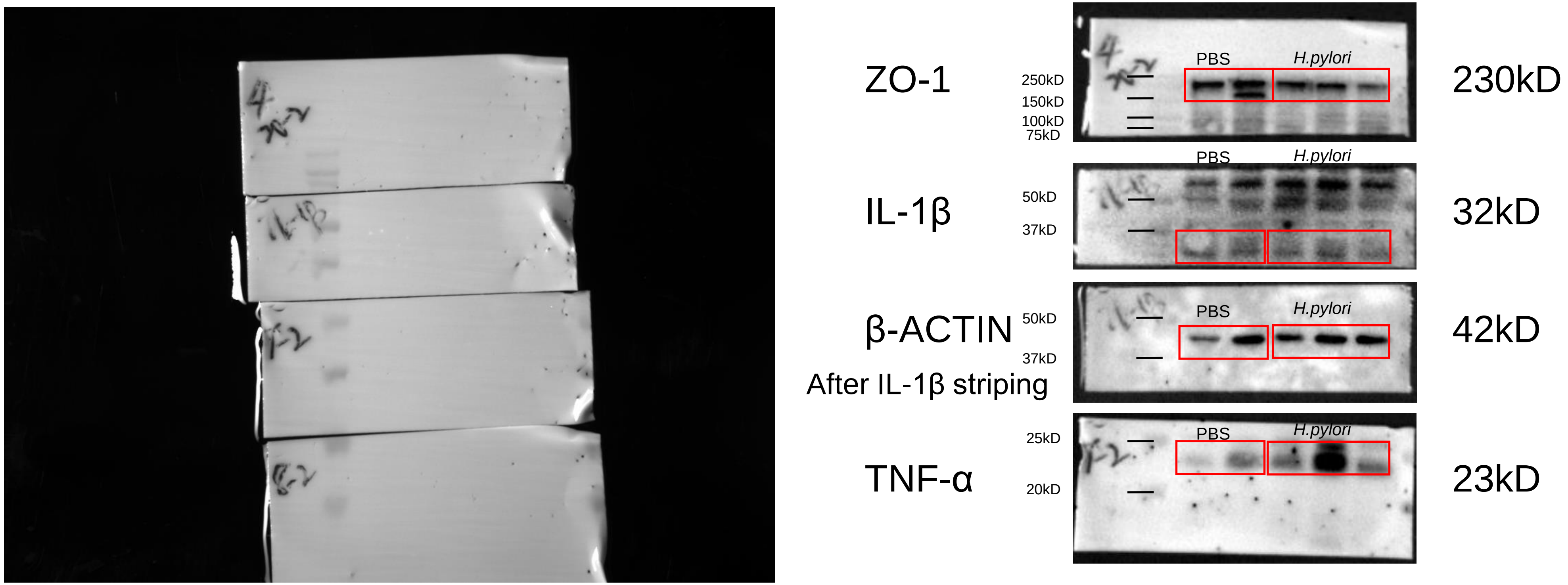
ZO-1
250kD
150kD
100kD
75kD
PBS
H.pylori
230kD
IL-1β
50kD
37kD
PBS
H.pylori
32kD
β-ACTIN
50kD
37kD
After IL-1β striping
PBS
H.pylori
42kD
TNF-α
25kD
20kD
PBS
H.pylori
23kD

Fig.1J

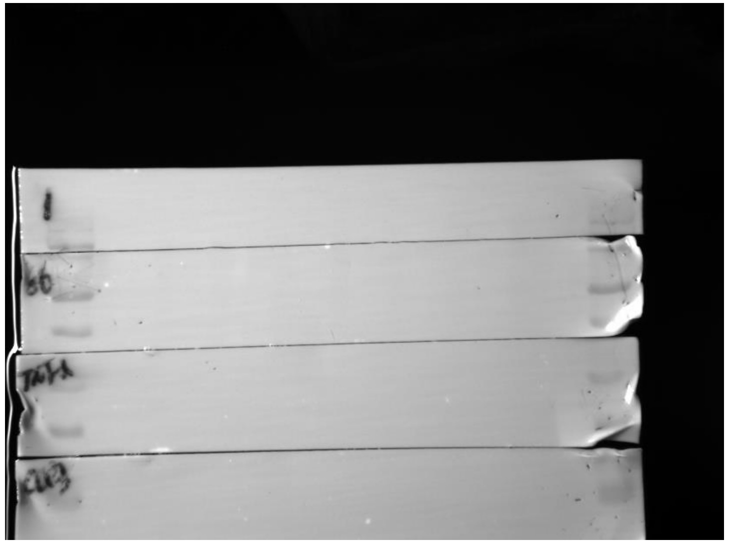

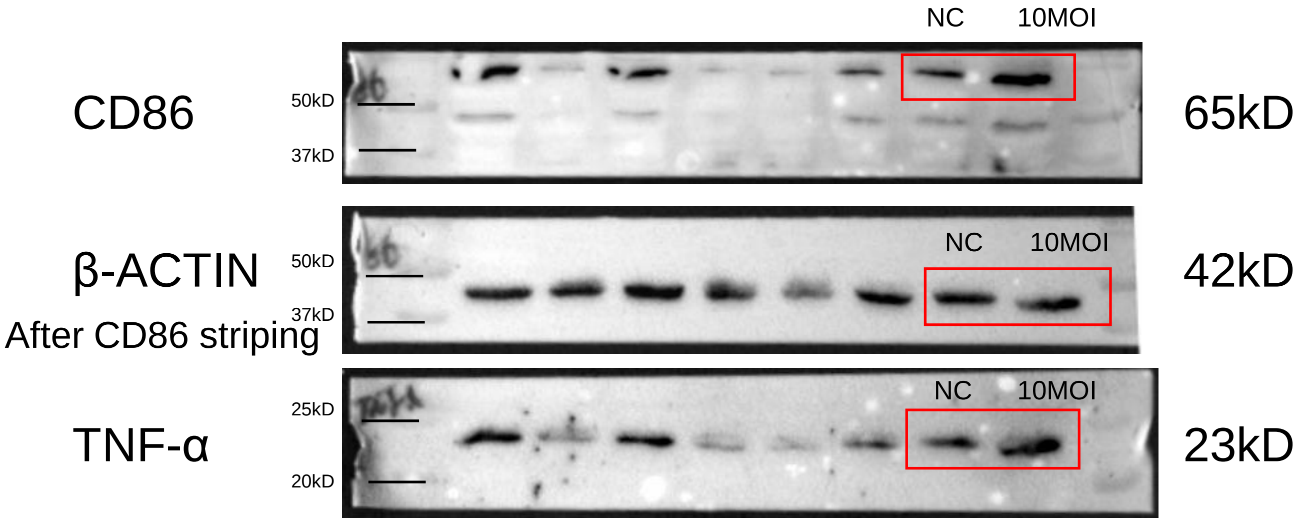
NC
10MOI
CD86
50kD
37kD
65kD
β-ACTIN
After CD86 striping
50kD
37kD
NC
10MOI
42kD
TNF-α
25kD
20kD
NC
10MOI
23kD

Fig.2B

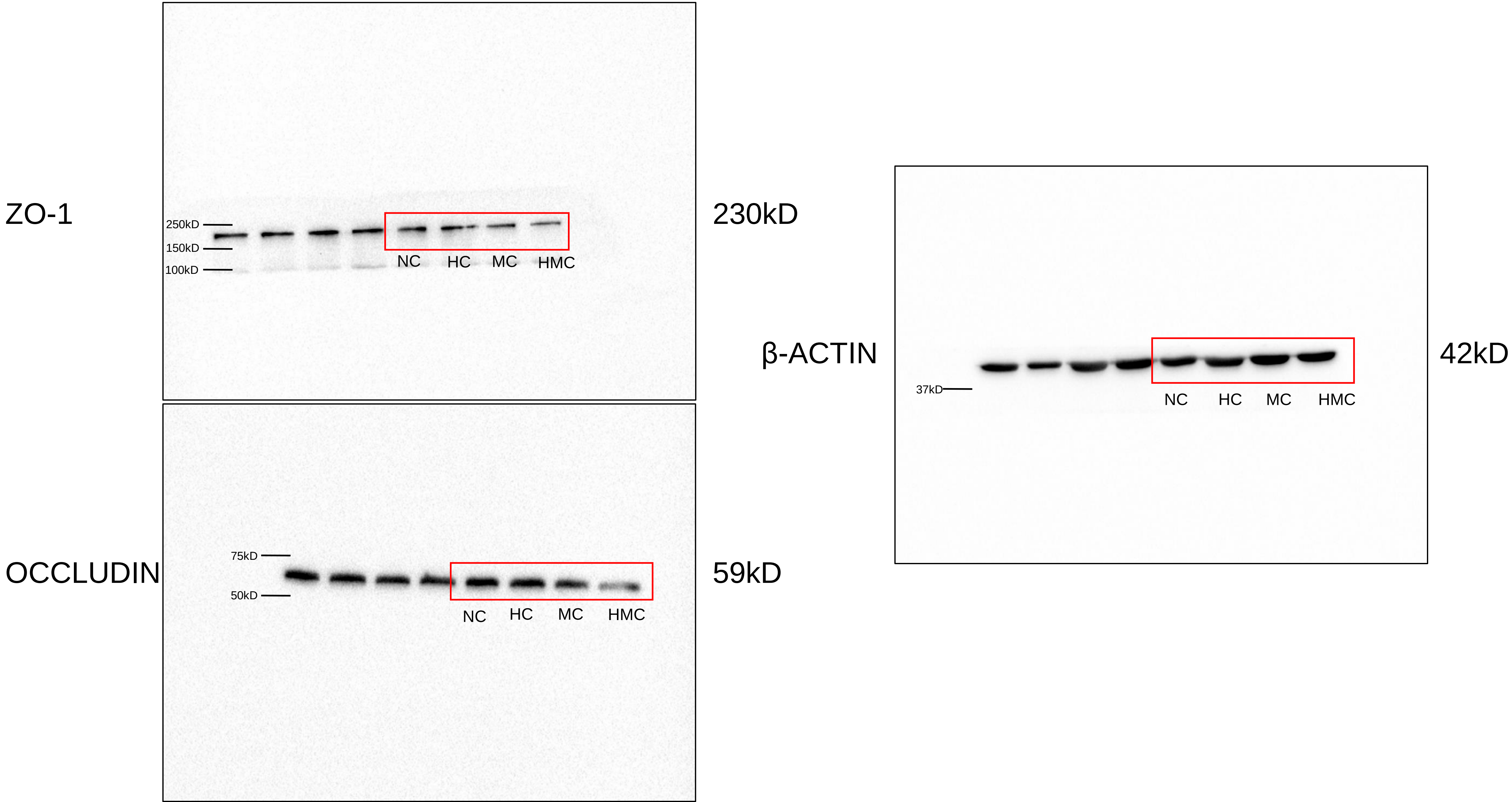
ZO-1
250kD
150kD
100kD
NC
HC
MC
HMC
230kD
OCCLUDIN
75kD
50kD
NC
HC
MC
HMC
59kD
β-ACTIN
37kD
NC
HC
MC
HMC
42kD

Fig.2C

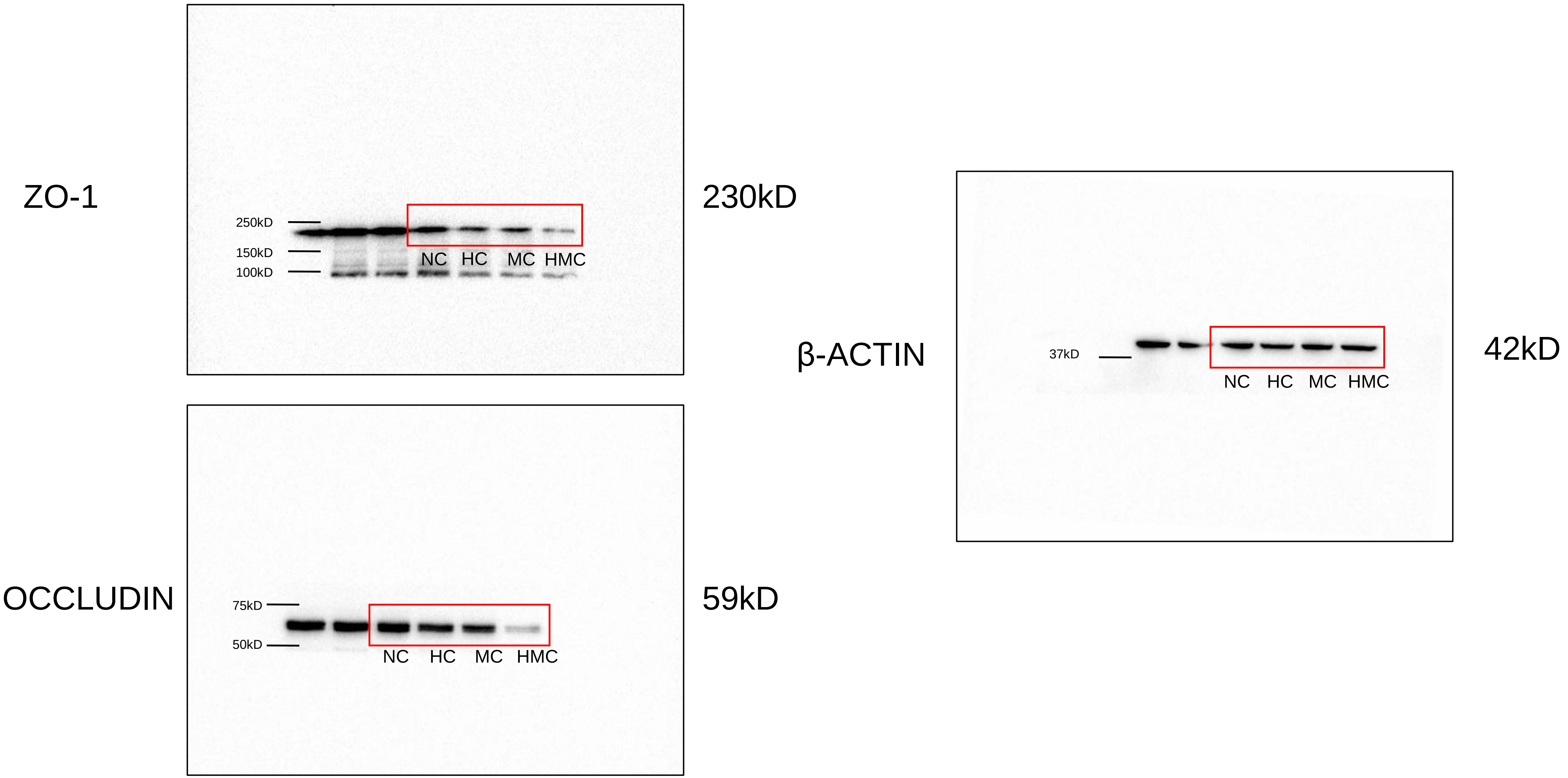
ZO-1
250kD
150kD
100kD
NC HC MC HMC
230kD
OCCLUDIN
75kD
50kD
NC HC MC HMC
59kD
β-ACTIN
37kD
NC HC MC HMC
42kD

Fig.2F

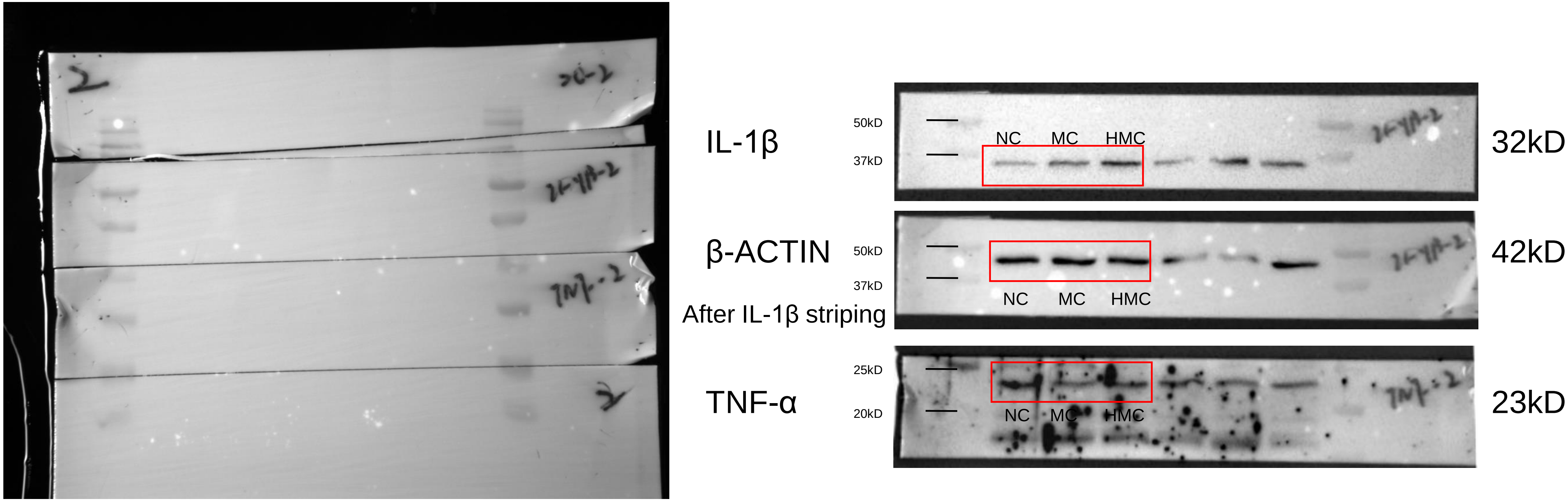
IL-1β
β-ACTIN
After IL-1β striping
TNF-α
50kD
37kD
50kD
37kD
25kD
20kD
NC
MC
HMC
NC
MC
HMC
NC
MC
HMC
32kD
42kD
23kD

Fig.3C

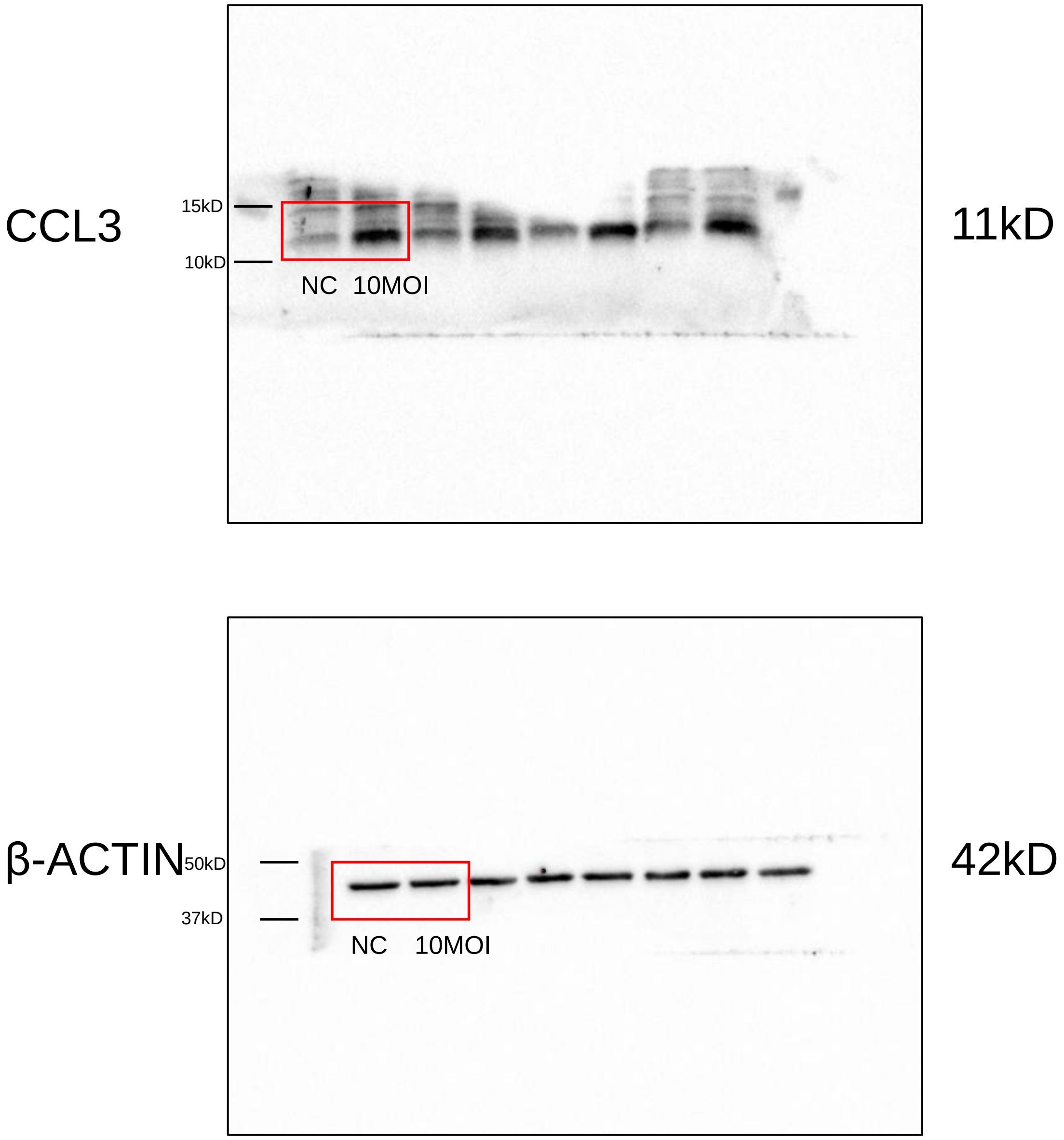
CCL3
15kD
10kD
NC 10MOI
11kD
β-ACTIN
50kD
37kD
NC 10MOI
42kD

Fig.3F

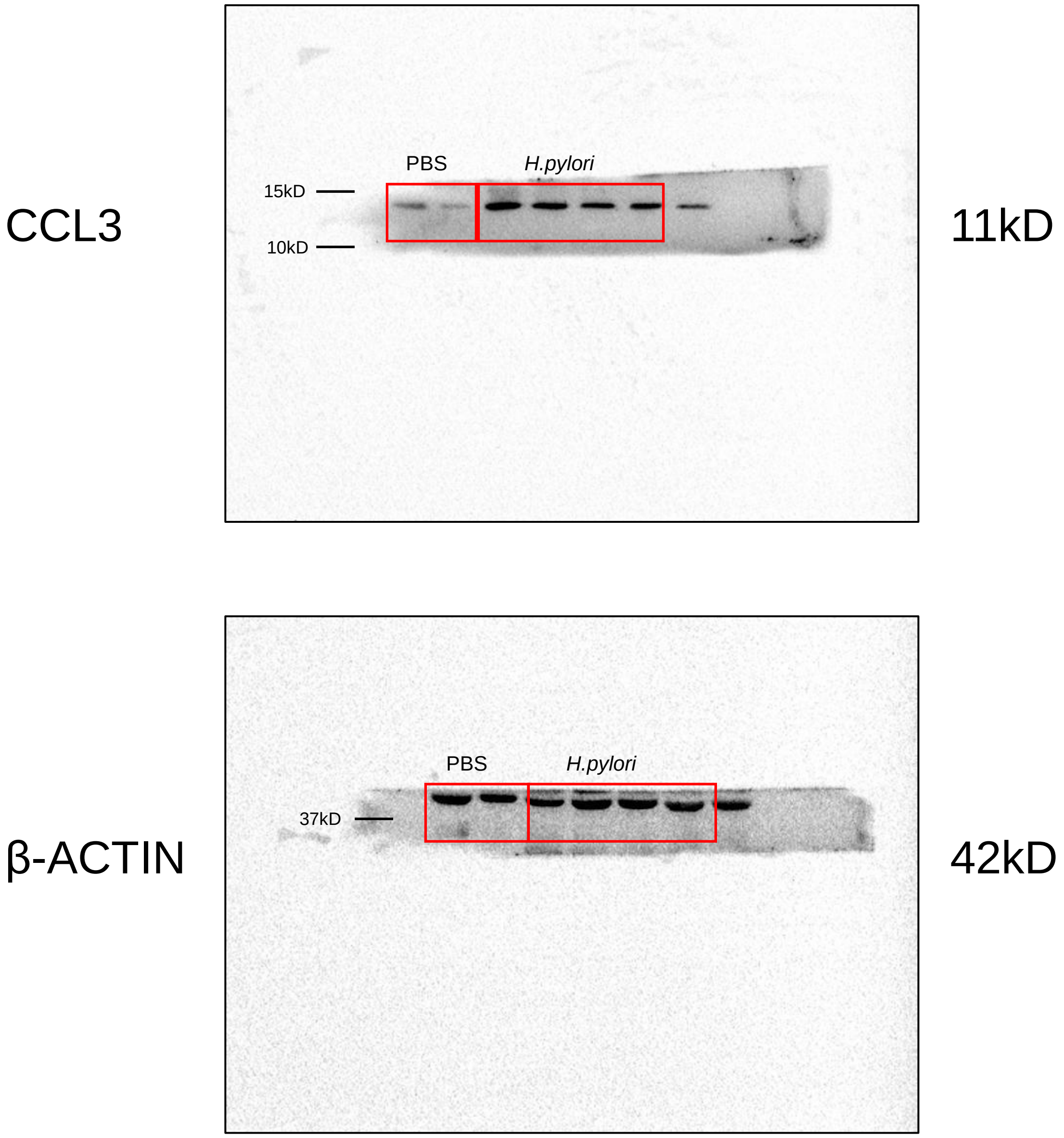
CCL3
PBS
H.pylori
15kD
10kD
11kD
β-ACTIN
PBS
H.pylori
37kD
42kD

Fig.4B

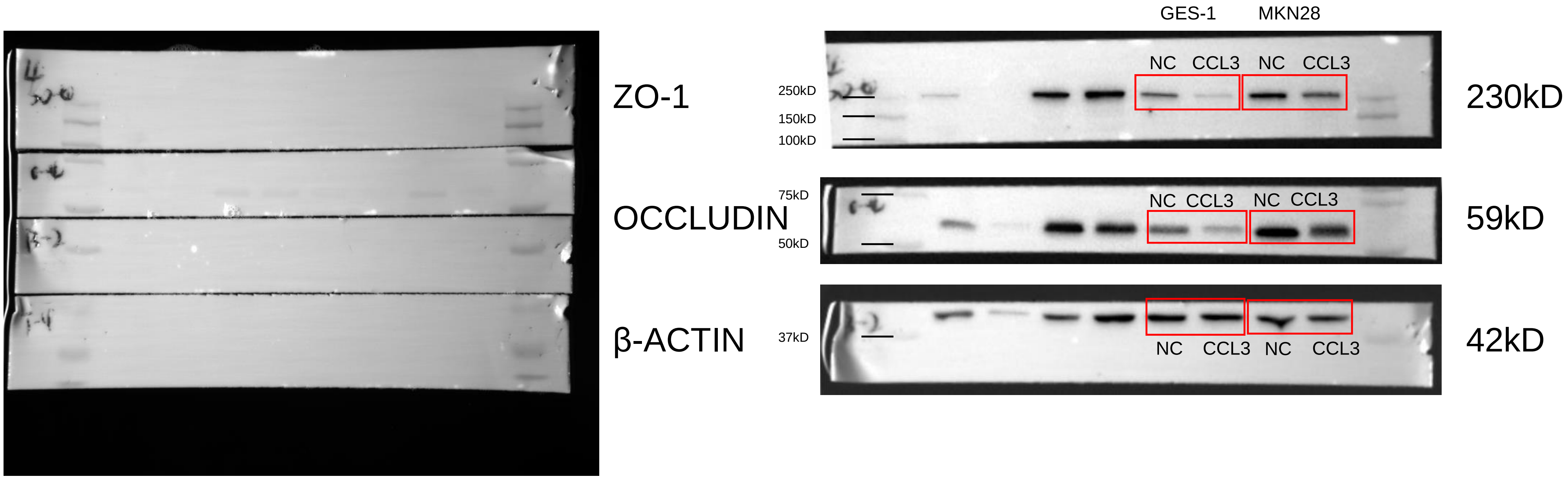
ZO-1
OCCLUDIN
β-ACTIN
GES-1
MKN28
NC
CCL3
NC
CCL3
250kD
150kD
100kD
230kD
NC
CCL3
NC
CCL3
75kD
50kD
59kD
37kD
NC
CCL3
NC
CCL3
42kD

Fig.4C

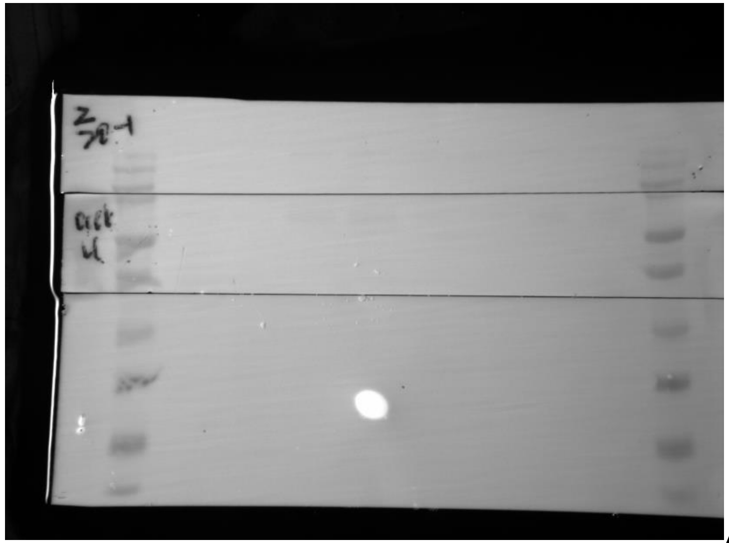

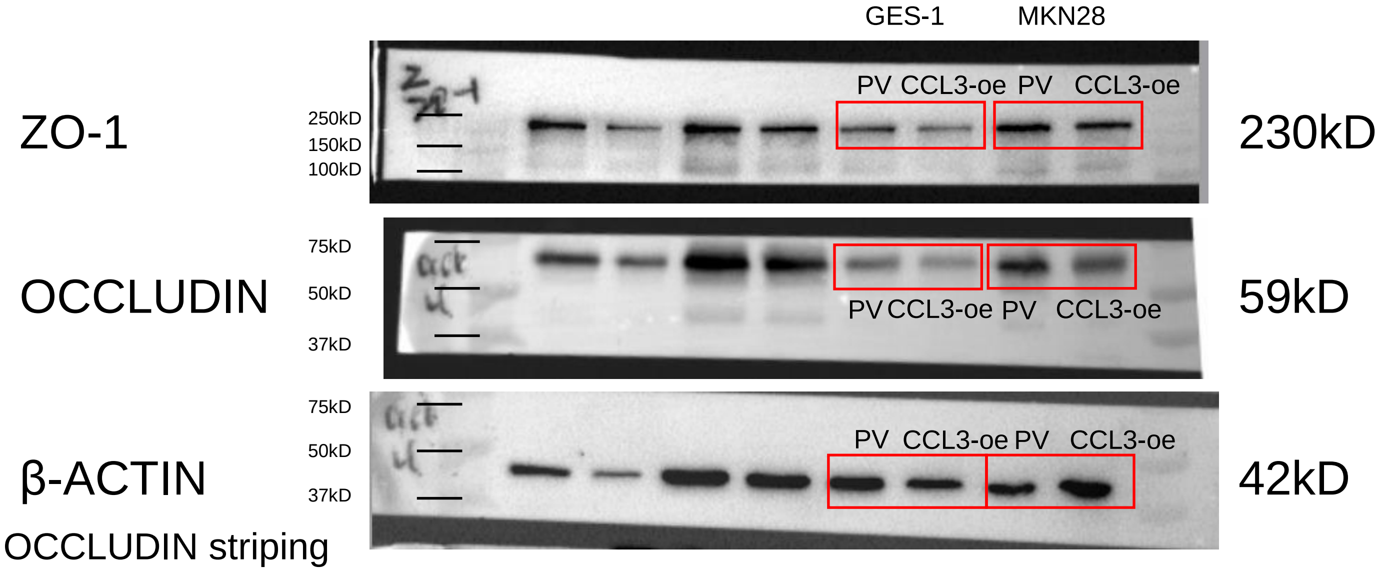
GES-1
MKN28
ZO-1
PV
CCL3-oe
PV
CCL3-oe
250kD
150kD
100kD
230kD
OCCLUDIN
75kD
50kD
37kD
PV
CCL3-oe
PV
CCL3-oe
59kD
β-ACTIN
75kD
50kD
37kD
PV
CCL3-oe
PV
CCL3-oe
42kD
After OCCLUDIN striping

Fig.5B

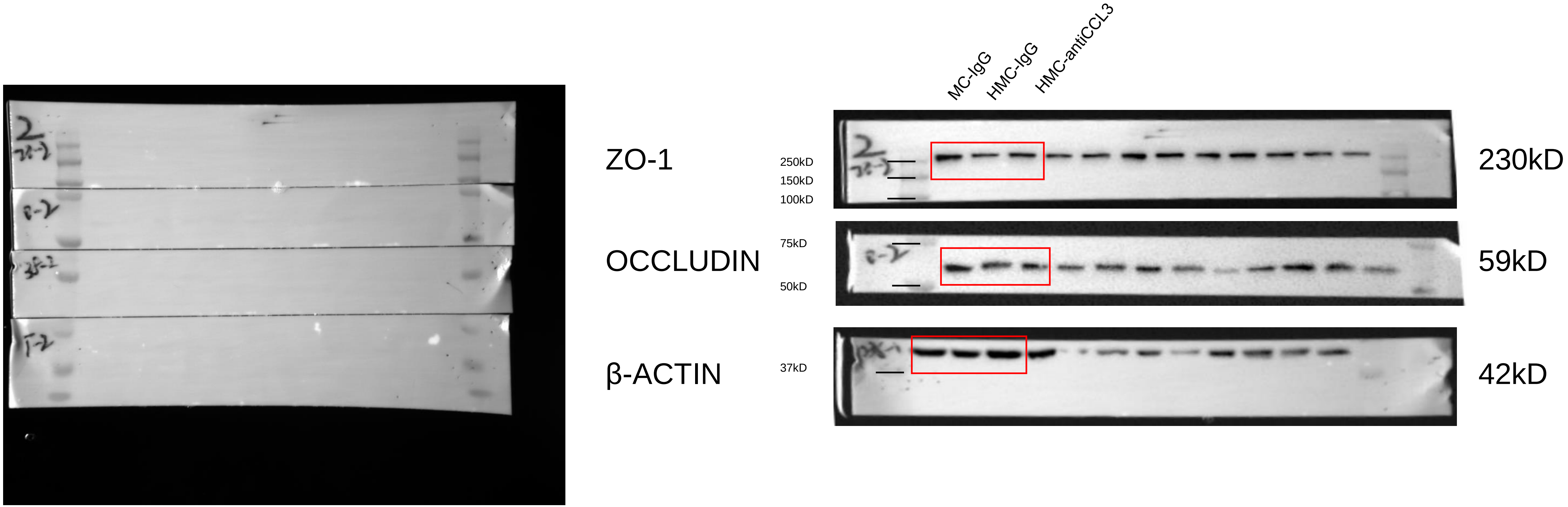
MC-IgG
HMC-IgG
HMC-antiCCL3
ZO-1
OCCLUDIN
β-ACTIN
250kD
150kD
100kD
75kD
50kD
37kD
230kD
59kD
42kD

Fig.5G

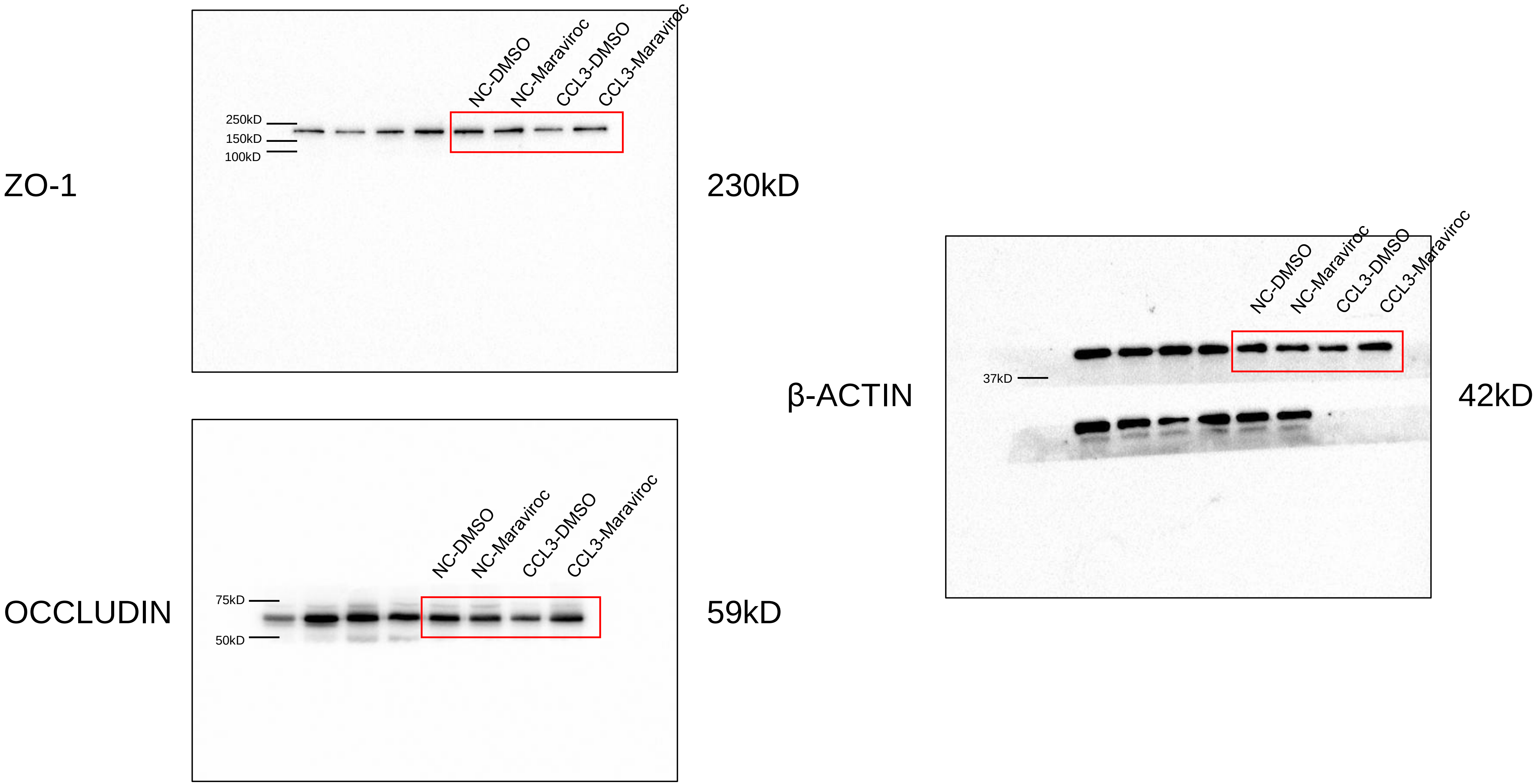
ZO-1
NC-DMSO
NC-Maraviroc
CCL3-DMSO
CCL3-Maraviroc
250kD
150kD
100kD
230kD
OCCLUDIN
NC-DMSO
NC-Maraviroc
CCL3-DMSO
CCL3-Maraviroc
75kD
50kD
59kD
β-ACTIN
NC-DMSO
NC-Maraviroc
CCL3-DMSO
CCL3-Maraviroc
37kD
42kD

Fig.6B

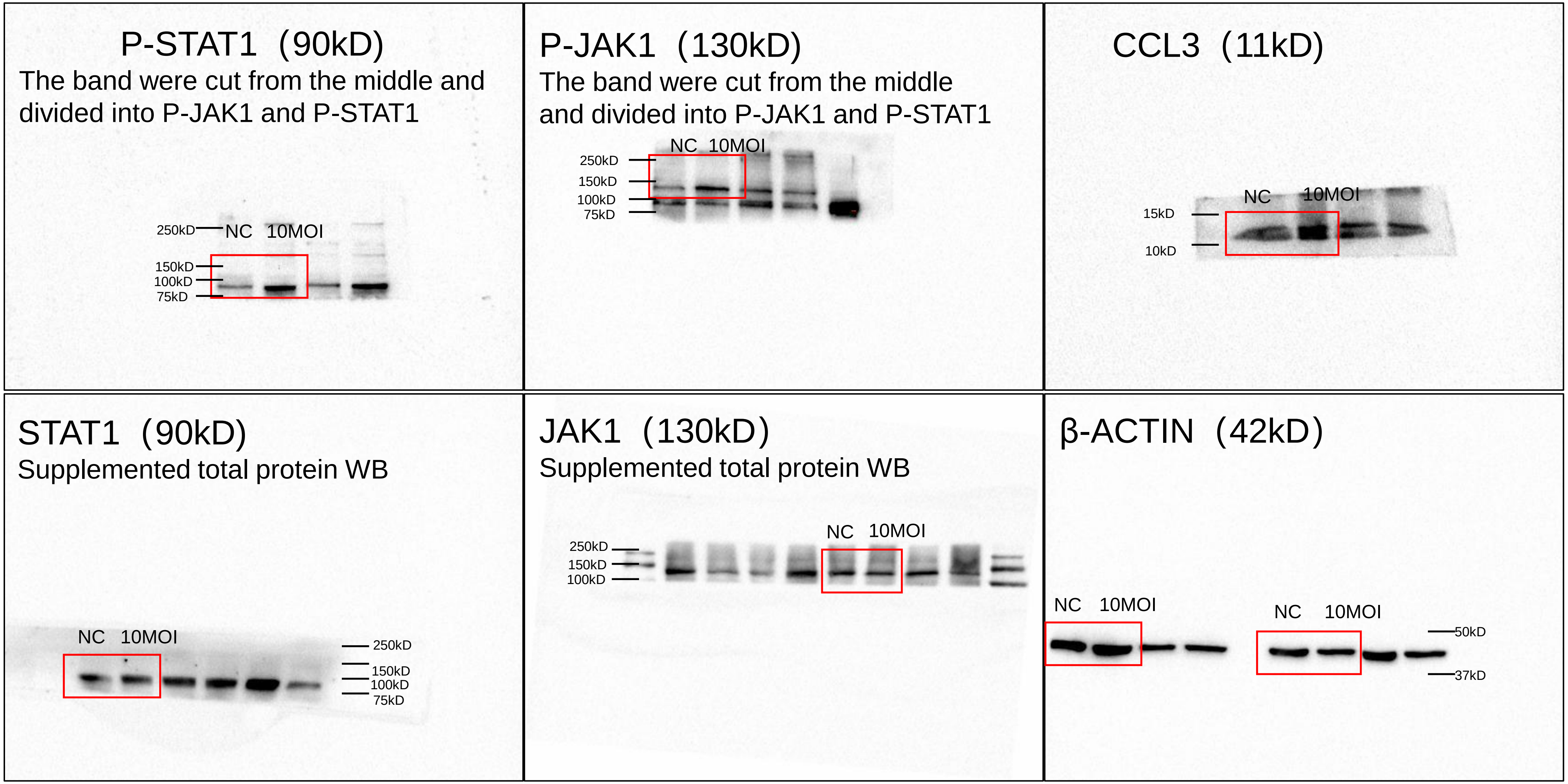
P-STAT1（90kD)
The band were cut from the middle and divided into P-JAK1 and P-STAT1
250kD
NC 10MOI
150kD
100kD
75kD
P-JAK1（130kD)
The band were cut from the middle and divided into P-JAK1 and P-STAT1
NC 10MOI
250kD
150kD
100kD
75kD
CCL3（11kD)
NC 10MOI
15kD
10kD
STAT1（90kD)
Supplemented total protein WB
NC 10MOI
250kD
150kD
100kD
75kD
JAK1（130kD)
Supplemented total protein WB
NC 10MOI
250kD
150kD
100kD
β-ACTIN（42kD)
NC 10MOI
NC 10MOI
50kD
37kD

Fig.6C

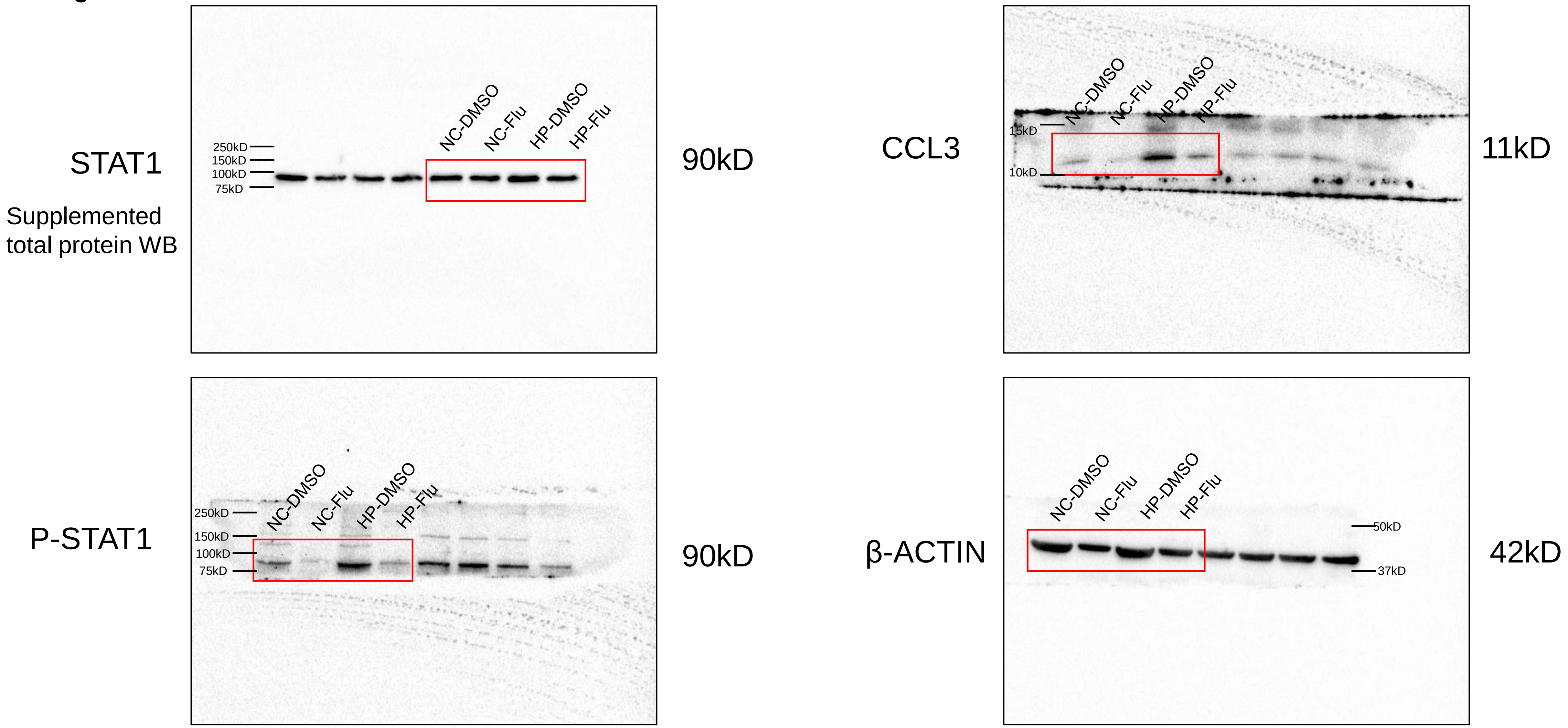
STAT1
Supplemented total protein WB
250kD
150kD
100kD
75kD
NC-DMSO
NC-Flu
HP-DMSO
HP-Flu
90kD
CCL3
15kD
10kD
11kD
P-STAT1
90kD
β-ACTIN
50kD
37kD
42kD

Fig.6D

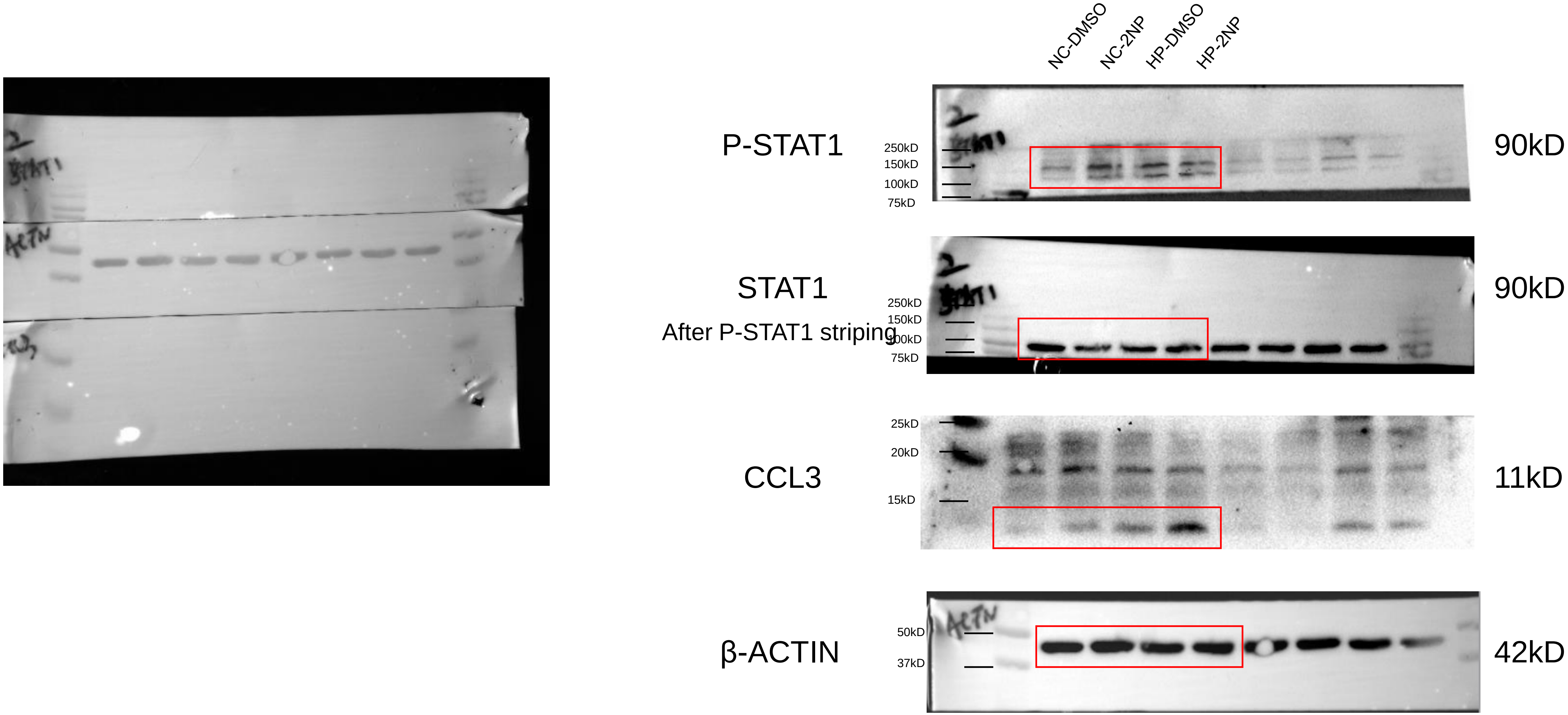
NC-DMSO
NC-2NP
HP-DMSO
HP-2NP
P-STAT1
250kD
150kD
100kD
75kD
90kD
STAT1
After P-STAT1 striping
250kD
150kD
100kD
75kD
90kD
CCL3
25kD
20kD
15kD
11kD
β-ACTIN
50kD
37kD
42kD

Fig.6F

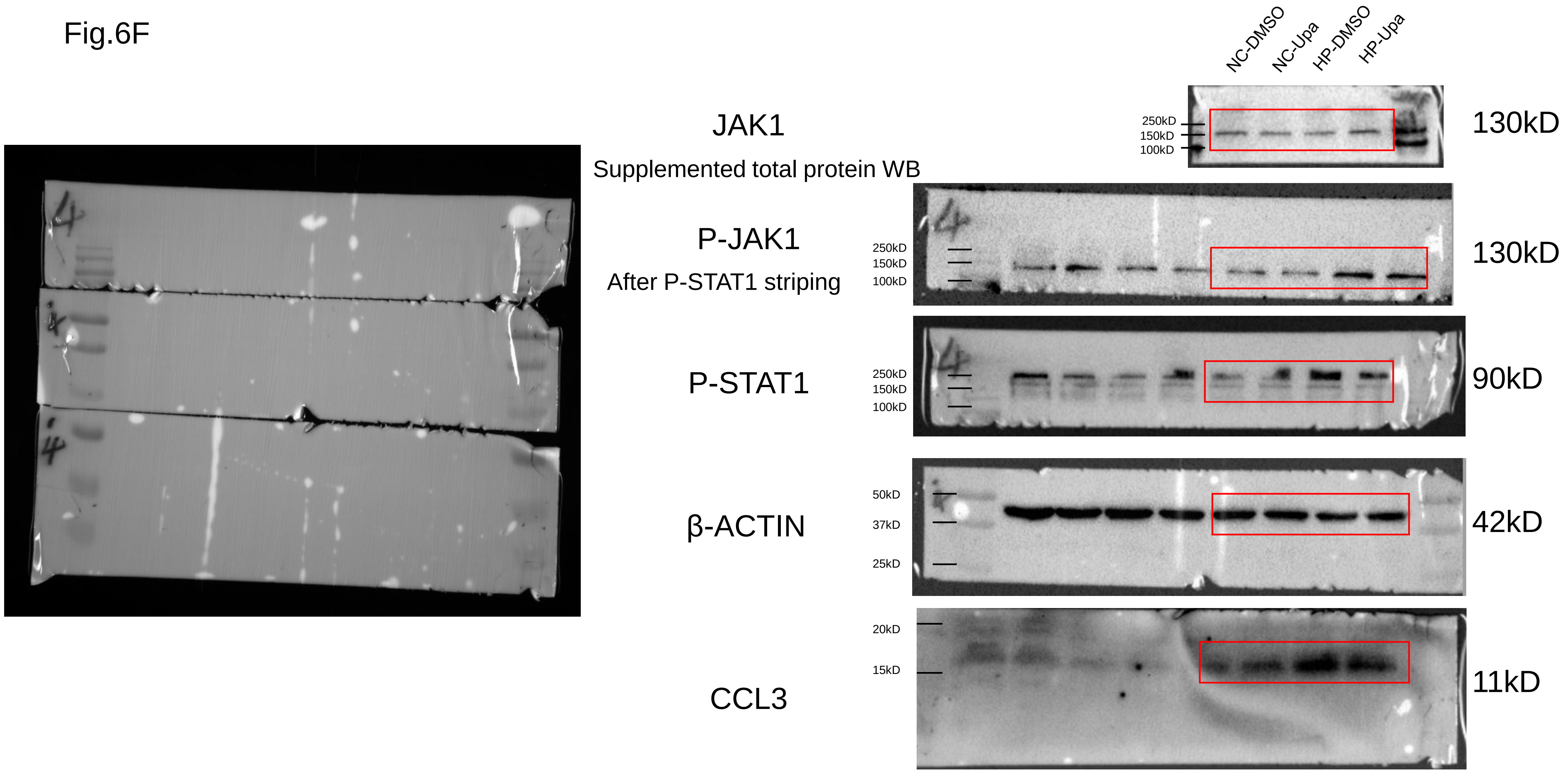
NC-DMSO
NC-Upa
HP-DMSO
HP-Upa
JAK1
250kD
150kD
100kD
130kD
Supplemented total protein WB
P-JAK1
After P-STAT1 striping
250kD
150kD
100kD
130kD
P-STAT1
250kD
150kD
100kD
90kD
β-ACTIN
50kD
37kD
25kD
42kD
CCL3
20kD
15kD
11kD

Fig.7A

P-P38

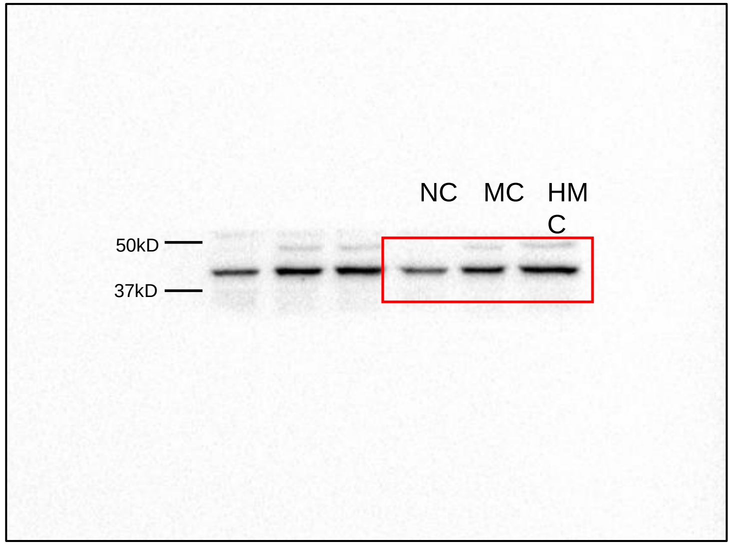

38kD

P38

After P-P38 striping

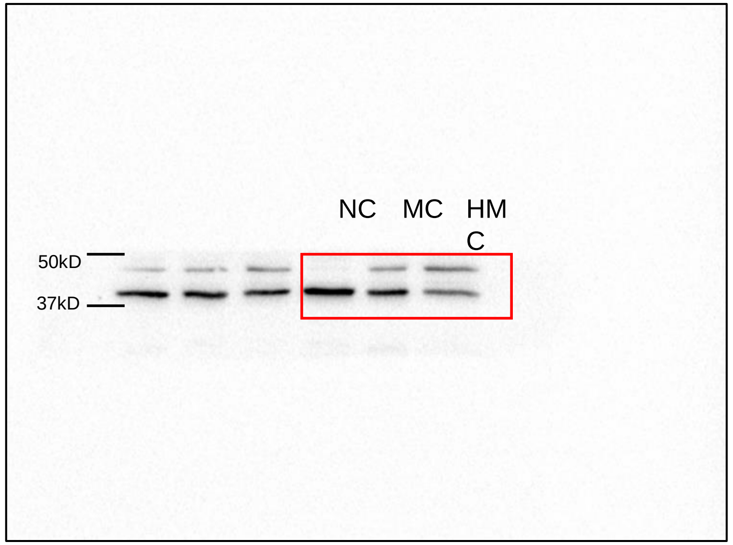

38kD

β-ACTIN

After P38 striping

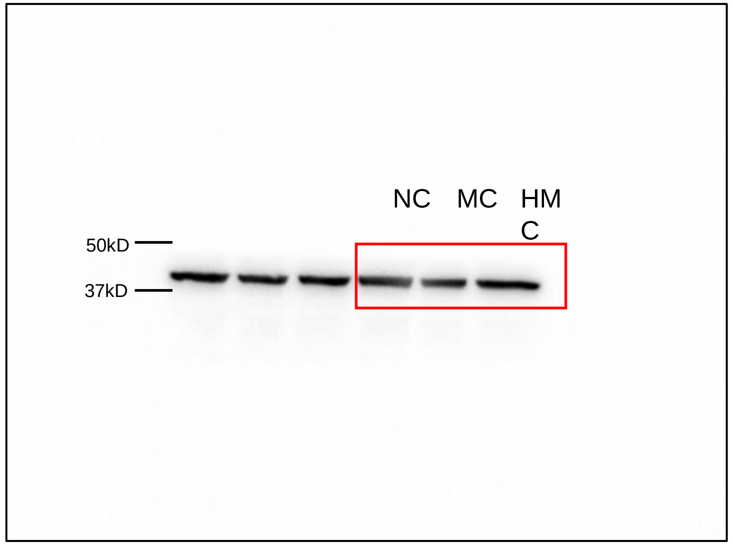

42kD

Fig.7C-D

P-P38

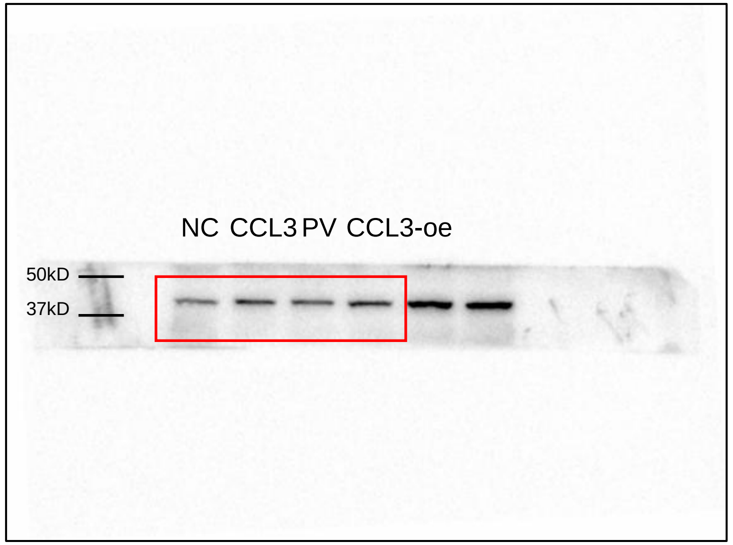

38kD

P38

After P-P38 striping

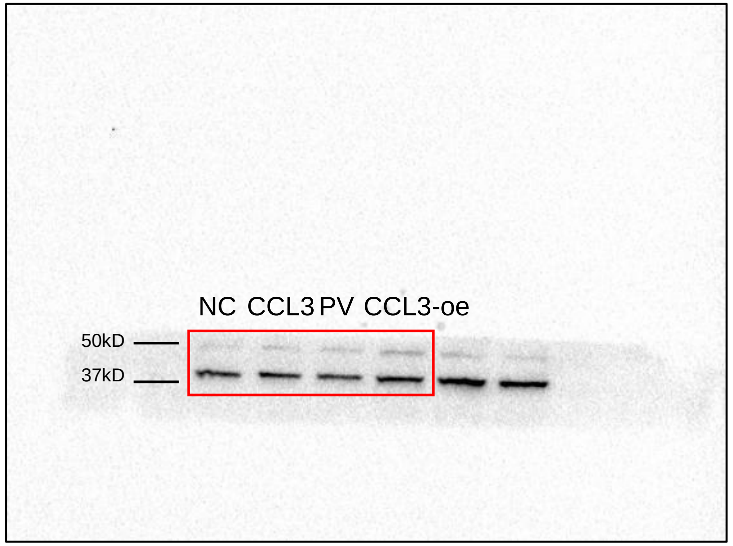

38kD

β-ACTIN

After P38 striping

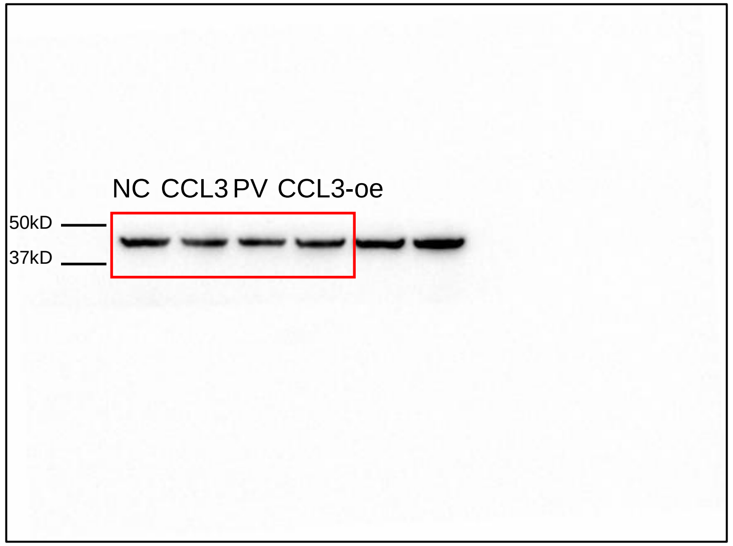

42kD

Fig.7E

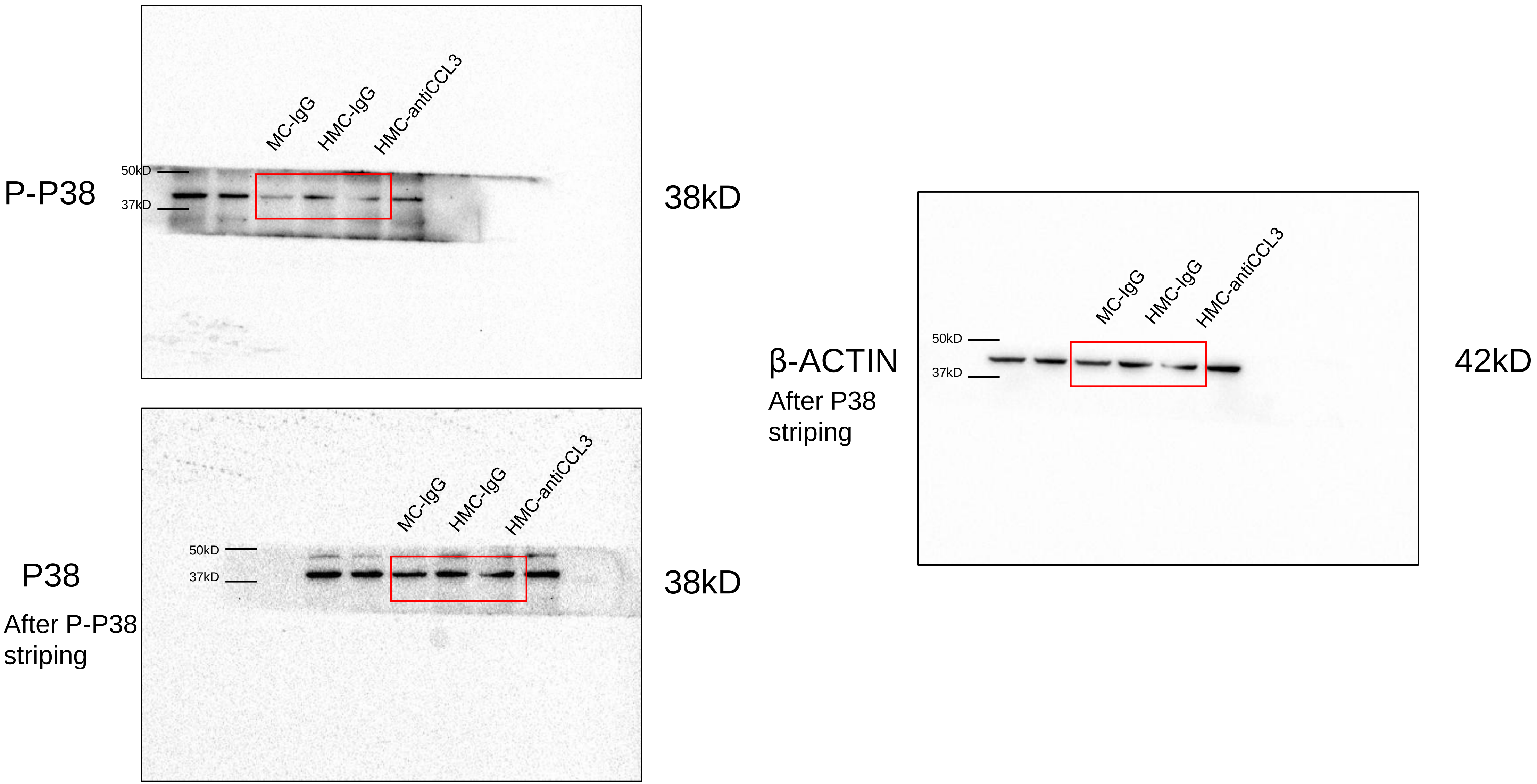
P-P38
MC-IgG
HMC-IgG
HMC-antiCCL3
50kD
37kD
38kD
P38
After P-P38
striping
MC-IgG
HMC-IgG
HMC-antiCCL3
50kD
37kD
38kD
β-ACTIN
After P38
striping
MC-IgG
HMC-IgG
HMC-antiCCL3
50kD
37kD
42kD

Fig.7F

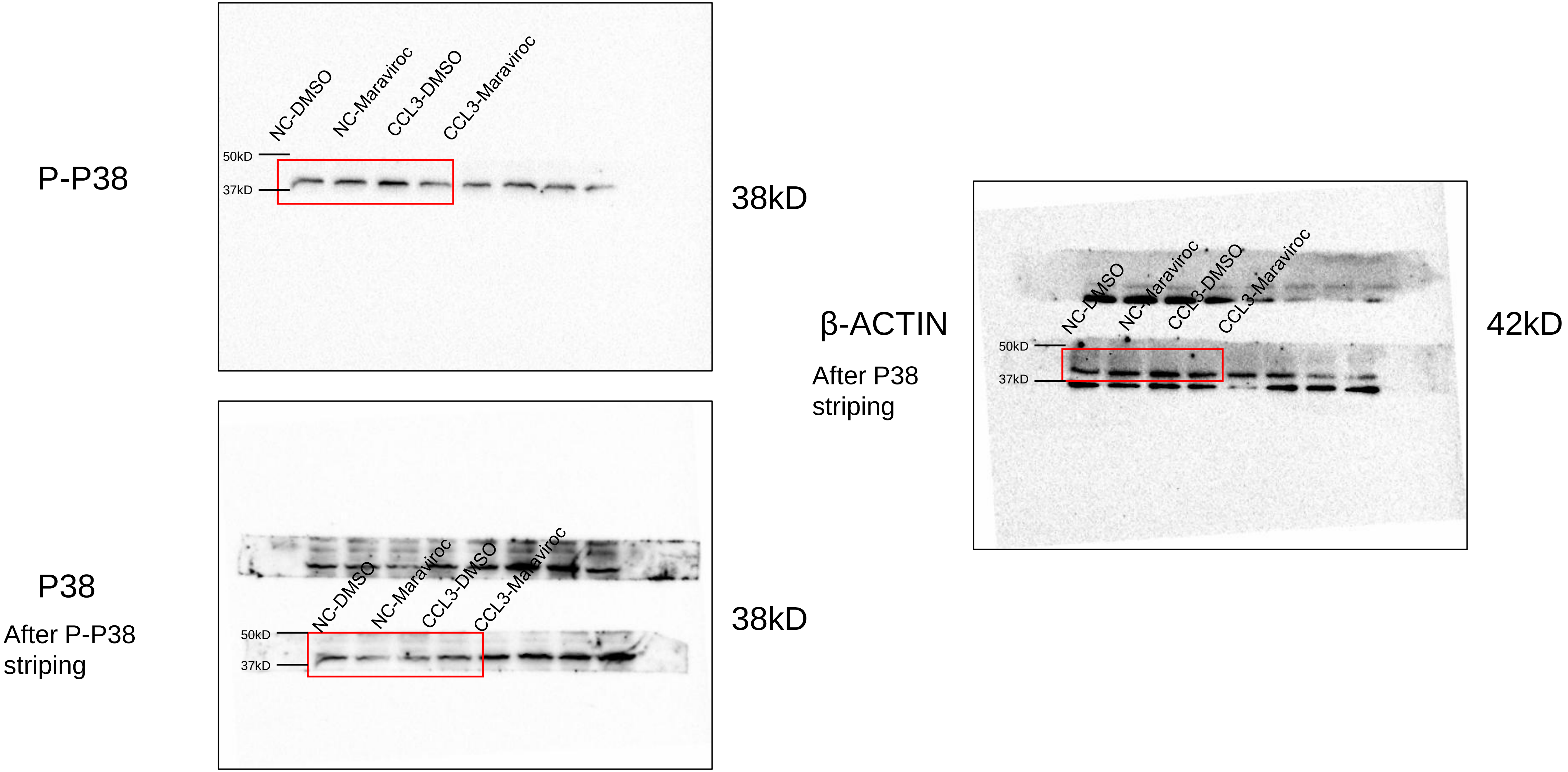
P-P38
NC-DMSO
NC-Maraviroc
CCL3-DMSO
CCL3-Maraviroc
50kD
37kD
38kD
P38
After P-P38 striping
NC-DMSO
NC-Maraviroc
CCL3-DMSO
CCL3-Maraviroc
50kD
37kD
38kD
β-ACTIN
After P38 striping
NC-DMSO
NC-Maraviroc
CCL3-DMSO
CCL3-Maraviroc
50kD
37kD
42kD

Fig.7I

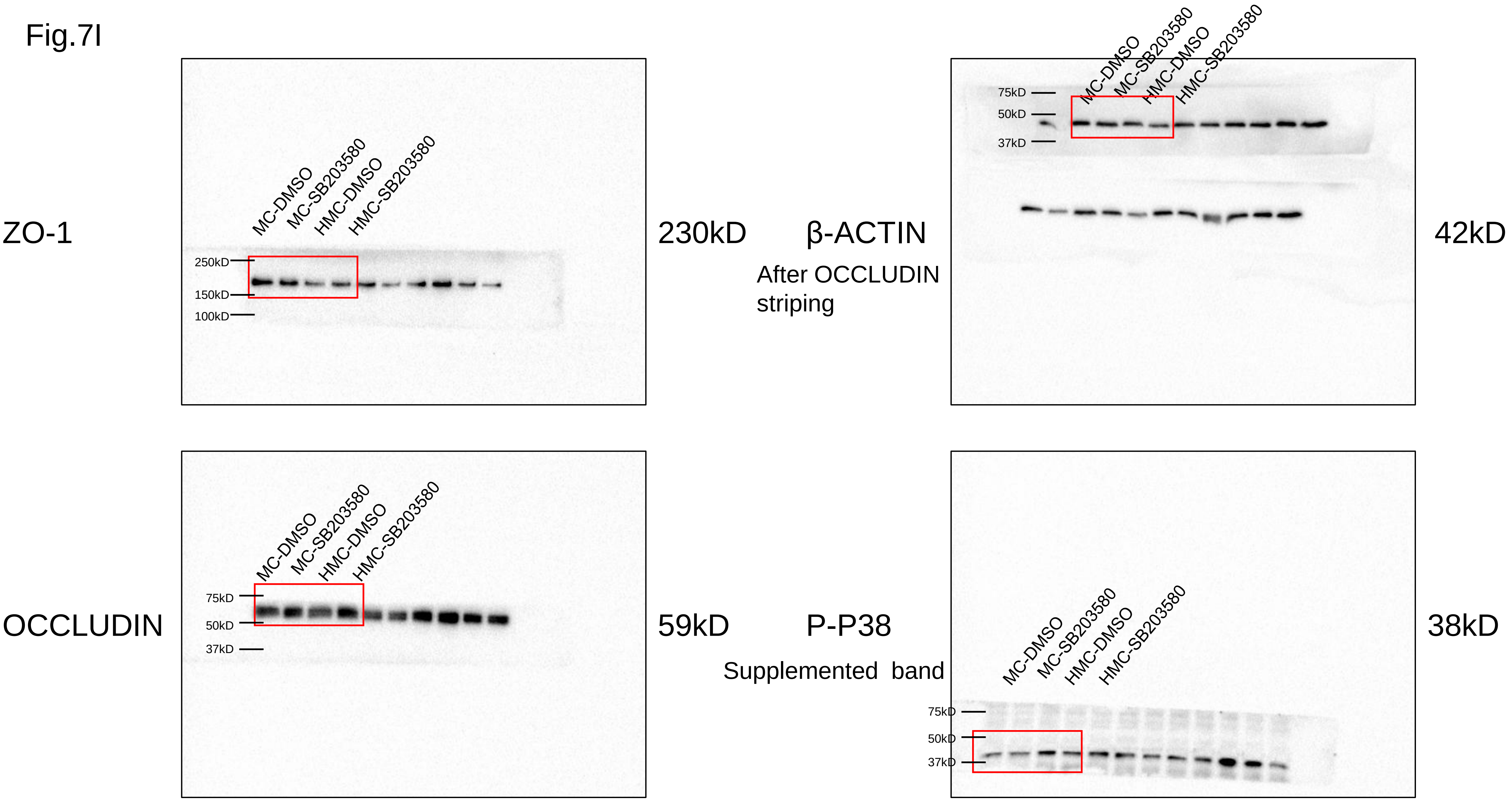
ZO-1
MC-DMSO
MC-SB203580
HMC-DMSO
HMC-SB203580
250kD
150kD
100kD
230kD
β-ACTIN
After OCCLUDIN striping
75kD
50kD
37kD
42kD
OCCLUDIN
75kD
50kD
37kD
59kD
P-P38
Supplemented band
75kD
50kD
37kD
38kD

Fig.7J

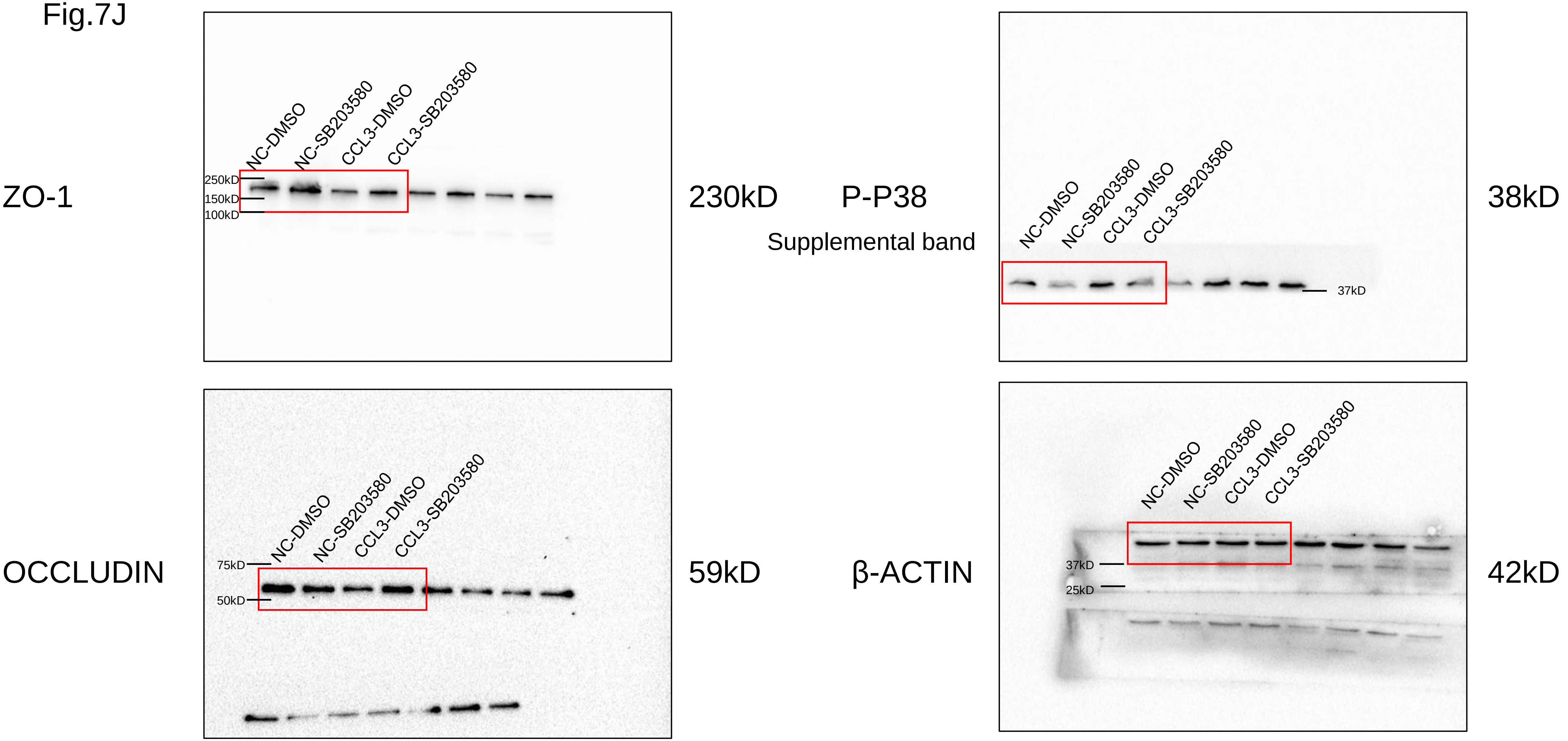
ZO-1
230kD
NC-DMSO
NC-SB203580
CCL3-DMSO
CCL3-SB203580
250kD
150kD
100kD
P-P38
Supplemental band
38kD
37kD
OCCLUDIN
59kD
75kD
50kD
β-ACTIN
42kD
37kD
25kD

Fig.8E,8G(The same sample ran back-to-back and incubated with different antibodies)

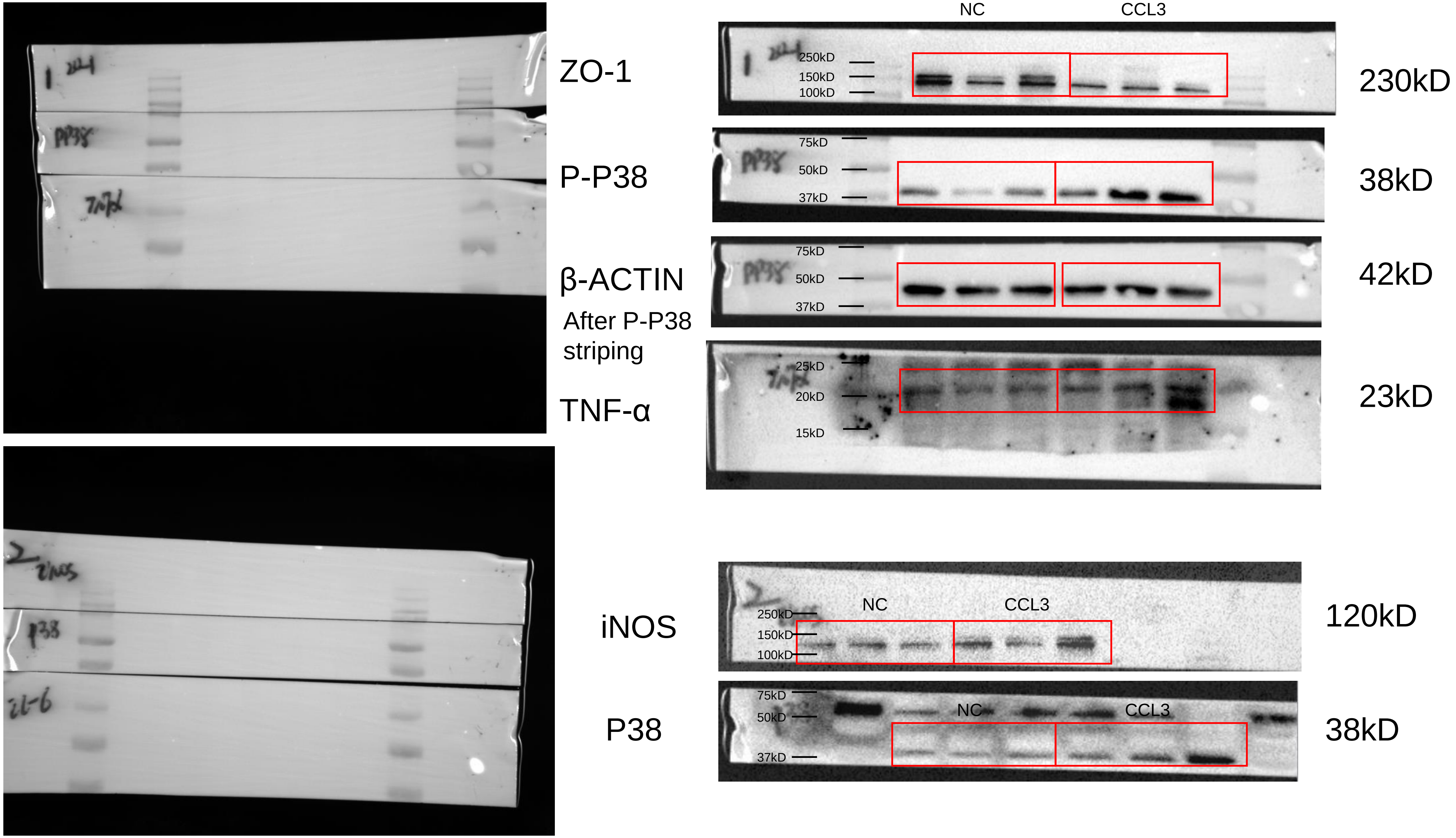

Fig.8K(The same sample ran back-to-back and incubated with different antibodies)

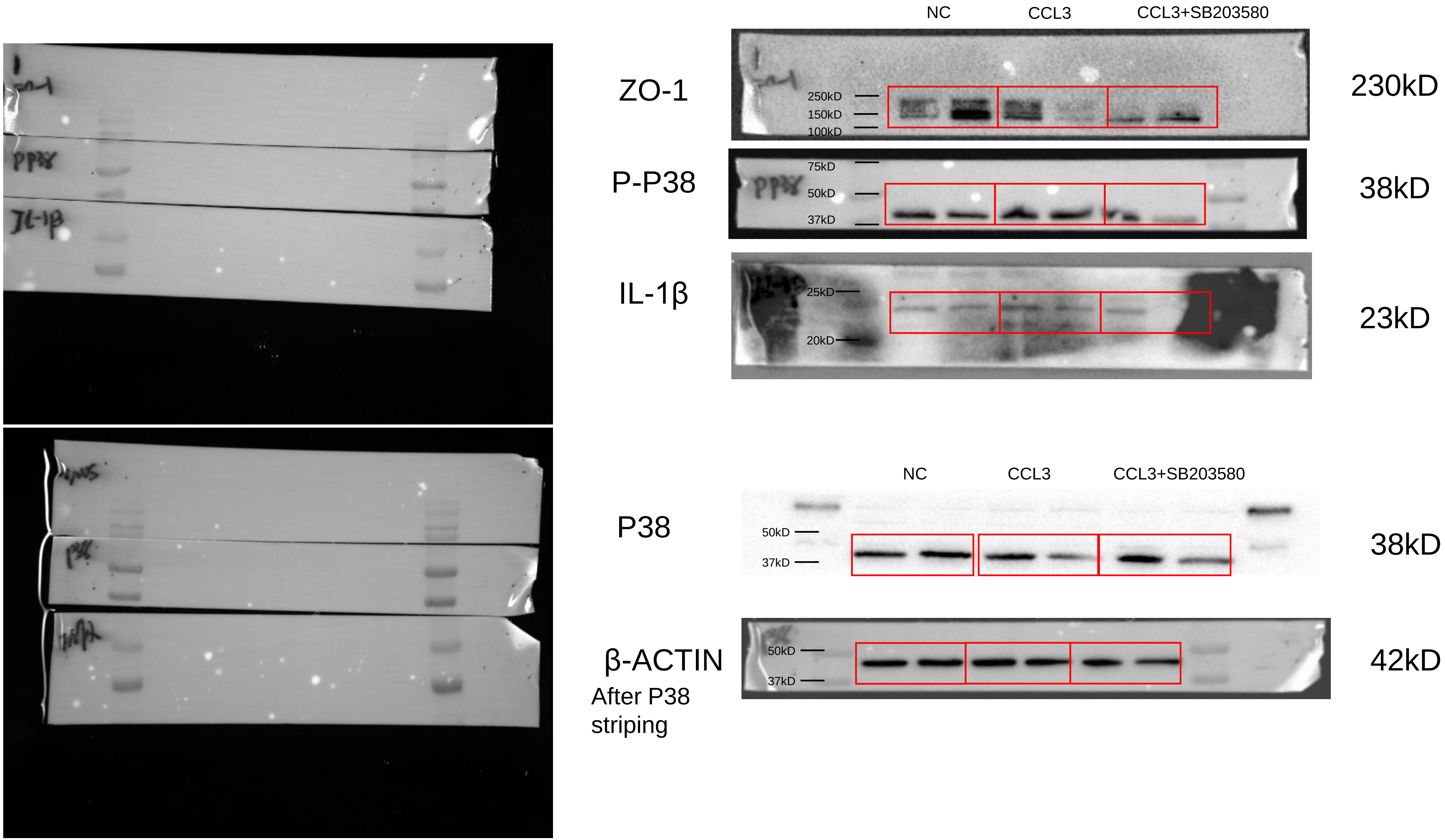

SP-Fig.1B

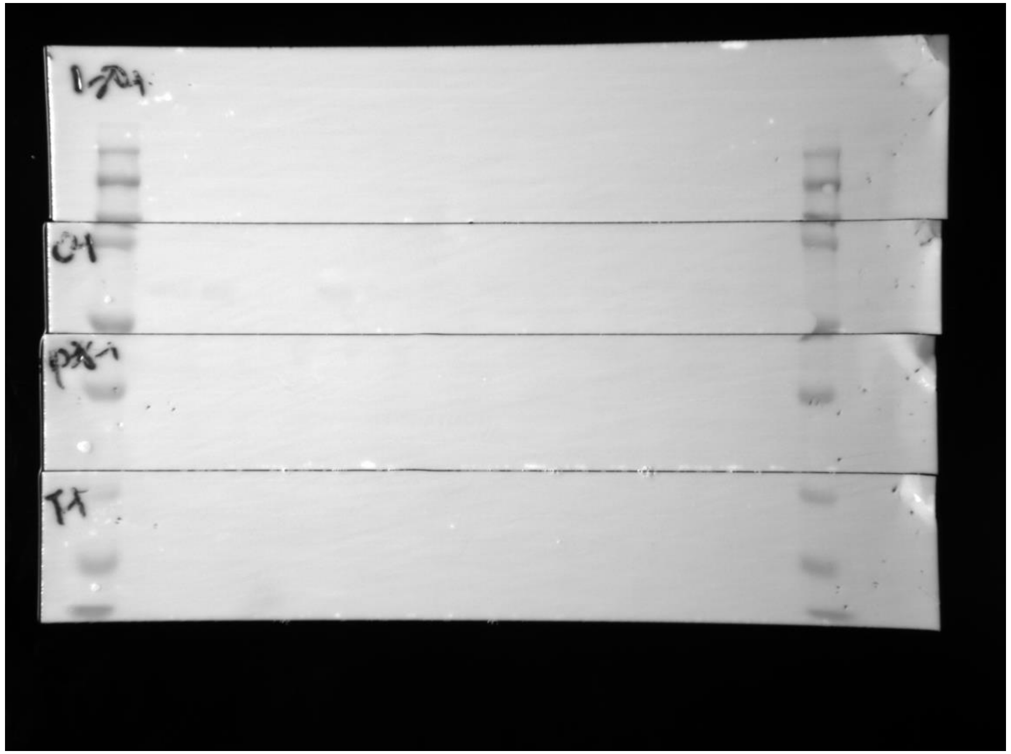

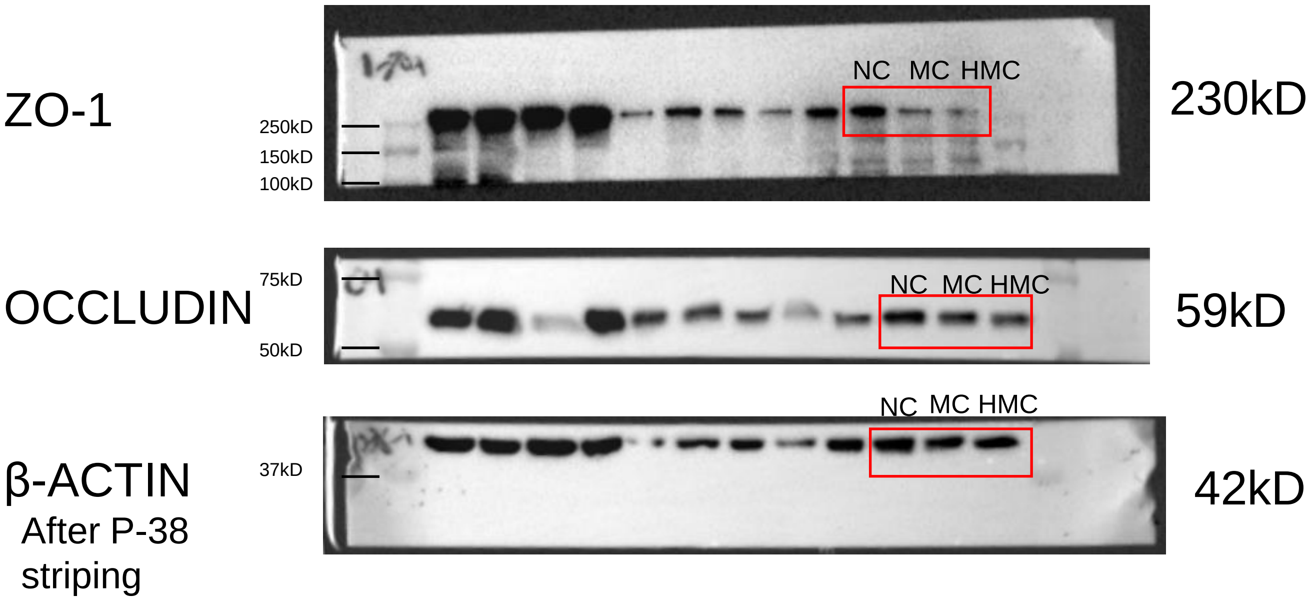
ZO-1
OCCLUDIN
β-ACTIN
After P-38
striping
250kD
150kD
100kD
75kD
50kD
37kD
NC MC HMC
NC MC HMC
NC MC HMC
230kD
59kD
42kD

SP-Fig.1C

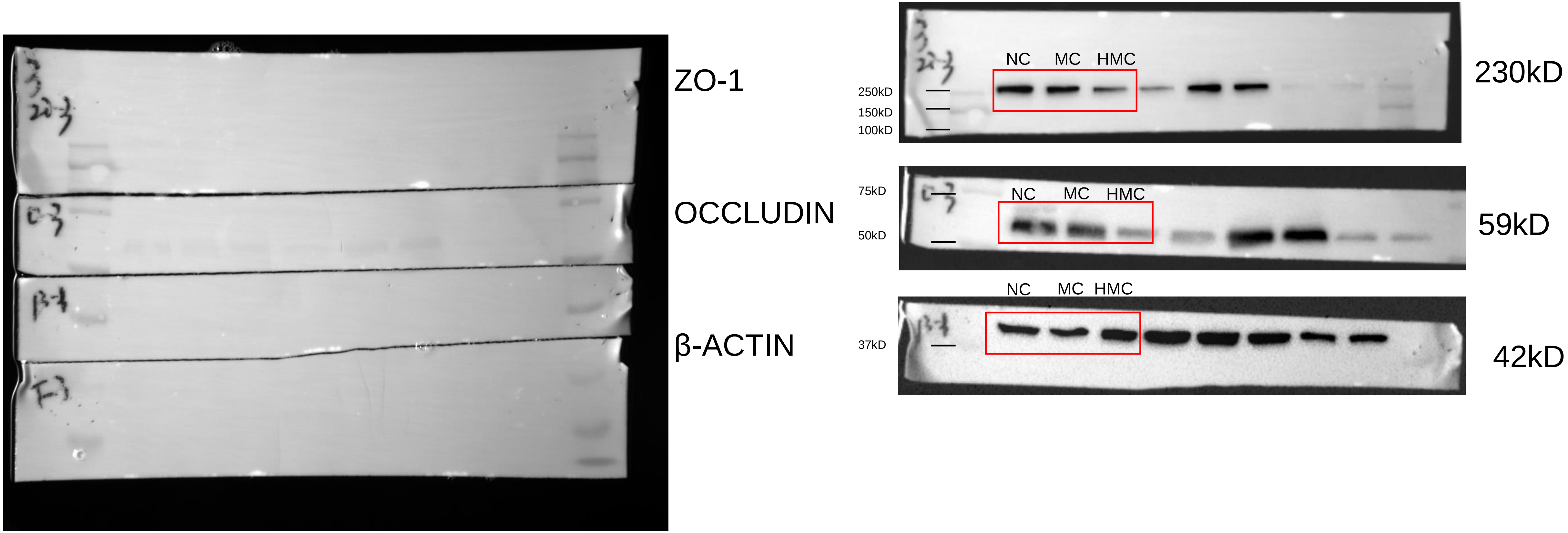
ZO-1
OCCLUDIN
β-ACTIN
NC
MC
HMC
250kD
150kD
100kD
230kD
75kD
50kD
59kD
37kD
42kD

SP-Fig.4B

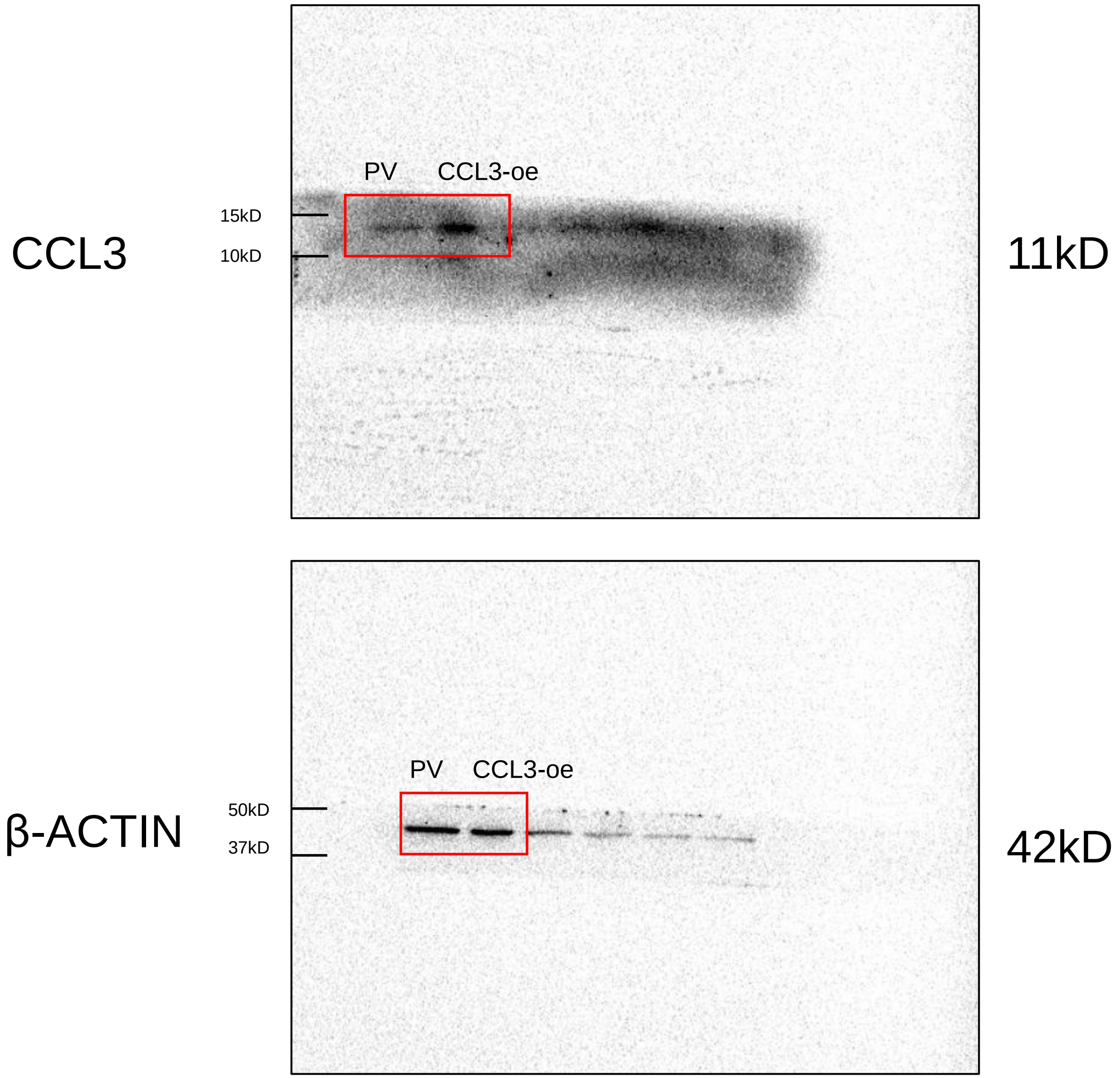
CCL3
15kD
10kD
PV
CCL3-oe
11kD
β-ACTIN
50kD
37kD
PV
CCL3-oe
42kD

SP-Fig.5A

The band were cut from the middle and divided into STAT1 and P-STAT1

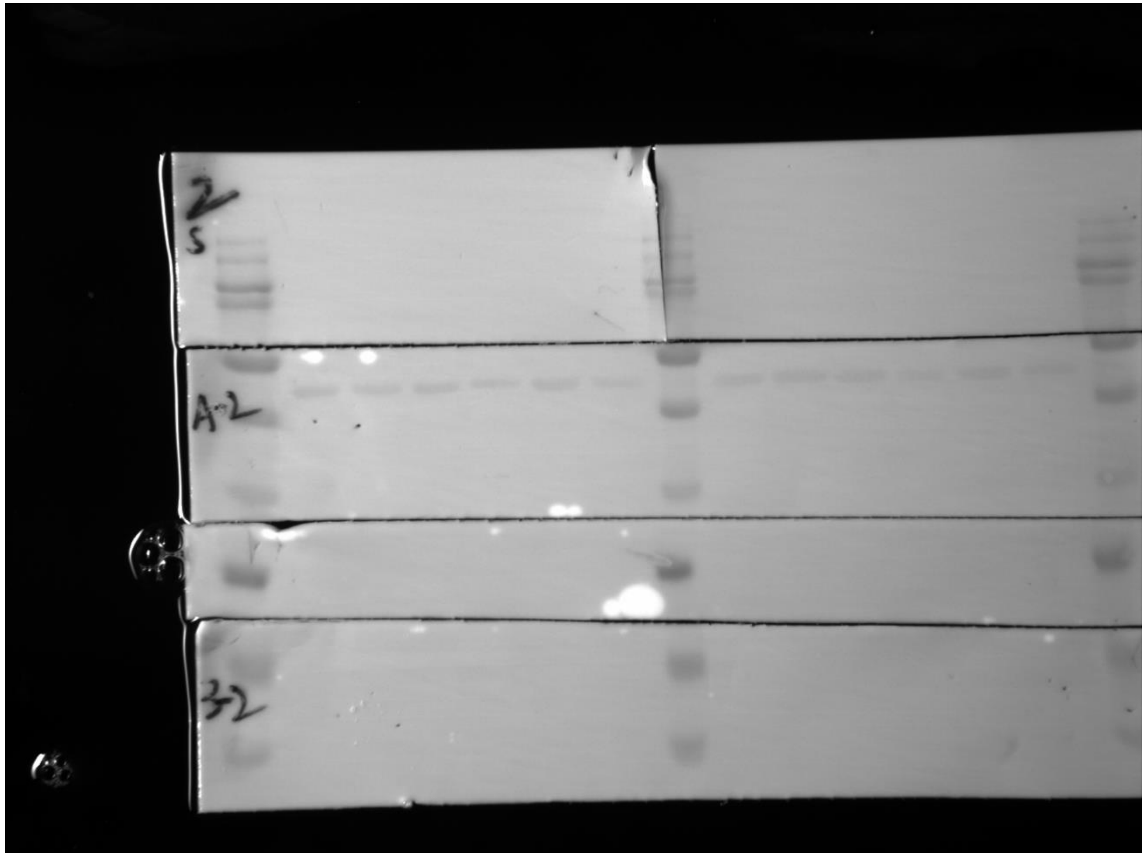

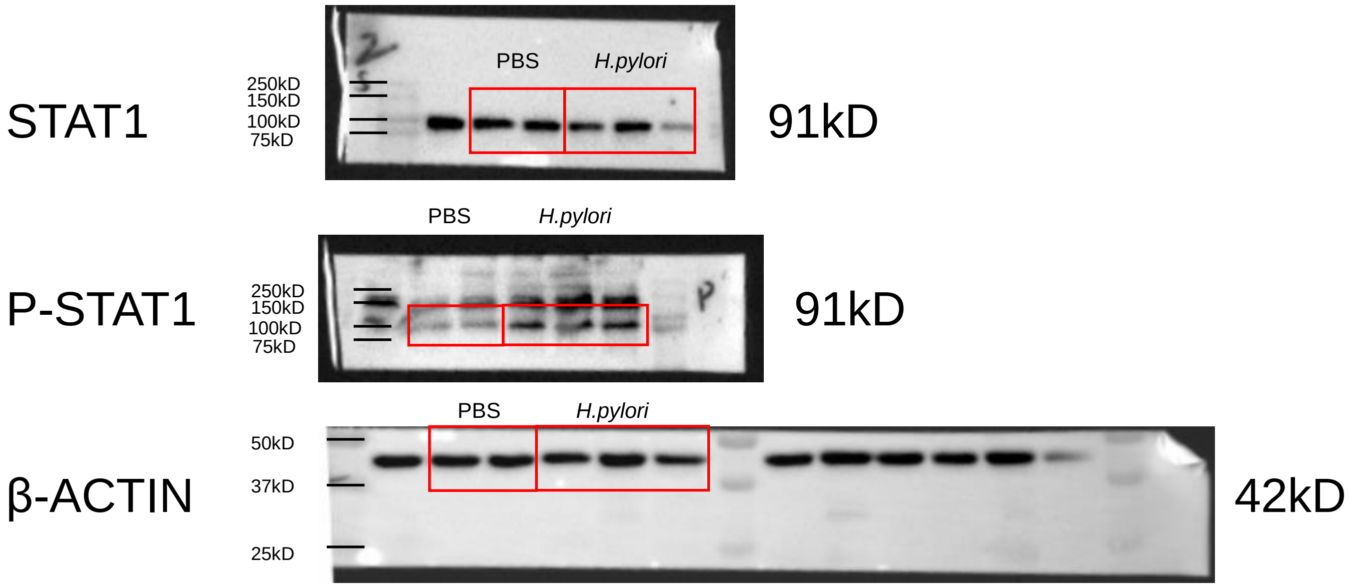

SP-Fig.6A

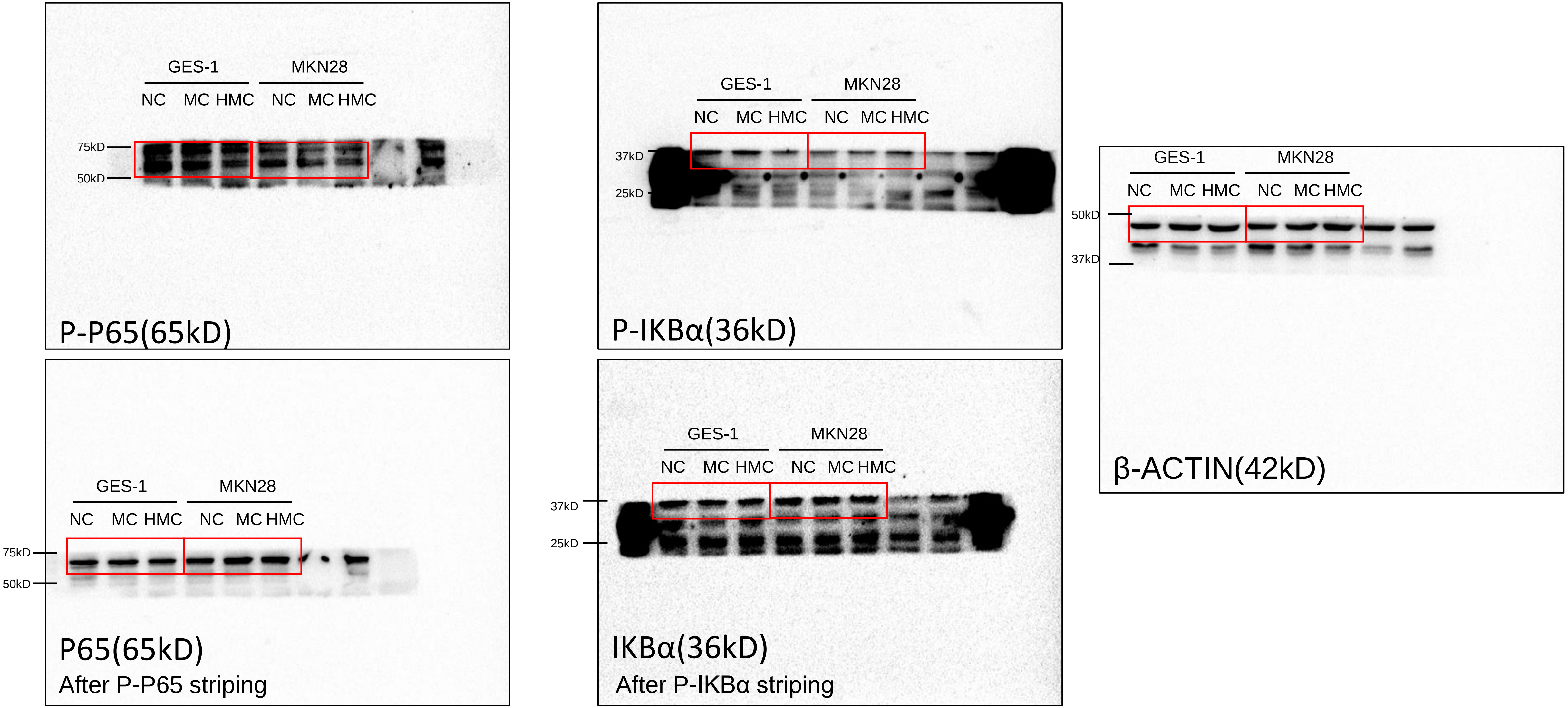
GES-1
MKN28
NC MC HMC NC MC HMC
75kD
50kD
P-P65(65kD)
37kD
25kD
P-IKBα(36kD)
50kD
37kD
β-ACTIN(42kD)
P65(65kD)
After P-P65 striping
IKBα(36kD)
After P-IKBα striping

SP-Fig.6B

P-P65

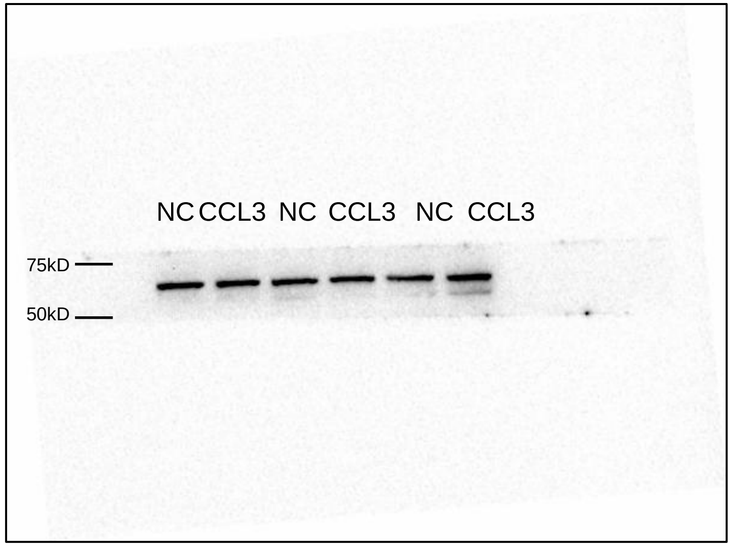

65kD

P65

After P-65 striping

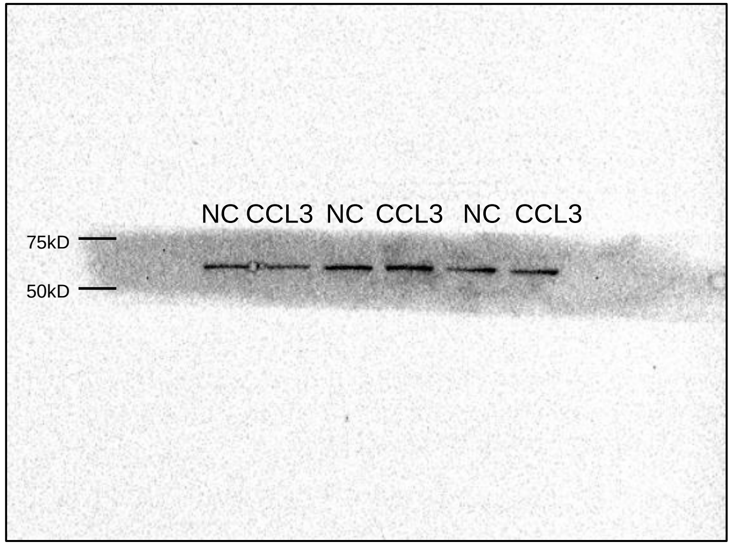

65kD

β-ACTIN

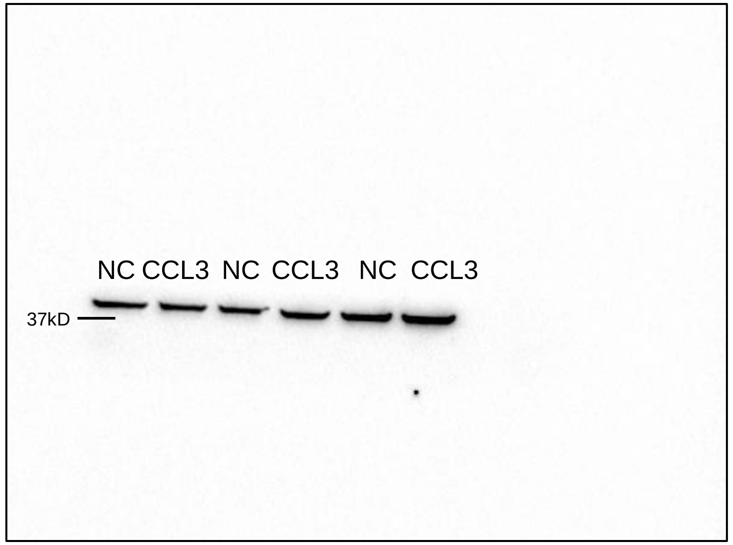

42kD

SP-Fig.6C

P-P65

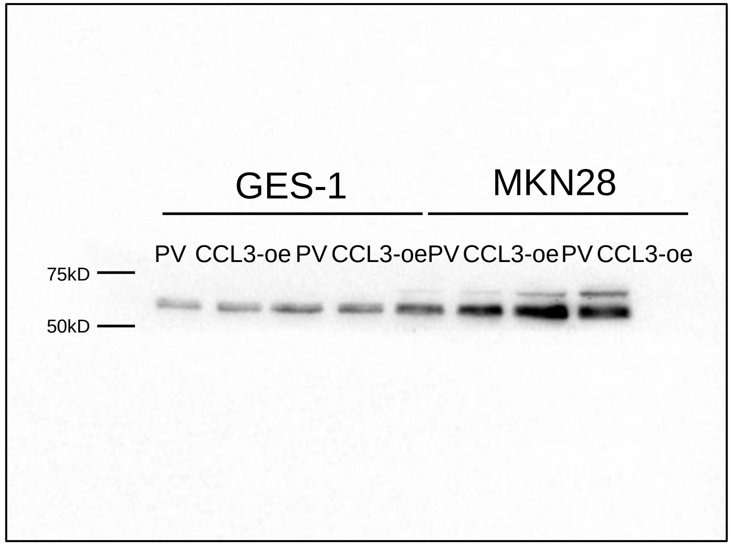

65kD

β-ACTIN

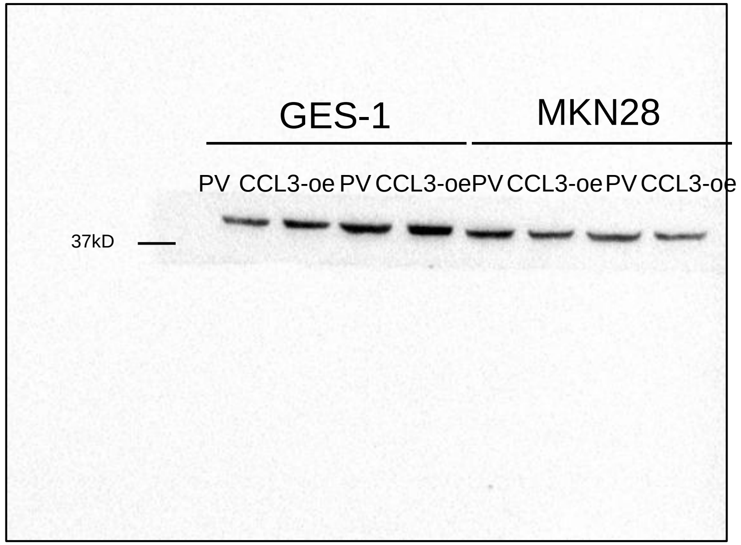

42kD
